# Rapid MRSA detection via tandem mass spectrometry of the intact 80 kDa PBP2a resistance protein

**DOI:** 10.1038/s41598-021-97844-w

**Published:** 2021-09-15

**Authors:** Jason R. Neil, Arvind Verma, Scott R. Kronewitter, William M. McGee, Christopher Mullen, Marjaana Viirtola, Annika Kotovuori, Herdis Friedrich, Johan Finell, Joni Rannisto, John E. P. Syka, James L. Stephenson

**Affiliations:** 1 Thermo Fisher Scientific, Cambridge, MA USA; 2 Thermo Fisher Scientific, Vantaa, Finland; 3 Thermo Fisher Scientific, San Jose, CA USA

**Keywords:** Microbiology, Bacteria, Clinical microbiology, Pathogens, Biological techniques, High-throughput screening, Mass spectrometry, Proteomic analysis, Diseases, Infectious diseases

## Abstract

Treatment of antibiotic-resistant infections is dependent on the detection of specific bacterial genes or proteins in clinical assays. Identification of methicillin-resistant *Staphylococcus aureus* (MRSA) is often accomplished through the detection of penicillin-binding protein 2a (PBP2a). With greater dependence on mass spectrometry (MS)-based bacterial identification, complementary efforts to detect resistance have been hindered by the complexity of those proteins responsible. Initial characterization of PBP2a indicates the presence of glycan modifications. To simplify detection, we demonstrate a proof-of-concept tandem MS approach involving the generation of N-terminal PBP2a peptide-like fragments and detection of unique product ions during top-down proteomic sample analyses. This approach was implemented for two PBP2a variants, PBP2a_mecA_ and PBP2a_mecC_, and was accurate across a representative panel of MRSA strains with different genetic backgrounds. Additionally, PBP2a_mecA_ was successfully detected from clinical isolates using a five-minute liquid chromatographic separation and implementation of this MS detection strategy. Our results highlight the capability of direct MS-based resistance marker detection and potential advantages for implementing these approaches in clinical diagnostics.

## Introduction

Antibiotic-resistant bacteria are a serious threat to human health around the globe^1^. The ability to track, prevent, and treat bacterial infections while maintaining good antibiotic stewardship has become a clinical and community health challenge. The prevalence and persistence of methicillin-resistant *Staphylococcus aureus* (MRSA) within hospital and community environments continues to be an important threat even as other resistance mechanisms evolve and emerge. MRSA strains often exhibit genetic heterogeneity, highlighting the diversity of lineages that have acquired resistance and virulence genes over time^2^. All strains are characterized by the presence and expression of the Staphylococcal cassette chromosome *mec* (SCC*mec*), a mobile genetic element containing genes enabling resistance to β-lactam antibiotics. Thirteen different SCC*mec* cassettes have been described to date, with the majority being characterized by the presence of the *mecA* gene^2^. The *mecA* gene encodes penicillin-binding protein 2a (PBP2a_mecA_), a membrane protein facilitating peptidoglycan biosynthesis and cell wall formation with low affinity for β-lactam antibiotics^3–5^. (Within the following text, use of the term ‘PBP2a’ will encompass all protein variants and specific variants will include the gene name in subscript). Where antibiotic-mediated inhibition of other penicillin-binding proteins (PBPs) would prevent cross-linking of cell wall peptidoglycans, expression of PBP2a enables this vital process, facilitating cell survival and growth. The less common and recently discovered genes of *mecB*, *mecC*, and *mecD* in three unique SCC*mec* cassettes encode variants of PBP2a_mecA_ exhibiting less than 70% amino acid homology^2,6–8^. The *mecC*-encoding PBP2a protein (PBP2a_mecC_, previously referred to as PBP2a_LGA251_) present in the SCC*mec* Type 11 SCC*mec* cassette lacks transglycosylation activity, may function with other glycosyltransferases to confer antibiotic resistance, and highlights the unique mechanisms that may characterize each variant^2^. PBP2a protein expression alone is not the sole determinant of MRSA strain susceptibility to β-lactam antibiotics as resistance phenotypes are dependent on the interplay of many factors including cell wall modifications, membrane microdomain structure, and the interplay of additional bacterial proteins and transcription factors^2,9,10^. Regardless, the detection of PBP2a has been the most reliable method for MRSA identification and a main strategy for many clinical testing approaches. Unfortunately, many current detection approaches are labor intensive, costly, rely on indirect detection, or lack specificity^11,12^.

Most MRSA testing strategies are either phenotypic or molecular in nature. Phenotypic approaches include the use of chromogenic agars that employ biochemical means of differentiating susceptible from resistant *S. aureus* colonies thru the uptake of media substrate(s) and resultant differences in colony color^11,13^. Examples of molecular assays include antibody-based direct detection of PBP2a_mecA_, as with latex agglutination or lateral flow immunochromatographic assays, or indirectly using polymerase chain reaction (PCR)-based approaches to detect the presence of the *mecA* gene or regulatory element^11,14,15^. Chromogenic agar detection approaches lack specificity, may produce ambiguous results, and requires additional culturing time crucial to clinical decision timelines. While sensitive and specific, many molecular assays can be difficult to automate, require additional consumables, may be susceptible to gene polymorphisms or mutations, and may require a significant amount of time to complete (e.g., ≥ 1 h)^11^.

Since the introduction of mass spectrometry (MS) systems into clinical microbiology laboratories, efforts have been made to use matrix-assisted laser desorption/ionization with time of flight (MALDI-TOF) MS-based systems to determine if *S. aureus* samples are antibiotic-resistant^16,17^. While routine MALDI-TOF MS-based species identification may be successful, this approach has struggled to provide strain discriminatory power and differentiate MRSA from methicillin-sensitive *S. aureus* (MSSA)^18^. Previous comparisons of MALDI-TOF MS-based peak profiles of oxacillin-resistant and -sensitive *S. aureus* strains with the same genetic background failed to identify reproducible diagnostic peaks^19^. Recent development of data processing algorithms to differentiate MRSA clonal lineages or implement decision-tree approaches to distinguish MSSA and MRSA MALDI-TOF peak profiles has shown promise and advance this approach^20,21^. Alternatively, coupling of MALDI-TOF *S.aureus* identification with separate identification of MRSA using a PBP2a_mecA_ latex agglutination assay is a solution for limited sample analysis but is reliant on assay reagents and sample processing^22^. Direct identification of MRSA through targeted detection of smaller proteins encoded on specific SCC*mec* cassettes has been successful in a limited number of cases (e.g., PSM-mec encoded on cassette types II, III, & VIII) but can be dependent on solvent extraction conditions^23–25^. These challenges highlight a need for an MS-based approach that is capable of directly detecting the PBP2a protein rather than relying on associations or secondary features. While high-resolution MS-based bottom-up detection of tryptic peptides (e.g., with MS an Orbitrap analyzer) for antibiotic resistance detection is straightforward, this approach is time-consuming, labor-intensive, and costly; thus precluding its adoption as a viable clinical diagnostic strategy^26^. A detailed comparison MALDI-TOF and high-resolution MS technology and concepts are beyond the scope of this article and readers are referred to a representative selection of publications on this topic^27–30^.

Top-down high-resolution MS detection of intact proteins represents an alternative antibiotic resistance identification process with less sample processing and a corresponding reduction in time-to-results. Previous detection and characterization of bacterial virulence factors using top-down proteomic methods have demonstrated the application of these detection methods for bacterial protein characterization^31^. Improvements in MS instrumentation, analytical methods, and data processing are at point where this analytical approach can also be implemented for high resolution MS bacterial identification and differentiation of closely related species^32–34^. For example, detection of bacterial proteins and differentiation of closely related species with liquid extraction surface analysis (LESA) followed by high-resolution MS detection offer a unique approach with minimal sample processing time and greater discriminatory power^35,36^. Application of top-down high-resolution MS detection of intact proteins and confident identification through fragmentation strategies has previously been demonstrated as a reliable and accurate method for resistance detection in other bacterial species^37,38^. In an expansion of our effort to characterize and develop MS-based resistance marker detection approaches, we propose a targeted method for direct detection of PBP2a through the implementation of intact protein liquid chromatographic (LC) separation and high-resolution MS for MRSA identification. In the development of the detection method, we confirm the presence of N-terminal methionine formylation and evidence for multiple glycosylations, a previously unknown modification on the PBP2a_mecA_ protein^39^. The approach to PBP2a_mecA_ detection, in the absence of induction, is scaled down to a five-minute LC–MS/MS method to detect smaller fragments of the intact protein and validated with clinical patient and quality control (QC) MRSA and MSSA strains. These results highlight the advantage of antibiotic resistance identification through direct detection of those proteins responsible, has the potential to improve and automate clinical workflows, providing a level of MS-based discrimination not previously available in this area of disease management.

## Results

### Detection and fragmentation of intact PBP2a_mecA_ protein

**Figure 1:**
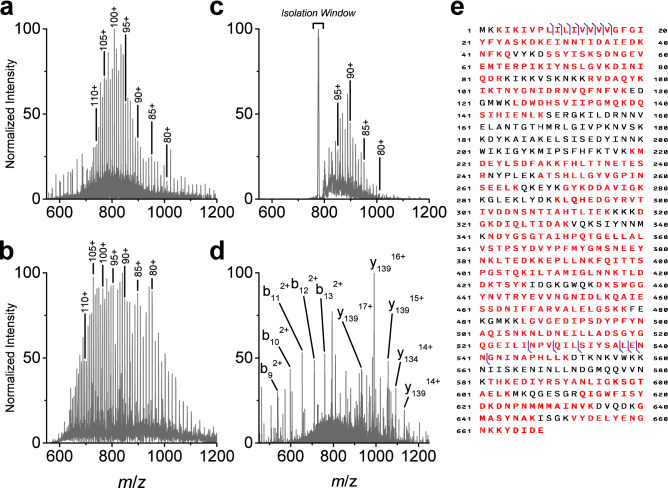
Identification of PBP2a_mecA_ protein from MRSA cell extract by LC–MS/MS. (**a**) Intact wild-type PBP2a_mecA_ MS spectrum from a representative MRSA strain (ATCC 33,591) separated by LC. Intact protein precursor ion charge states are labelled from 80 to 110. Data are representative for multiple MRSA strains (n ≥ 3). (**b**) Recombinant His_6_-PBP2a_mecA_ MS spectrum acquired using direct infusion. Identical precursor ion charges states are labelled as wild-type PBP2a_mecA_ spectrum. (**c**) Intact wild-type PBP2a_mecA_ MS spectrum produced during LC separation of cell extract and PTCR-mediated separation of superposed protein ion populations. Isolation window was centered at *m*/*z* 777 with a width of 5 *m*/*z*. Precursor ion charge states are labelled from 80 to 95. Data are representative of multiple technical replicates from the same MRSA cell extract (ATCC 33,591). (**d**) MS/MS spectrum of intact wild-type PBP2a_mecA_ precursor ion at *m*/*z* 793 (charge state = 102), using 1.5 *m*/*z* isolation window and fragmented with an HCD collision energy of 10 eV. Abundant N-terminal (*b*-ions) and C-terminal (*y*-ions) fragment ions are labelled. Fragmentation data are representative for multiple charge states and different MRSA strains (n ≥ 100). (**e**) Associated b- and y-ion fragment location for MS/MS of intact wild-type PBP2a_mecA_ protein indicated by purple vertical lines. Red colored amino acids indicate protein coverage (72.2%) for complementary bottom-up peptide analysis and protein characterization. Protein coverage associated with peptide data are from multiple analyses (n = 12).

MRSA identification through direct PBP2a detection required initial isolation and characterization of the resistance marker protein. Previous biochemical and structural characterization studies, relying on soluble recombinant derivatives and overlooking potential post translational modifications, have assumed the predicted mass of PBP2a_mecA_ (UniProtKB Q53707 or NCBI RefSeq NG_047936) to be approximately 76 kDa^5,39,40^. However, separation and detection of the intact wild-type protein by LC–MS indicates that the mass is higher, at approximately 80 kDa (Fig. 1a). Information on the observed PBP2a_mecA_ protein precursor ion distribution is provided in Supplemental Table S1. When analyzed by LC–MS, the same charge state ions of recombinant His_6_-PBP2a_mecA_ (note: full amino acid His_6_ tag leader sequence was unknown) were observed at lower *m*/*z* values and the intact mass was approximately 76 kDa, as previously reported (Fig. 1b)^39^. For example, the wild-type and recombinant precursor ions bearing a charge state of +102 could be observed at *m*/*z* 793.0142 and *m*/*z* 742.4749, respectively. A list of the observed recombinant protein precursor ions is provided in Supplemental Table S2. Characteristic for both wild-type and recombinant protein spectra was the high charge state pattern, a consequence of the large number of basic residues present within the PBP2a_mecA_ amino acid sequence. Confirmation of the wild-type target was observed in LC separated MRSA samples subject to gas phase ion-ion proton-transfer charge reduction (PTCR) and MS detection; a procedure developed for gas-phase separation of multiply charged protein ion populations and analyses of complex samples (Fig. 1c)^41,42^. Due to the high density of peaks observed in the single stage MS analysis of intact complex protein extracts, PBP2a_mecA_ was often difficult to detect. The implementation of PTCR enabled its separation from superposed abundant protein isotope distributions having slower reaction kinetics. As proteins were separated by LC, charge reduction was performed on sequentially isolated ions. This facilitated the detection of numerous high mass proteins, of which only one resembled our initial 80 kDa observation. A list of observed PBP2a_mecA_ precursor ions produced by PTCR is provided in Supplemental Table S3. Isolation and tandem MS (MS/MS) using high-energy collisional dissociation (HCD) of these precursor ions yielded matching N-terminal and C-terminal product ions confirming the target to be PBP2a_mecA_. A representative fragmentation spectrum for a single PBP2a_mecA_ precursor at *m*/*z* 793.0 is displayed in Fig. 1d. Based on a + 27.9949 Da mass shift observed in the N-terminal product ions (i.e., b_9_ to b_16_ fragments), it was postulated that PBP2a_mecA_ retains its formylated N-terminal methionine. Confirmation of this modification was performed with synthetic peptides in the results presented below. The observed C-terminal product ions, from amino acids 527 to 668, matched predicted fragment masses and indicated the absence of modifications on this region of PBP2a_mecA_. A total of 16 unique matching protein fragments could be identified in MS/MS spectra, accounting for approximately 2% of the cleavable bonds (Fig. 1e, ProSight P-Score = 3.6e^−30^, Supplemental S4). The limited HCD fragmentation of PBP2a_mecA_ was not unexpected given its molecular weight and associated high charge state distribution produced by electrospray ionization^43,44^. A full list of observed MS/MS product ions is provided in Supplemental Table S4. The C-terminal fragmentation pattern observed in wild-type PBP2a_mecA_ could be reproduced with the recombinant protein despite lacking three C-terminal amino acids and is displayed in Supplemental Fig. S1. Examination of other MRSA isolates expressing PBP2a_mecA_ using the same precursor ion fragmentation list confirmed the approach was applicable across multiple strains and the discrepancy in predicted PBP2a_mecA_ mass was common to all isolates.

The difference between the predicted and observed mass for PBP2a_mecA_ was further investigated. Staining of immunoprecipitated PBP2a_mecA_ from MRSA protein extracts separated by SDS-PAGE revealed that the protein is glycosylated, as indicated by a fluorescent band at ~ 75 kDa using the ProQ Emerald stain (Supplemental Fig. S2). Following the treatment of immunoprecipitated PBP2a_mecA_ with a deglycosylation enzyme mixture, glycoprotein staining was absent. Similar reactivity was observed with the antibody heavy chain band at 50 kDa in the immunoprecipitated extract. Conversely, the recombinant version did not show glycoprotein stain reactivity under any treatment conditions. Protein location was further confirmed with Sypro Ruby and silver staining.

In-gel enzymatic digestion or LC–MS/MS analysis of PBP2a_mecA_-enriched samples identified 51 unique PBP2a_mecA_ peptides (Supplemental Table S5, Supplemental Figs. S3.1–S3.52), with protein sequence coverage of approximately 72.2% (indicated in red in Fig. 1e). Based on the combined information from top-down and bottom-up PBP2a_mecA_ analyses, post-translation modifications (PTMs) on the protein are expected to be exist between amino acids 80 and 245. Further investigation of these uncharacterized regions of the protein should yield information related to glycan modification.

### Detection of PBP2a_mecA_ by In-Source Fragmentation

**Figure 2:**
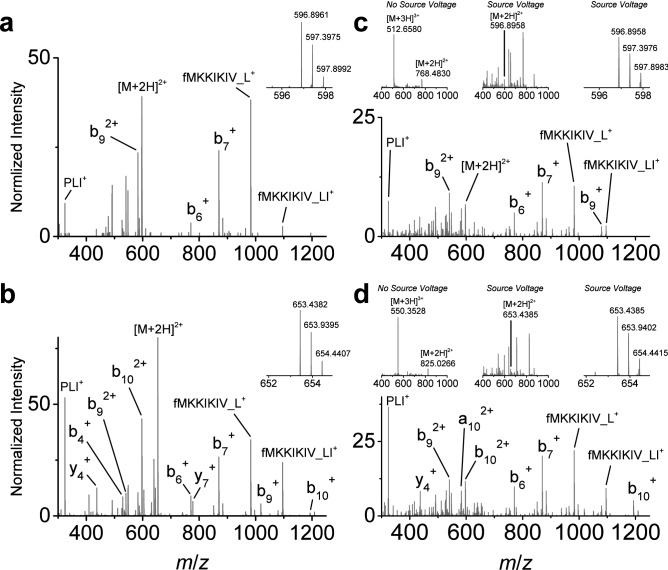
PBP2a_mecA_ protein detection using in-source generated peptide-like fragments and MS/MS fragmentation. **(a**, **b**) Source-induced dissociated N-terminal peptide-like fragments from PBP2a_mecA_ (ATCC MRSA isolate BAA-44) at precursor *m*/*z* 596.8961 (a) and *m*/*z* 653.4382 (**b**) for selected ion monitoring (insets) and associated representative targeted MS/MS spectra (n ≥ 100). (**c**) Synthetic peptide confirmation of source-induced dissociated peptide-like precursor *m*/*z* 596.8961 and associated MS/MS spectrum (inset spectrum series displays peptide spectrum without in-source energy, with in-source dissociation, and magnified spectrum of target precursor following in-source dissociation). (**d**) Synthetic peptide confirmation of source-induced dissociated peptide-like precursor *m*/*z* 653.4382 and associated MS/MS spectrum (inset spectrum series displays peptide spectrum without in-source energy, with in-source dissociation, and magnified spectrum of target precursor following in-source dissociation). Data collection for synthetic peptides was repeated multiple times on different MS instruments (n = 3).

The development of an LC protein separation method adequate for clinical assay throughput requirements (i.e., ≤ 5 min) led to the observation that PBP2a_mecA_ MS/MS product ions were difficult to detect with shorter separations. While fractionation and multidimensional LC approaches afford greater protein separation and MS characterization opportunities in longer discovery-based proteomic investigations, these methods are not suitable for clinical assays. With little chromatographic separation, the prohibitive effects of co-isolating multiple proteins, poor ionization characteristics, low cellular abundance, and interfering matrix ionization properties were readily observed. Informed by the HCD fragmentation of intact PBP2a_mecA_, a detection approach was devised for those abundant product ions generated through an alternative method. Previous N- and C-terminal protein sequencing of moderate to high mass proteins by MS using in-source fragmentation approaches has facilitated the identification of difficult to characterize proteins^45,46^. In our experience, this approach has generated sequence-specific and reproducible fragmentation for proteins lacking disulfide bonds. In-source fragmentation followed by tandem MS enabled a similar protein characterization approach following ionization of the intact PBP2a_mecA_ molecule. The increase in voltage (i.e., increase S-lens voltage) at the inlet of the mass spectrometer, normally adjusted to improve the desolvation of intact proteins, may also lead to their dissociation at higher voltages and is analogous to non-specific protein digestion. The use of this approach provided enough energy to break apart the intact PBP2_mecA_ protein into smaller pieces, enabling easier isolation from interfering analytes, and their subsequent fragmentation for sequence analysis. While both protein termini could be detected in this manner, only the N-terminus yielded reliable abundant peptide-like dissociated product ions. These target precursor ions were also the same N-terminal fragments observed in the HCD fragmentation of intact PBP2a_mecA_. Of the multiple N-terminal peptide-like fragments, the selected targets for our method included fMKKIKIVPLI (MH^2+^ = *m*/*z* 596.8961, b_10_^2+^) and fMKKIKIVPLIL (MH^2+^ = *m*/*z* 653.4382, b_11_^2+^), where ‘f’ indicates formylation of the methionine amino acid residue. Targeted isolation and fragmentation of these N-terminal peptide-like ions generated product ions in MS/MS spectra that were easily identified (Fig. 2a,b). However, two product ions were unexpected at *m*/*z* 982.6482 and *m*/*z* 1095.7322. Verification of the observed product ions for both N-terminal source-induced dissociated PBP2a_mecA_ peptide-like targets were confirmed with synthetic analogs (Fig. 2c,d). The process for generating each in-source peptide-like fragment is displayed in the upper sequence of spectra in Fig. 2c,d (e.g., MS of original synthetic peptide, MS of in-source dissociation of synthetic peptide highlighting location of target peptide-like precursor ion and zoomed in MS of in-source dissociated target peptide-like precursor ion). The unique product ions, labeled fMKKIKIV_L^+^ and fMKKIKIV_LI^+^, were present in the synthetic analog fragment spectra and were the result of gas phase peptide cyclic rearrangement; a process previously reported for peptide b ions^47,48^. This rearrangement process is illustrated in Supplemental Fig. S4. The specificity of the PBP2a_mecA_ peptide-like targets was addressed within the context of previous identification of *S. aureus*. When searched against an *S. aureus* reference protein database (taxid:1280, refseq protein) using the Basic Alignment Search Tool (BLAST) and blastp algorithm, the source-induced PBP2a_mecA_ protein fragment sequences provide an E-value score of 6e^−4^ for ‘MKKIKIVPLI’ and 7e^−5^ for ‘MKKIKIVPLIL’ (using https://blast.ncbi.nlm.nih.gov/Blast.cgi). These scores do not take into consideration the sequence fragment rearrangement phenomenon and N-terminal formylation as unique identifiers. Given that these peptides are generated during two dissociation steps, the probability of generating the associated product ions by chance is extremely unlikely. Consequently, these targets provide unique markers for PBP2a_mecA_ detection. Implementation of this detection approach with PBP2a_mecA_-enriched samples exhibited multiple chromatographic peaks indicating multiple PBP2a_mecA_ glycoforms are present (Supplemental Fig. S5).

### Detection of PBP2a_mecC_ by in-source fragmentation

**Figure 3:**
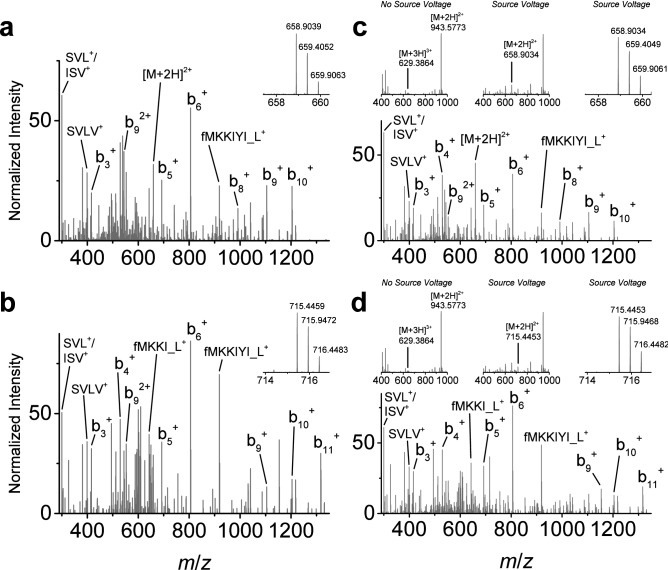
PBP2a_mecC_ protein detection using in-source generated peptide-like fragments and MS/MS fragmentation. (**a**, **b**) Source-induced dissociated N-terminal peptide-like fragments from PBP2a_mecC_ (ATCC MRSA isolate BAA-2312) at precursor *m*/*z* 658.9042 (**a**) and *m*/*z* 715.4462 (**b**) for selected ion monitoring (insets) and associated representative targeted MS/MS spectra (n ≥ 100). (**c**) Synthetic peptide confirmation of source-induced dissociated peptide-like precursor *m*/*z* 658.9042 and associated MS/MS spectrum (inset spectrum series displays peptide spectrum without in-source energy, with in-source dissociation, and magnified spectrum of target precursor following in-source dissociation). (**d**) Synthetic peptide confirmation of source-induced dissociated peptide-like precursor *m*/*z* 715.4462 and associated MS/MS spectrum (inset spectrum series displays peptide spectrum without in-source energy, and magnified spectrum of target precursor following in-source dissociation). Data collection for synthetic peptides was repeated multiple times on different MS instruments (n = 3).

Using the same source-induced dissociation approach, the detection of N-terminal peptide-like fragments for a MRSA strain expressing PBP2a_mecC_ was performed, assuming similar N-terminal protein methionine formylation and without previous MS detection of the intact protein. While multiple N-terminal peptide-like targets could be detected based on the PBP2a_mecC_ amino acid sequence, two N-terminal fragment ions for fMKKIYISVLVL (MH^2+^ = *m*/*z* 658.9042, b_11_^2+^) and fMKKIYISVLVLL (MH^2+^ = *m*/*z* 715.4462, b_12_^2+^) were reliably detected (Fig. 3a,b). Source-induced dissociation, isolation, and fragmentation of these N-terminal peptide-like fragments, following LC separation and ionization of the intact PBP2a_mecC_ protein, produced matching product ions as well as two product ions resulting from peptide rearrangements. These product ions are labeled fMKKI_L^+^ at *m*/*z* 642.4007 and fMKKIYI_L^+^ at *m*/*z* 918.5481. An example of PBP2a_mecC_ N-terminal peptide rearrangement is illustrated in Supplemental Fig. S6. A similar BLAST evaluation for these peptide sequences generated E-value scores of 7e^−5^ for ‘MKKIYISVLVL’ and 7e^−6^ for ‘MKKIYISVLVLL’. Verification of the N-terminal PBP2a_mecC_ peptide-like precursor targets and associated fragmentation was also confirmed with a single synthetic peptide analog (Fig. 3c,d). Observation of the peptide-like sequence rearrangements was also confirmed with this analog. Thus, use of the in-source dissociation approach is not unique to one PBP2a variant and can also be used for PBP2a_mecC_ detection.

### Extensibility of MRSA detection approach

**Figure 4:**
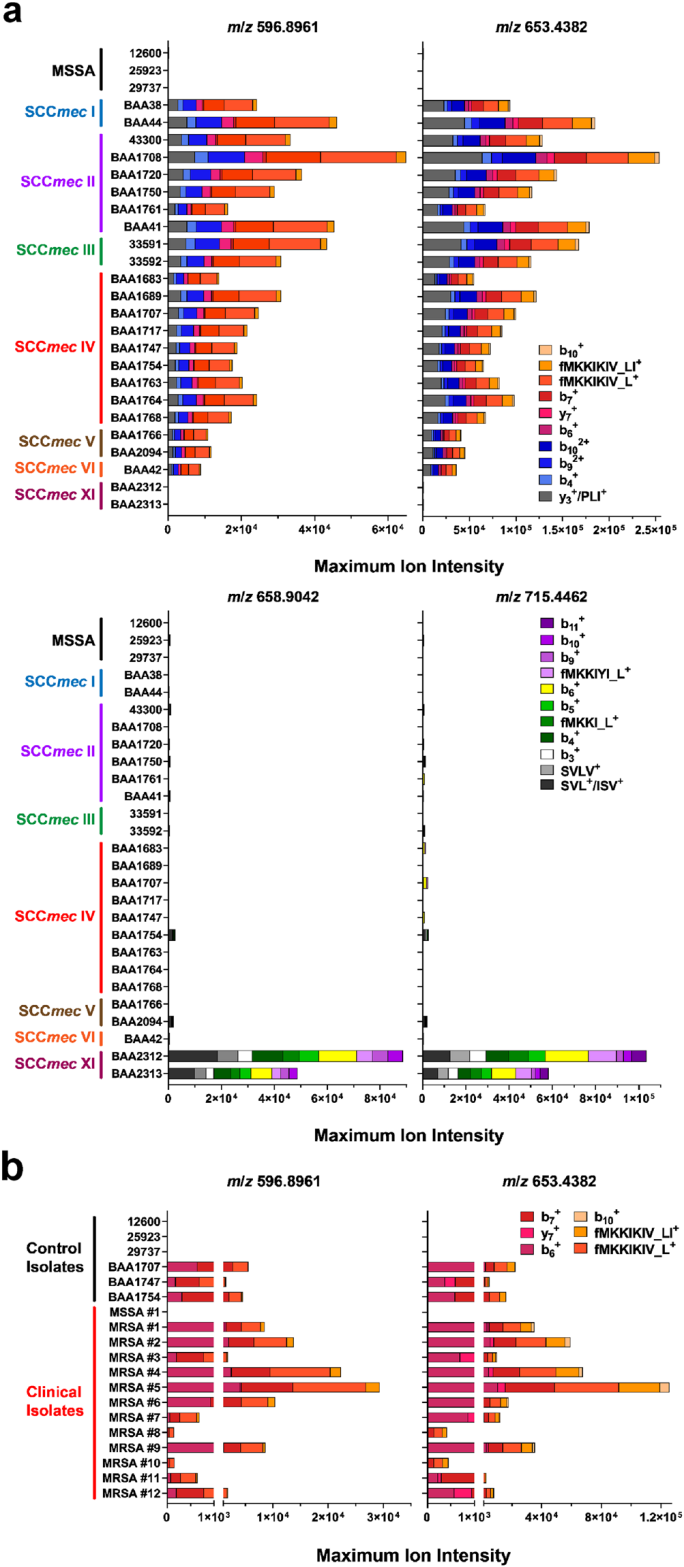
Evaluation of source-induced peptide detection of PBP2a across representative MRSA panel and clinical isolates. (**a**) Performance of source-induced detection method for PBP2a_mecA_ (top panel) and PBP2a_mecC_ (bottom panel) N-terminal peptide-like fragments for MRSA strains exhibiting different SCC*mec* and PFGE genetic backgrounds, as well as negative MSSA isolates (listed in Supplemental Table S6). Strains were cultured on TSA plates and harvest after overnight growth. Colored bars represent the mean maximum intensity for each product ion over a 60-min LC protein separation. Results are from distinct biological replicates (n = 3). (**b**) Performance of MSSA and MRSA quality control strains along with clinical isolates over five-minute PBP2a_mecA_ detection method using custom SPE trap and source-induced dissociation method. Strains were cultured on Blood Agar plates and harvested after overnight growth. Data are the mean maximum ion intensity for each product ion and calculated from distinct biological replicates (n = 2 or 3).

Evaluation of the source-induced dissociation methods for PBP2a_mecA_ and PBP2a_mecC_ targets were performed across a representative genetically diverse group of validated SCC*mec*-expressing and pulsed-field type MRSA strains (Fig. 4a). Included in this examination were three MSSA strains as negative controls. The observed maximum intensity for each target’s product ions greater than *m*/*z* 300 (i.e., those fragments consisting of two or more amino acids) within a 10 ppm tolerance window were monitored during a 60 min LC–MS/MS analysis and averaged for three separate experiments. The results of this analysis confirmed the specificity of the approach for each set of PBP2a_mecA_ and PBP2a_mecC_ precursors, as no false-positives were observed in the MSSA strains or false-negatives observed with either method when MRSA strains were tested. All MRSA isolates with SCC*mec* cassettes carrying the *mecA* gene displayed positive results for the PBP2a_mecA_ precursors *m*/*z* 596.8961 and *m*/*z* 653.8961. The two MRSA isolates harboring the SCC*mec* Type XI cassette (carrying the *mecC* gene) were the only to exhibit strong positive results for PBP2a_mecC_ precursors, *m*/*z* 658.9042 and *m*/*z* 715.4462. While some non-specific signals for PBP2a_mecC_ product ions were observed in PBP2a_mecA_-expressing strains, further inspection revealed that some of the product ions were not the correct charge state or too low a signal to be considered true positives (i.e., at the level of noise).

### Proof-of-concept MRSA detection with online solid phase extraction

Adapting this PBP2a detection approach to a rapid clinical assay (≤ 5 min) for MRSA identification involved the design and implementation of a single-use solid phase extraction (SPE) reverse-phase monolith tip (Supplemental Fig. S7) that could be coupled in-line with a modified nano-LC system for proof-of-concept protein separation and detection^49^. A five-minute LC method was used to elute *S. aureus* proteins bound to the SPE tip from crude cell extracts and coupled with the source-dissociation method for N-terminal PBP2a_mecA_ peptide-like fragment detection. A representative extracted ion chromatogram for PBP2a_mecA_ source-induced N-terminal peptide-like fragment transition ions display the elution profile of the protein produced with this method in Supplemental Fig. S8. Independent confirmation of PBP2a_mecA_ protein expression in clinical isolates was also performed with a lateral flow clinical assay. Only those target product ions with *m*/*z* values greater than 700 within a 10 ppm tolerance window were considered and averaged for three separate experiments. With shorter protein LC separations, all product ions exhibited lower maximum ion intensity and those with less specificity within the lower *m*/*z* range were excluded (i.e., resulting in their omission from the observation list). Successful detection of PBP2a_mecA_ N-terminal peptide-like product ions for both precursors were observed for MRSA QC isolates (n = 3 isolates) and clinical samples (n = 12 isolates) from cystic fibrosis patients (Fig. 4b). No false-positive results were observed with MSSA QC (n = 3 isolate) or clinical (n = 1 isolate) isolates.

Over the course of developing this analytical method, we estimate the false-negative rate at less than five percent for MRSA strains expressing PBP2a_mecA_ (e.g., 49/51 MRSA strains were correctly identified). This observation is based on repeated testing and changing of the MS acquisition parameters prior to the analysis of the data presented here. This preliminary performance result is based on a minimum threshold of three or more product ions for the PBP2a_mecA_ N-terminal peptide-like fragments within a 10 ppm mass tolerance. These results demonstrate that direct MS-detection of PBP2a and identification of MRSA can be achieved on a short analysis timescale and provide actionable information.

## Discussion

The identification of PBP2a as a putative glycoprotein adds to the growing list of similarly modified *S. aureus* proteins that include plasmin-sensitive protein (Pls), the serine-rich adhesion for platelets (SraP), and serine-rich *S. aureus* clumping factor A (ClfA)^50,51^. Altered MRSA cell wall glycosylation is involved in pathogenesis and immune evasion but the role this modification plays in PBP2a_mecA_ remains an open question^52^. It is unknown if glycan modifications exist on other PBP2a variants, if these modifications are similar, or whether they are the variable across different species or strains expressing this resistance marker protein. Further characterization may yield unique diagnostic information for MRSA identification and differentiation between species and strains. Given the complexity of protein glycosylation, this post translation modification complicates the straightforward MS detection of the PBP2a protein. Previous characterization of small bacterial glycoproteins using electron-transfer dissociation (ETD) generated sufficient fragmentation and sequence coverage, without loss of labile glycans, to accurately assign associated amino acid modification sites^31^. Fragmentation of intact PBP2a_mecA_ using ETD was not considered given its large size, charge state distribution, and number of dissociation channels that would result in product ions with low signal-to-noise. The combination of ionization and/or dissociation approaches described above and elsewhere (i.e., PTCR, HCD, ETD, or AI-ETD) with smaller fragments of PBP2a_mecA_ may enable sufficient coverage to accurately determine its associated glycosylation sites.

Accurate and reliable detection of diagnostic proteins is a necessary requirement for MS-based clinical microbial identification^17^. The same should be true for MS-based antibiotic resistance characterization. Indirect detection approaches are often limited in their application and fail under robust independent evaluation, as with the detection of unique SCC*mec* cassette proteins that are often specific for a subset of MRSA strains^2,24^. The ability to provide a rapid MS-based test for PBP2a detection has the potential to improve MS diagnostics and antibiotic resistance characterization in clinical microbiology. We demonstrate the feasibility of an LC–MS/MS approach to MRSA identification with direct PBP2a detection. The results of this approach were consistent with current ‘gold standard’ approaches for MRSA detection (i.e., pcr-based detection). Source-induced dissociation and detection of two N-terminal peptide-like fragments specific to PBP2a_mecA_ and PBP2a_mecC_ can be used for MRSA identification during short LC separation of microbial intact protein extracts. This approach is not limited to the LC method detailed here but could performed with a diverse range of LC configurations. The ability to create diagnostic source-induced fragment ions in a lower *m*/*z* range with fewer analyte or contaminant ion interferences enables the detection of the PBP2a target on short LC protein separations. While MRSA strains expressing PBP2a_mecB_ and PBP2a_mecD_ were unavailable to test in this study, we anticipate the same detection approach would be successful for those variants. Each of the four variants have unique N-terminal amino acid sequences (Supplemental Table S7) that would enable their differentiation based on a distinct set of source-induced peptide-like fragment and product ions. Based on the observations of PBP2a_mecA_ and PBP2a_mecC_, N-terminal methionine formylation is likely to be present in all variants. The presence and retention of an N-terminal formylated methionine in PBP2a_mecA_ and PBP2a_mecC_ is most likely explained by the competition of binding proteins (e.g., signal recognition particle) with peptide deformylase during protein translation and membrane translocation, similar to other bacterial membrane proteins^53^. The product ions associated with the cyclic rearrangement of the PBP2a_mecA_ and PBP2a_mecC_ N-terminal fragments create additional diagnostic power that would not have been otherwise predicted. Cyclic rearrangement of N-terminal source dissociation-induced fragments for PBP2a_mecB_ and PBP2a_mecD_ and their associated product ions would also be anticipated. When considering the unique observations listed above, it is unlikely that one would detect any of combined product ions by chance for a specific PBP2a target. This is illustrated by the lack of any observable target products ion for PBP2a_mecA_ in the MSSA strains evaluated. This detection approach would also be unaffected by amino acid substitutions in the downstream PBP2a_mecA_ protein sequence. Most substitutions appear in the allosteric and transpeptidase domain, either increasing or decreasing antibiotic resistance, while the N-terminal amino acid sequence of PBP2a_mecA_ appears conserved across evolution^54–56^. Additional amino acid substitutions, variations in the glycosylation status, or other modifications also would not affect this detection approach. Therefore, this MRSA detection strategy is comprehensive for most MRSA yet specific enough to detect variants of PBP2a protein.

When clinical isolates of MSSA and MRSA were tested with this MS-based method, all resistant strains were successfully detected using PBP2a_mecA_ in-source dissociated peptide-like product ion targets and all sensitive strains were negative for the same identifiers. Compared to other methods, the MS-based MRSA detection approach represents a significant improvement in automation when coupled with routine MS-based microorganism identification without the need for a separate workflow for resistance characterization.

As the *mecA* resistance gene is not unique to MRSA (e.g., present in methicillin-resistance *S. epidermidis* (MRSE)), this detection approach could also apply to the detection of β-lactam resistance in other species expressing the same PBP2a protein. The use of this approach may not be applicable for less common borderline oxacillin-resistant *S. aureus* (BORSA) or modified *S. aureus* (MODSA) strains possessing unique resistance mechanisms, but their further characterization may provide additional diagnostic marker(s) for MS-based identification^57^. For example, the detection of changes associated with increased PBP3 or PBP4 expression may be feasible with this or similar approaches and enable their identification^57^. Finally, this approach could also be adopted for other large bacterial proteins that contribute to antibiotic resistance in other gram-negative or gram-positive species.

The implementation of this proof-of-concept MS-based approach for MRSA identification has the potential to improve clinical diagnostic workflow efficiency, accuracy, and time-to-results as bacterial identification and resistance determination would be achieved on the similar time scales. With further efforts, these approaches will better our response to the control of bacterial infections and antibiotic stewardship.

## Methods

### Bacterial culturing and reagents

Laboratory and clinical MSSA and MRSA strains and their sources are listed in Supplemental Table S6. Briefly, characterized strains were purchased from American Type Tissue Culture Collection (ATCC) while clinical isolated were kindly provided by microbiology research and hospital labs. Following the receipt of bacterial isolates, *mecA* gene expression was confirmed by pcr analysis and immunochromatographic-based PBP2a_mecA_ protein detection. Cultures were maintained on Tryptone Soy Agar (TSA) or Blood Agar (TSA supplemented with 5% sheep blood) under aerobic conditions at 35 °C, unless otherwise indicated. Positive selection of antibiotic-resistant colonies was performed on agar plates with 1 μg Oxoid oxacillin antimicrobial susceptibility disks (Thermo Fisher Scientific, Waltham, MA). Liquid cultures were grown in 500 mL of Tryptone Soy Broth (TSB) supplemented with 2.5 μg/mL of oxacillin for 20 h at 35 °C in an orbital shaker, following the addition 1 mL of starter culture grown in TSB without antibiotics. All culturing reagents were purchased from Thermo Fisher Scientific (Waltham, MA) while oxacillin antibiotic for liquid cultures was purchased from MilliporeSigma (Burlington, MA). Recombinant PBP2a_mecA_ (230–00,051, N-terminal His_6_-tagged, amino acids 19–668) was purchased from Raybiotech (Palmtree Corners, GA). Protein Deglycosylation Mix was purchased from New England Biolabs (Ipswich, MA). Synthetic peptides for PBP2a_mecA_ (fMKKIKIVPLIDPL-OH and fMKKIKIVPLILDPL-OH) and PBP2a_mecC_ (fMKKIYISVLVLLLDPL-OH)) were purchased from New England Peptide (Gardner, MA).

### *S. aureus* protein extraction: agar cultures

Two different lysis approaches were employed for cultures grown on agar plates. MRSA colonies were collected and combined from a single 100 mm agar plate and suspended in 1 mL of chaotropic solvent (6 M guanidine hydrochloride, 250 mM Tris pH 7.2), transferred to tubes containing Matrix B lysing beads beating (MP Biomedicals, Irvine CA), and mechanically lysed using the Fisherbrand Bead Mill 4 Homogenizer (Thermo Fisher Scientific, Waltham MA). Alternatively, 3–5 MRSA colonies were collected with a 1 mm loop tool, suspended in a formic acid/acetonitrile solution (50% formic acid, 25% acetonitrile, and 25% water), and mixed manually by pipette. Extracts from the second approach were pelleted by centrifugation and then diluted with a 50% acetonitrile solution.

### MRSA protein extraction and enrichment: liquid cultures protein

Liquid cultures were pelleted by centrifugation, washed with phosphate buffered saline (pH 7.4), and then frozen prior to processing. Gentle lysis and suspension of soluble proteins was performed on ice (Lysis Buffer: 50 mM HEPES, 100 mM NaCl, 1 mM EDTA, 0.5% SDS, 1 mM EDTA, and 1 mM PMSF, pH 7.0). Protein extracts were centrifuged, filtered, and then subject to multiple enrichment steps for PBP2a_mecA_. Protein fractionation was performed on an Akta Avant chromatography system (Cytiva, Marlborourgh MA). Stage strong cation exchange (SCX) fractionation was performed using a HiTrap SP HP column (SCX Buffer A: 50 mM HEPES pH 7.0, SCX Buffer B: 50 mM HEPES + 1 M NaCl pH 7.0, proteins loaded in an isocratic gradient of 100% SCX Buffer A at 2 mL/min, rinsed with 25% SCX Buffer B at 2.5 mL/min, and eluted with 75% SCX Buffer B at 2.5 mL/min) (Cytiva, Marlborourgh MA). The SCX protein fraction was dialyzed overnight at 10 °C (Dialysis Buffer: 20 mM Tris, 100 mM NaCl, pH 7.5) using 20 kDa MWCO Slide-a-Lyzer dialysis cassettes (Thermo Fisher Scientific, Waltham, MA).

### PBP2a immunoaffinity enrichment and detection

PBP2a_mecA_ affinity enrichment was performed offline following gentle lysis using a mouse anti-MRSA antibody from RayBiotech (Palmtree Corners, GA) coupled to Pierce Protein G magnetic beads (Thermo Fisher Scientific, Waltham MA). Briefly, dialyzed SCX fractions were incubated with antibody-coupled beads over night at 10 °C under constant rotation. Magnetic beads were washed and immunocaptured proteins were released using 100 mM glycine, pH 2.0. Immunopurified PBP2a extracts were separated by SDS-PAGE for gel band peptide analysis or protein gel staining.

### Immunochromatographic PBP2amecA detection

Detection of PBP2a_mecA_ by an immunochromatographic membrane assay was performed with the Alere PBP2a SA Culture Colony Test kit per the manufacturer’s instructions (Abbott, Abbott Park, IL).

### Protein gel electrophoresis, staining, and in-gel digestion

Immunopurified PBP2a MRSA protein fractions were separated on 10% Tris–Glycine SDS-PAGE gels (Thermo Fisher Scientific, MA). Silver staining and destain of separated proteins was performed per manufactures instructions using the Pierce silver stain kit for mass spectrometry (Thermo Fisher Scientific, Waltham MA). In-gel peptide analysis of PBP2a_mecA_ was performed from excised gel bands. Briefly, gel bands were destained per the manufacturer’s protocol, washed in 100 mM NH_4_HCO_3_ (pH 8.0) and then acetonitrile, dried, and incubated with 50 ng of MS-grade Pierce trypsin protease or Proteinase K overnight (Thermo Fisher Scientific, Waltham MA). Peptides were extracted from gel pieces by sequential addition of NH_4_HCO_3_ (pH 8.0) and acetonitrile. Collected supernatant was dried and analyzed by bottom-up peptide analysis. Glycoprotein detection of SDS-PAGE separated proteins was performed using the Pro-Q Emerald glycoprotein stain while total protein was stained using SYPRO Ruby (Thermo Fisher Scientific, Waltham MA). Staining and visualization was performed per manufacturer’s instructions.

### Liquid chromatography

Peptide samples were separated using a Vanquish Horizon UHPLC system coupled with a Hypersil GOLD C18 column (1.9 μm, 175 Å, 2.1 × 200 mm), maintained at 60 °C, and connected to an Ion Max API source (Thermo Fisher Scientific, Waltham MA). A binary gradient of solvent A (0.1% formic acid in water) and solvent B (10% water, 10% isopropanol, 0.1% formic acid in acetonitrile) was used, increasing from 5 to 40% solvent B over 60 min at a flow rate of 60 μL/min. Intact protein samples were separated using a Dionex Ultimate 3000 HPLC system (Thermo Fisher Scientific, Waltham MA) coupled with a ProSwift RP-4H column (1 × 250 mm), maintained at 60 °C, and connected to an Ion Max API source. A binary gradient of solvent A (0.2% formic acid in water) and solvent B (0.2% formic acid in acetonitrile) was used, increasing from 2 to 50% solvent B at a flow rate of 200 μL/min. Acquisition times for intact protein chromatography ranged from five minutes to two hours.

### Monolith solid phase extraction tip and modified nano-LC separation

Construction and fabrication of monolith reverse phase SPE tips was performed per previous publication and patent descriptions^49,58^. Equilibration, washing and sample loading of SPE tips was performed by centrifugation for two minutes at 2000 g. Tips were washed with 50 μL of 0.2% formic acid in acetonitrile and then equilibrated with 50 μL of 0.2% formic acid in water. Next, 50 μL of diluted (1:5 in water) *S. aureus* formic acid/acetonitrile protein extracts were loaded onto SPE tips. Tips were washed a second time with 0.2% formic acid in water prior to loading onto a modified Easy-nLC liquid chromatography system adapted to move them from sample rack to the in-line LC flow path. Proteins were eluted off the SPE tip at a flow rate of 3uL/min using a binary gradient of solvent A (0.2% formic acid in 10% acetonitrile) and solvent B (0.2% formic acid in acetonitrile).

### Mass spectrometry: peptide analysis

Peptide data acquisition was collected in data-dependent mode on a Q Exactive HF mass spectrometer (Thermo Fisher Scientific, Waltham MA). ESI source settings for peptide acquisition included: source voltage of 3.5 kV, heated capillary temperature of 250 °C, and S-lens RF set to 50 percent. Data acquisition was performed over a mass-to-charge (*m*/*z*) range of 300–2000 *m*/*z* during peptide elution, with data collected at 120,000 resolving power (at *m*/*z* 400) and 3 μscans/spectrum, while fragment spectra were collected at 30,000 resolving power (at *m*/*z* 400) and 3 μscans/spectrum. Peptides were isolated with a width of 2 *m*/*z* and fragmented using a normalized collision energy of 30 eV.

### Mass spectrometry: intact protein analysis

Top-down protein data acquisition was performed on a Q Exactive HF (Thermo Fisher Scientific, Waltham MA) system using protein mode. ESI source settings for protein ionization included: source voltage of 3.5 kV, heated capillary temperature of 350 °C, S-lens voltage of 70, and recommended gas settings for the HPLC flow rate. Single stage MS data acquisition was performed over a range of 500–2000 *m*/*z* during protein elution, with data collected at 15,000 resolving power (at *m*/*z* 400), an AGC target value of 3 × 10^6^, 10 μscans/spectrum and maximum injection time of 250 ms. Fragmentation spectra were acquired with a 1.5–3 *m*/*z* isolation window, at a resolving power of 120,000 (at *m*/*z* 400), an AGC target value of 10^6^, with 5 μscans/spectrum, and the FTMS maximum injection time of 300 ms.

### Mass spectrometry: protein transfer charge reduction (PTCR) protein analysis

Data was collected on an Orbitrap Fusion Lumos™ modified to introduce anion reagent ions for PTCR experiments. Briefly, HPLC-separated proteins over 20 min were sequentially ionized and isolated, ion-ion reactions were performed with perfluoroperhydrophenanthrene (PFPP) reagent anions (MilliporeSigma, Burlington, MA), and detection of charge-reduced proteins was performed in the ion trap. PTCR spectra were acquired under the following instrument settings: 550–1500 *m*/*z* scan range, 10^5^ AGC target, 2 × 10^5^ PTCR reagent target, 10 μscans/spectrum, 1 ms PTCR reaction time, and 5 *m*/*z* precursor isolation windows centered every 5 *m*/*z* starting *m*/*z* 742 to 787. The ESI source voltage was 3.5 kV and heated capillary temperature was 275 °C.

### Mass spectrometry: source dissociation method for MRSA identification

In source fragmentation of PBP2a_mecA_ and PBP2a_mecC_ and the generation of N-terminal peptide fragments was performed on a Q Exactive HF mass spectrometer (Thermo Fisher Scientific, Waltham MA) using an in-source CID voltage of 40 or 50 eV. Selected ion monitoring (SIM) data was collected at 120,000 resolution, using an isolation width of 3.0 *m*/*z*, and 10 μscan. MS/MS data were collected at 30,000 resolution, using an isolation width of 2.5 *m*/*z*, 3 μscans, and with a fragmented with a collision energy of 25 eV. Direct infusion of PBP2a_mecA_ and PBP2a_mecC_ synthetic peptides was performed to verify in-source dissociated peptide masses and CID fragment product ion for wild-type proteins.

### Mass spectrometry: data analysis

Peptide spectra from bottom-up analyses were identified using Proteome Discoverer 2.5 (Thermo Fisher Scientific, Waltham MA) with Sequest HT and searched against a reviewed database of manually annotated *S. aureus* (NCTC 8325) proteins (2889 protein sequences, 797,376 residues) and a databased of common contaminants (179 protein sequences, 51,237 residues). A curated protein sequence for the PBP2a_mecA_ protein (UniProt 53707, 668 residues) from *S. aureus* was included in the above database. Peptide search parameters included full proteolytic cleavage (i.e., for trypsin or proteinase K), a maximum of two missed cleavages, a minimum peptide length of 6 amino acids, a precursor mass tolerance of ± 10 ppm, and a fragment mass tolerance of ± 0.02 Da. Determination of PBP2a_mecA_ molecular weight accomplished by manual inspection of intact protein spectra. Interpretation of intact protein MS/MS fragment spectra and fragment ion assignment was performed manually with the aid of the MS-Product utility from Protein Prospector (http://prospector.ucsf.edu/prospector) as well as the Prosight Lite software tool for top-down proteomics^59^. Average precursor mass and a ± 10 ppm fragmentation tolerance was used for Prosight Lite data processing. In source dissociated MS/MS spectra were manually annotated with the aid of the MS-Product utility program from Protein Prospector (http://prospector.ucsf.edu/prospector).

## Supplementary Information

Supplementary Information.

## Data Availability

Supporting raw data, mzXML, and protein sequence files can be found at the MassIVE proteomic data repository (https://massive.ucsd.edu/ProteoSAFe/static/massive.jsp).
