# Supplementary material for: Rapid MRSA detection via tandem mass spectrometry of the intact 80 kDa PBP2a resistance protein
Source: Sci Rep. 2021 Sep 15;11:18309. doi: 10.1038/s41598-021-97844-w (PMC8443585; doi:10.1038/s41598-021-97844-w)

Supplemental Information

Title

Rapid MRSA detection via tandem mass spectrometry of the intact 80kDa PBP2a resistance protein

Authors

Jason R Neil[1], Arvind Verma[2], Scott R. Kronewitter[1], William M. McGee[1], Christopher Mullen[3], Marjaana Viirtola[2], Annika Kotovuori[2], Herdis Friedrich[2], Johan Finell[2], Joni Rannisto[2], John E. P. Syka[3], James L. Stephenson Jr.[1*]

* Corresponding author

Corresponding author email address: jim.stephenson@thermofisher.com

Affiliations

[1]Thermo Fisher Scientific, Cambridge, MA, United States of America

[2]Thermo Fisher Scientific, Vantaa, Finland

[3]Thermo Fisher Scientific, San Jose, CA, United States of America

Supplemental Table S1.

Observed wild-type $PBP2a_{mecA}$ precursor ions during LC-separation and MS detection, associated charge states, and estimated intact protein masses (kDa).

Precursor (m/z)	Charge State	Estimated Mass
749.0506	108	80789.46
756.0164	107	80786.75
763.1251	106	80785.26
770.4351	105	80790.69
777.7800	104	80785.12
785.3582	103	80788.89
793.0142	102	80785.45
800.8558	101	80785.44
808.8633	100	80786.33
817.0276	99	80786.73
825.3572	98	80787.01
833.8500	97	80786.45
842.5315	96	80787.02
851.3875	95	80786.81
860.4332	94	80786.72
869.6699	93	80786.30
879.1185	92	80786.90
888.8340	91	80792.89
898.6044	90	80784.40
908.6761	89	80783.17
919.0220	88	80785.94
929.5832	87	80786.74
940.3804	86	80786.71
951.4436	85	80787.71
962.7549	84	80787.41
974.3535	83	80788.34
986.1979	82	80786.23
998.3879	81	80788.42

Supplemental Table S2.

Observed recombinant His$_6$-PBP2a$_{mecA}$ precursor ions during static infusion and MS detection, associated charge states, and estimated intact protein mass (kDa).

Precursor (m/z)	Charge State	Estimated Mass
689.4417	110	75728.59
695.7183	109	75724.29
702.0076	108	75708.82
708.4284	107	75694.84
714.9862	106	75682.54
721.6894	105	75672.39
728.4697	104	75656.85
735.4249	103	75645.76
742.4749	102	75630.44
749.6928	101	75617.97
757.0309	100	75603.09
764.5173	99	75588.21
772.1490	98	75572.60
779.9485	97	75558.00
787.8883	96	75541.28
796.0026	95	75525.25
804.2679	94	75507.18
812.7720	93	75494.80
821.3775	92	75474.73
830.2002	91	75457.22
839.2721	90	75444.49
848.0000	89	75383.00
857.8789	88	75405.34
867.4464	87	75380.84
877.3149	86	75363.08
887.4195	85	75345.66
897.7188	84	75324.38
908.2631	83	75302.84
919.0792	82	75282.49
930.1249	81	75259.12
941.4857	80	75238.86
953.0951	79	75215.51
965.0383	78	75194.99
977.0000	77	75152.00
989.7187	76	75142.62

Supplemental Table S3**.**

Observed wild-type PBP2a$_{mecA}$ precursor ions during chromatographic separation and charge-reduced using PTCR and MS detection approach, associated charge states, and estimated intact protein masses (kDa).

**Precursor (m/z)**	**Charge State**	**Estimated Mass**
851.40	95	80788.00
860.50	94	80793.00
869.70	93	80789.10
879.10	92	80785.20
888.70	91	80780.70
898.50	90	80775.00
908.60	89	80776.40
919.00	88	80784.00
929.50	87	80779.50
940.20	86	80771.20
951.20	85	80767.00

Supplemental Table S4.

Observed wild-type PBP2a$_{mecA}$ MS/MS product ions for precursor *m/z* 793.0 (charge state = 102) using HCD dissociation on a Q Exactive HF mass spectrometer.

Location	Protein Fragment	Amino Acids	Charge State	m/z (observed)	m/z (theoretical)	Δm (ppm)
N-terminus	b_9	1-9	2+	540.3544	540.3541	0.56
	b_{10}	1-10	2+	596.8964	596.8961	0.50
	b_{11}	1-11	3+	435.9615	435.9612	0.69
			2+	653.4386	653.4382	0.61
	b_{12}	1-12	3+	473.6561	473.6559	0.42
			2+	709.9804	709.9802	0.28
	b_{13}	1-13	3+	506.6790	506.6787	0.59
			2+	759.5153	759.5144	1.18
	b_{14}	1-14	3+	539.7018	539.7015	0.56
			2+	809.0488	809.0486	0.25
	b_{15}	1-15	3+	572.7245	572.7243	0.35
			2+	858.5835	858.5828	0.82
	b_{16}	1-16	3+	605.7474	605.7471	0.50
C-Terminus	y_{142}	527-668	16+	1011.0194	1011.0178	1.58
			15+	1078.3536	1078.3519	-1.67
			14+	1155.3075	1155.3051	2.08
	y_{139}	530-668	18+	881.5087	881.5074	1.47
			17+	933.3032	933.3016	1.71
			16+	991.5708	991.5699	0.91
			15+	1057.6087	1057.6075	1.13
			14+	1133.0804	1133.0789	1.32
	y_{136}	533-668	16+	969.4337	969.4308	2.99
			15+	1033.9942	1033.9923	1.84
			14+	1107.7779	1107.7770	0.81
	y_{134}	534-668	17+	900.6949	900.6931	2.00
			16+	956.9248	956.9235	1.36
			15+	1020.6525	1020.6513	1.18
			14+	1093.4859	1093.4830	2.65
	y_{131}	538-668	16+	936.8547	936.8527	2.13
			15+	999.2422	999.2424	-0.20
			14+	1070.5486	1070.5449	3.46
	y_{130}	539-668	17+	875.1519	875.1510	1.03
			16+	929.7858	929.7850	0.86
			15+	-	991.7035	-
			14+	1062.4694	1062.4675	1.79
	y_{129}	540-668	17+	867.5609	867.5602	0.81
			16+	921.7205	921.7198	0.76
			15+	983.1018	983.1006	1.22
			14+	1053.2520	1053.2502	1.71
	y_{127}	542-668	16+	907.4661	907.4644	1.87
			15+	967.8971	967.8949	2.27
			14+	1036.9611	1036.9583	2.70

Note: y_{130}^{15+} was not observed as isotopic pattern overlaps with y_{139}^{16+}

Supplemental Table S5.

Observed PBP2a$_{mecA}$ peptides following cell lysate fractionation or in-gel digestion of immunopurified PBP2a from ATCC MRSA isolate BAA-44, associated charge states, observed and theoretical m/z values, mass error (ppm), and enzymes used.

Location	Sequence	Charge State	m/z (obs.)	m/z (theor.)	Error (ppm)	Enzyme
2-26	KIKIVPLILIVVVVGFGIYFYASK	3	893.8949	893.8920	3.2	Tryp
27-40	DKEINNTIDAIEDK	2	809.4052	809.4019	4.1	Tryp
34-40	IDAIEDK	2	402.2109	402.2109	0	Prot K
34-44	IDAIEDKNFKQ	3	440.8985	440.8980	1.1	Prot K
48-54	DSSYISK	2	400.1960	400.1953	1.7	Tryp
55-68	SDNGEVEMTERPIK	2	802.8857	802.8829	3.5	Tryp
69-76	IYNSLGVK	2	447.2586	447.2582	0.9	Tryp
77-83	DINIQDR	2	437.2257	437.2249	1.8	Tryp
94-100	RVDAQYK	2	440.2382	440.2378	1.1	Tryp
101-110	IKTNYGNIDR	3	398.5480	398.5473	1.8	Tryp
103-110	TNYGNIDR	2	476.7287	476.7278	1.8	Tryp
111-118	NVQFNFVK	2	498.2711	498.2691	4.0	Tryp
125-138	LDWDHSVIIPGMQK	3	546.9510	546.9485	4.6	Tryp
139-148	DQSIHIENLK	3	399.5486	399.5472	3.5	Tryp
219-229	KMDEYLSDFAK	3	449.5482	449.5481	0.2	Tryp
230-241	KFHLTTNETESR	3	488.2474	488.2477	-0.6	Tryp
231-241	FHLTTNETESR	3	445.5504	445.5494	2.2	Tryp
248-265	ATSHLLGYVGPINSEELK	3	643.3433	643.3423	1.6	Tryp
271-280	GYKDDAVIGK	2	533.2842	533.2824	3.4	Tryp
290-298	KLQHEDGYR	3	382.5280	382.5281	-0.3	Tryp
293-300	HEDGYRVT	2	488.7281	488.7278	0.6	Prot K
299-316	VTIVDDNSNTIAHTLIEK	3	661.6860	661.6846	2.1	Tryp
301-311	IVDDNSNTIAH	2	599.7888	599.7886	0.3	Prot K
320-331	DGKDIQLTIDAK	3	439.5739	439.5734	1.1	Tryp
323-331	DIQLTIDAK	2	508.7851	508.7848	0.6	Tryp
342-382	NDYGSGTAIHPQTGELLALVSTPSYDVYPFMYGMSNEEYNK	3	1521.3666	1521.3613	3.5	Tryp
376-383	SNEEYNKL	2	498.7361	498.7353	1.6	Prot K
383-394	LTEDKKEPLLNK	3	476.6098	476.6083	3.2	Tryp
395-406	FQITTSPGSTQK	2	647.8361	647.8355	0.9	Tryp
407-417	ILTAMIGLNNK	2	594.3447	594.3445	0.3	Tryp
407-417	ILTAmIGLNNK	2	602.3432	602.3419	2.2	Tryp
418-426	TLDDKTSYK	3	357.5179	357.5170	2.5	Tryp
435-445	DKSWGGYNVTR	3	428.2113	428.2107	1.4	Tryp
446-456	YEVVNGNIDLK	2	632.3328	632.3326	0.3	Tryp
457-469	QAIESSDNIFFAR	2	749.3720	749.3903	2.3	Tryp
470-477	VALELGSK	2	408.7458	408.7449	2.2	Tryp
485-506	KLGVGEDIPSDYPFYNAQISNK	3	819.0778	819.0762	1.9	Tryp
486-506	LGVGEDIPSDYPFYNAQISNK	2	776.2796	776.3779	2.2	Tryp
505-513	NKNLDNEIL	2	536.7859	536.7853	1.1	Prot K
507-551	NLDNEILLADSGYGQGEILINPVQILSIYSALENNGNINAPHLLK	3	1620.8566	1620.8521	2.8	Tryp
582-589	THKEDIYR	3	354.5178	354.5173	1.4	Tryp
582-591	THKEDIYRSY	3	404.1998	404.1999	-0.2	Prot K
583-591	HKEDIYRSY	3	437.8832	437.8824	1.8	Prot K
590-597	SYANLIGK	2	433.2442	433.2425	3.9	Tryp
598-604	SGTAELK	2	353.1923	353.1925	-0.6	Tryp
613-634	QIGWFISYDKDNPNmMMAINVK	3	877.7516	877.7517	-0.1	Tryp
618-628	ISYDKDNPNMM	2	664.2878	664.2865	2.0	Prot K
620-628	YDKDNPNMM	2	564.2294	564.2284	2.0	Prot K
621-628	DKDNPNMM	2	482.6972	482.6968	0.8	Prot K
640-647	GMASYNAK	2	421.1970	421.1973	-0.7	Tryp
652-662	VYDELYENGNK	2	672.3105	672.3093	1.8	Tryp
663-668	KYDIDE	2	391.6818	391.6820	-0.5	Tryp

Supplemental Table S6.

List of quality control and clinical MSSA and MRSA isolates, associated characteristics, and sources.

Isolate	SCC*mec*	Resistance Gene	PFGE Type	Source
12600	-	-	n/a	American Type Culture Collection, USA
25923	-	-	n/a	American Type Culture Collection, USA
29737	-	-	n/a	American Type Culture Collection, USA
BAA-44	Type I	*mecA*	Iberian	American Type Culture Collection, USA
BAA-41	Type II	*mecA*	USA 100	American Type Culture Collection, USA
33592	Type III	*mecA*	ST239	American Type Culture Collection, USA
BAA-1683	Type IV a	*mecA*	USA 400	American Type Culture Collection, USA
BAA-2094	Type V	*mecA*	WA-MRSA	American Type Culture Collection, USA
BAA-42	Type VI	*mecA*	USA 800	American Type Culture Collection, USA
BAA-2313	Type XI	*mecC*	CC130	American Type Culture Collection, USA
BAA-1761	Type II	*mecA*	USA 100	American Type Culture Collection, USA
BAA-1720	Type II	*mecA*	USA 200	American Type Culture Collection, USA
BAA-1717	Type IV a	*mecA*	USA 300	American Type Culture Collection, USA
BAA-1707	Type IV	*mecA*	USA 400	American Type Culture Collection, USA
BAA-1763	Type IV	*mecA*	USA 500	American Type Culture Collection, USA
BAA-1754	Type IV	*mecA*	USA 600	American Type Culture Collection, USA
BAA-1766	Type V	*mecA*	USA 700	American Type Culture Collection, USA
BAA-1768	Type IV	*mecA*	USA 800	American Type Culture Collection, USA
BAA-1747	Type IV	*mecA*	USA 1000	American Type Culture Collection, USA
BAA-1764	Type IV	*mecA*	USA 1100	American Type Culture Collection, USA
BAA-2312	Type XI	*mecC*	n/a	American Type Culture Collection, USA
BAA-42	Type IV	*mecA*	USA 800	American Type Culture Collection, USA
33591	Type III	*mecA*	n/a	American Type Culture Collection, USA
BAA-38	Type I	*mecA*	n/a	American Type Culture Collection, USA
BAA-1750	Type II	*mecA*	USA 200	American Type Culture Collection, USA
BAA-1689	Type IV	*mecA*	USA 500	American Type Culture Collection, USA
43300	Type II	*mecA*	n/a	American Type Culture Collection, USA
BAA-1708	Type II	*mecA*	n/a	American Type Culture Collection, USA
MSSA Clin. Isolate 1	-	-	n/a	St. George's University Hospital, London UK
MRSA Clin. Isolate 1	Type IV a	*mecA*	n/a	Univ. Hospital Ramón y Cajal, Madrid Spain
MRSA Clin. Isolate 2	n/a	*mecA*	n/a	Univ. Hospital Ramón y Cajal, Madrid Spain
MRSA Clin. Isolate 3	Type IV a	*mecA*	n/a	Univ. Hospital Ramón y Cajal, Madrid Spain
MRSA Clin. Isolate 4	n/a	*mecA*	n/a	Univ. Hospital Ramón y Cajal, Madrid Spain
MRSA Clin. Isolate 5	n/a	*mecA*	n/a	Univ. Hospital Ramón y Cajal, Madrid Spain
MRSA Clin. Isolate 6	Type IV a	*mecA*	n/a	Univ. Hospital Ramón y Cajal, Madrid Spain
MRSA Clin. Isolate 7	n/a	*mecA*	n/a	Univ. Hospital Ramón y Cajal, Madrid Spain
MRSA Clin. Isolate 8	n/a	*mecA*	n/a	Univ. Hospital Ramón y Cajal, Madrid Spain
MRSA Clin. Isolate 9	Type IV c	*mecA*	n/a	Univ. Hospital Ramón y Cajal, Madrid Spain
MRSA Clin. Isolate 10	Type IV c	*mecA*	n/a	Univ. Hospital Ramón y Cajal, Madrid Spain
MRSA Clin. Isolate 11	n/a	*mecA*	n/a	Univ. Hospital Ramón y Cajal, Madrid Spain
MRSA Clin. Isolate 12	Type IV a	*mecA*	n/a	Univ. Hospital Ramón y Cajal, Madrid Spain

n/a – not available

Supplemental Table S7.

N-terminal sequences from four different PBP2a variants, accession numbers, and species.

**PBP2a Variant**	**UniProt Accession Number**	**Species**	**N-terminal Sequence** (15 amino acids)
$PBP2a_{mecA}$	Q53707	*Staphylococcus aureus*	MKKIKIVPLILIVVV…
$PBP2a_{mecB}$	A0A4Y1NMT9	*Macrococcus canis*	MKNKALAILIICICL…
$PBP2a_{mecC}$	F4NA57	*Staphylococcus aureus*	MKKIYISVLVLLLIM…
$PBP2a_{mecD}$	A0A1S7BGS4	*Macrococcus caseolyticus*	MKNIKVKILIVCSLC…

Supplemental Figure S1.

Intact recombinant His_6-$PBP2a_{mecA}$ MS/MS spectrum acquired with static infusion, isolation of precursor ion at m/z = 742.5 , isolation window of 3 Th.

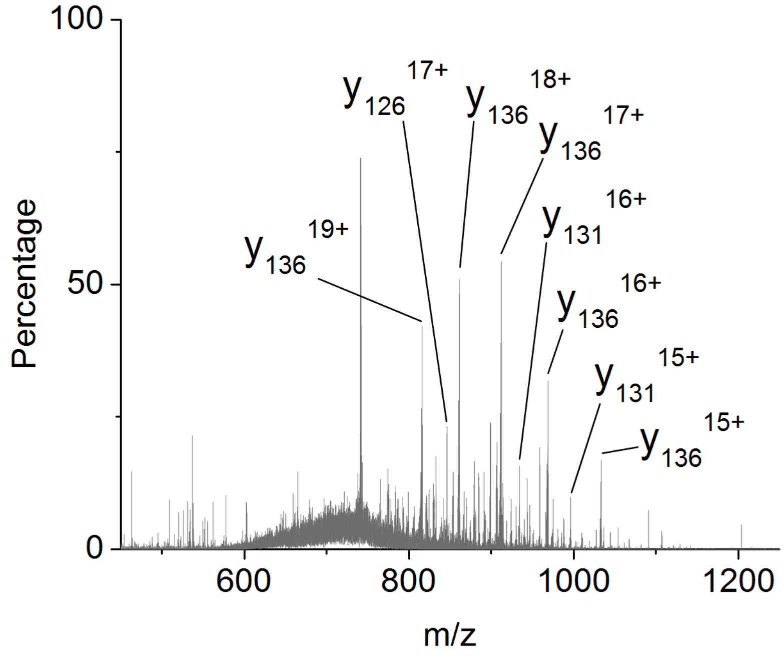

Supplemental Figure S2.

Detection of recombinant SDS-PAGE separated His$_6$-PBP2a$_{mecA}$ (1μg) and immunopurified PBP2a$_{mecA}$ from ATCC MRSA isolate BAA-44 with ProQ Emerald glycoprotein stain, and total protein using Sypro Ruby total protein stain or silver stain (left side). Detection of immunopurified protein components before and after treatment with deglycosylation mix (right side). Original gel images are displayed on the next page.

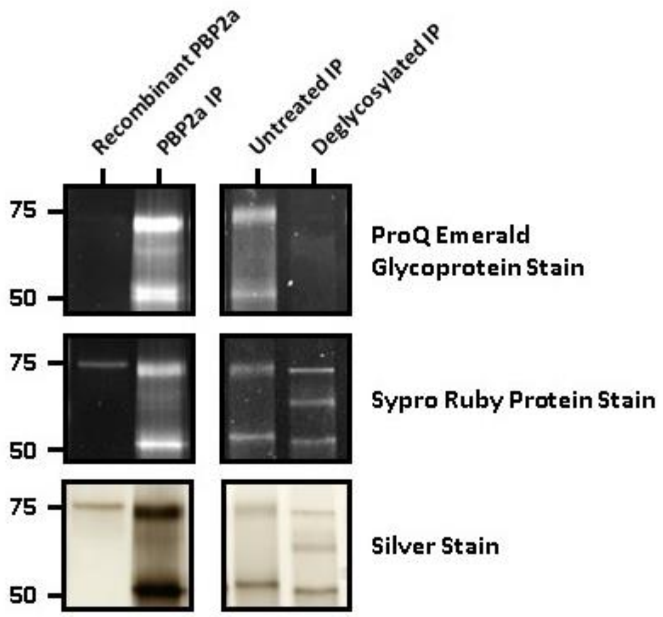

Supplemental Figure S2. cont.

Below are the original gel images for the previous figure. Red boxes highlight those areas of the gel that have been selected for display.

20160428_Pro-Q Emerald 300 Glycoprotein stain

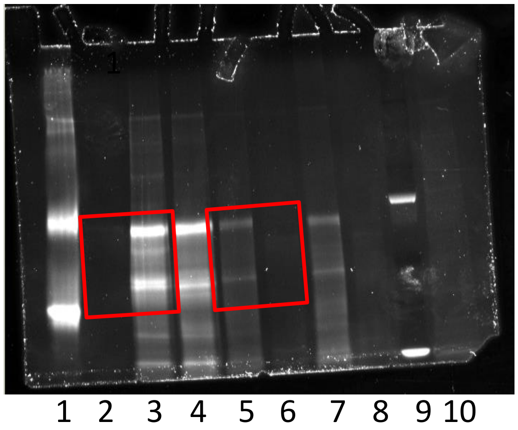

1. CandyCane std, 0.25µg of each protein
2. rPBP2a, 0.25µg
3. PBP2a IP 3.4.2016, 10µl
4. PBP2a IP 3.18.2016, 10µl
5. IP - untreated #2, 10µl
6. IP + treated #1, 10µl
7. IP – 3.22.2016, 10µl
8. IP + MIX 3.22.2016, 10µl
9. BioRad Precision plus std, 5µl
10. STA2; 25.11.2015, 10µl (Herdis)

(Items 3–8: from Jason)

20160429_Sypro Ruby Protein stain (stains all proteins)

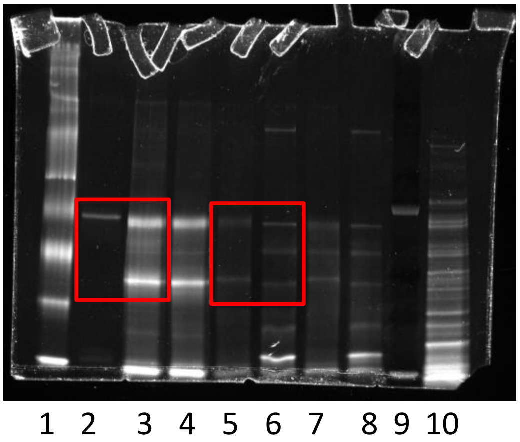

20160429_Silver stain

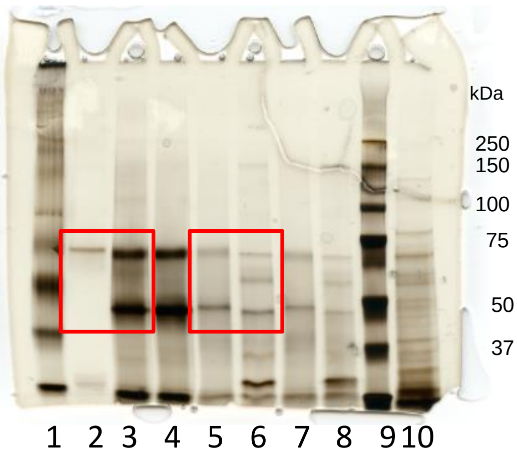

Supplemental Figure S3.1

KIKIVPLILIVVVVGFGIYFYASK

Location: aa 2-26

Precursor Charge State: 3

Theoretical Monoisotopic: 893.8920 *m/z*

Mass Error: 3.2 ppm

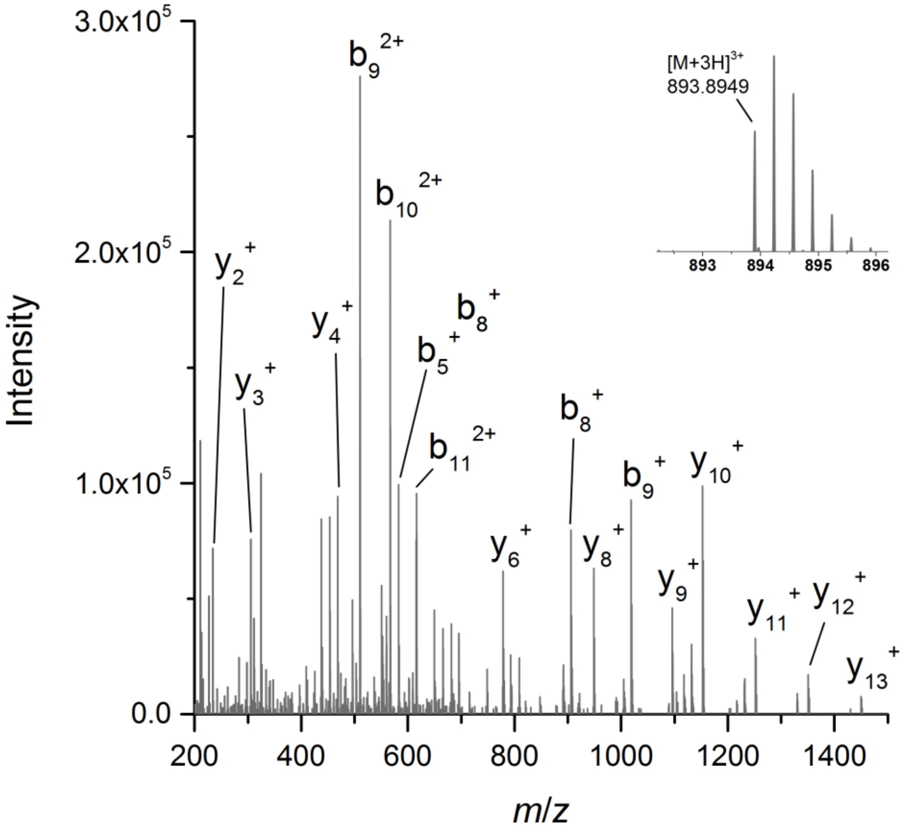

Supplemental Figure S3.2

DKEINNTIDAIEDK

Location: aa 27-40

Precursor Charge State: 2

Theoretical Monoisotopic: 809.4019 *m/z*

Mass Error: 4.1 ppm

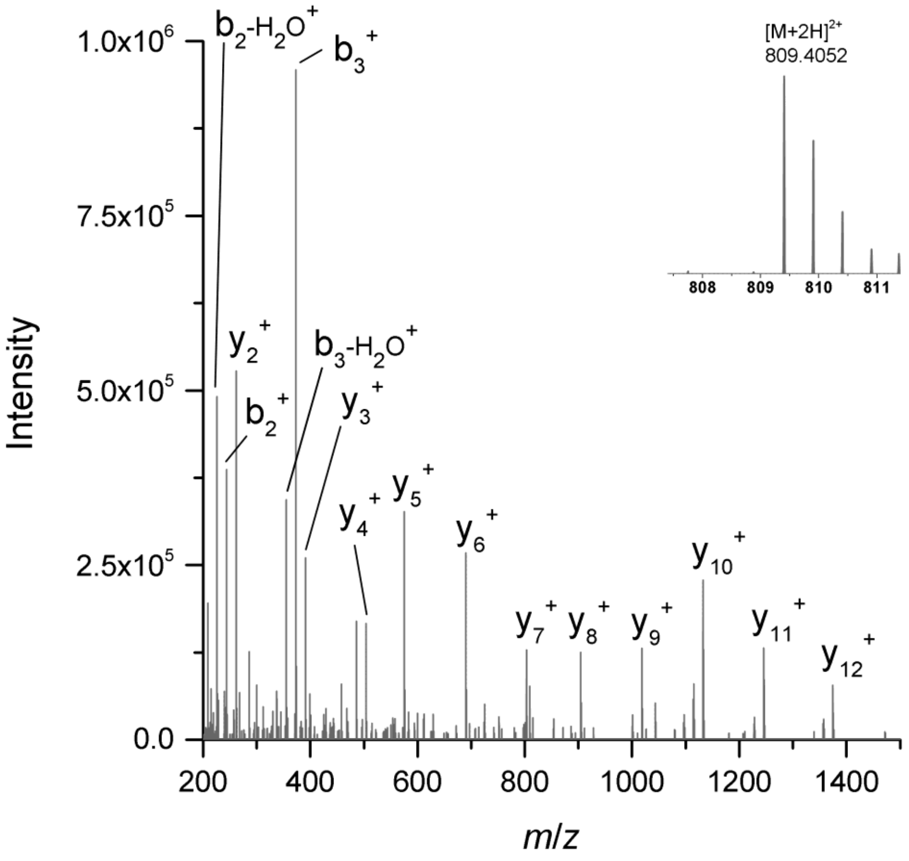

Supplemental Figure S3.3

IDAIEDK (Proteinase K)

Location: aa 34-40
Precursor Charge State: 2
Theoretical Monoisotopic: 402.2109 *m/z*
Mass Error: 0 ppm

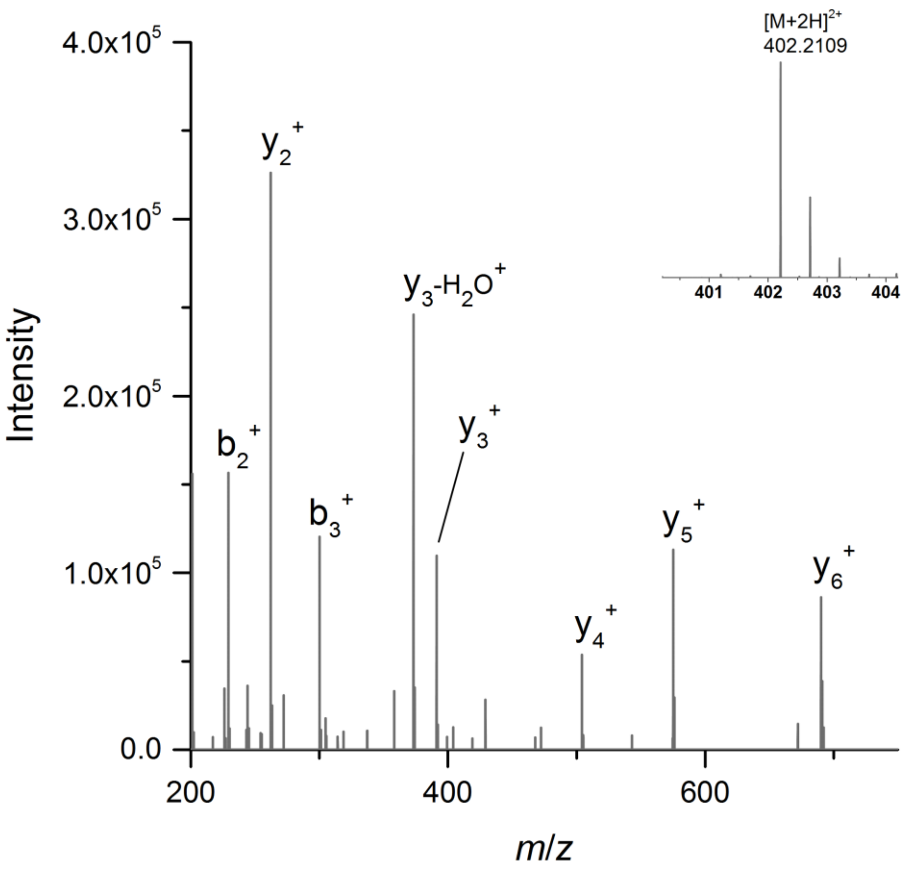

Supplemental Figure S3.4

IDAIEDKNFKQ (Proteinase K)

Location: aa 34-44
Precursor Charge State: 3
Theoretical Monoisotopic: 440.8980 *m/z*
Mass Error: 1.1 ppm

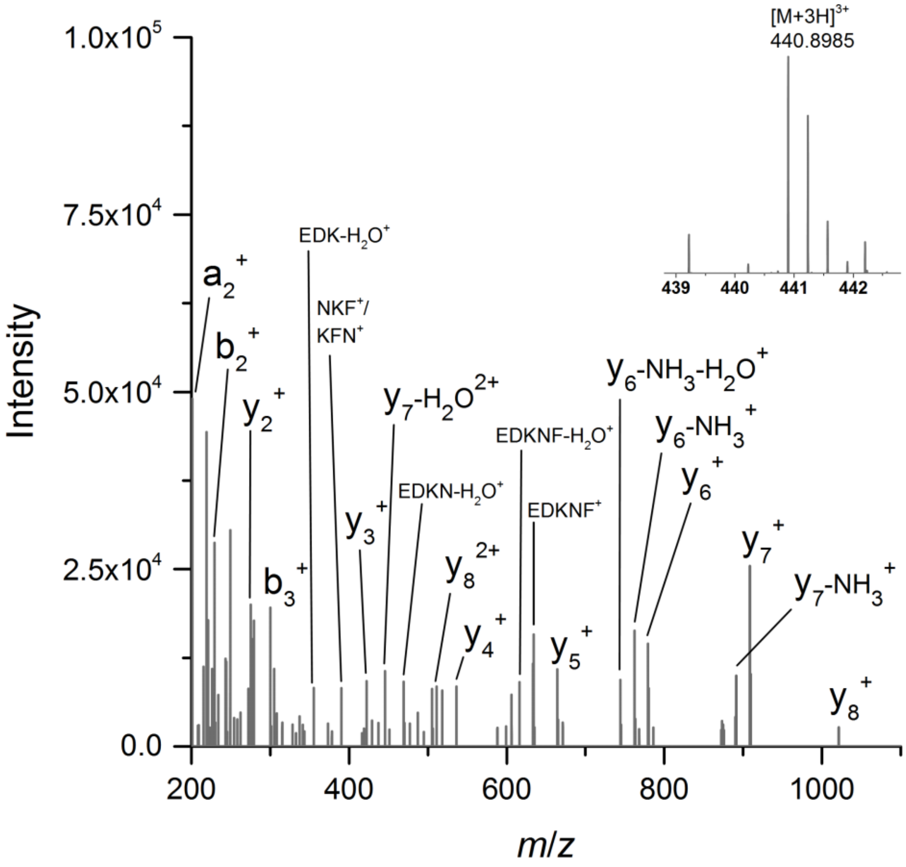

Supplemental Figure S3.5

DSSYISK

Location: aa 48-54
Precursor Charge State: 2
Theoretical Monoisotopic: 400.1953 *m/z*
Mass Error: 1.7 ppm

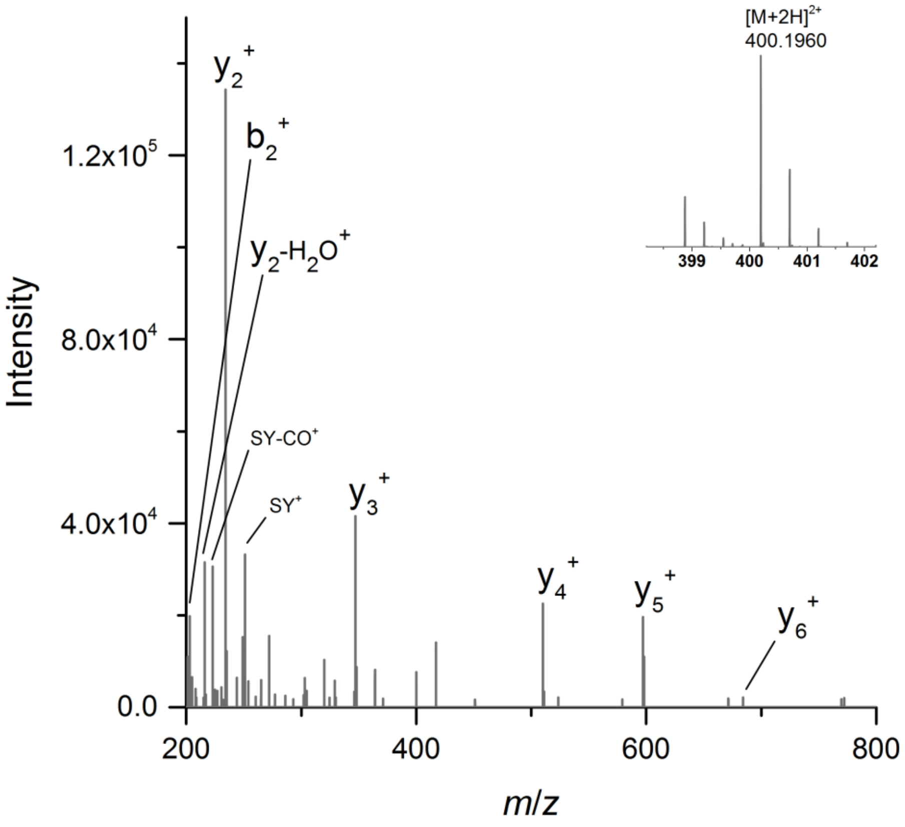

Supplemental Figure S3.6

SDNGEVEMTERPIK

Location: aa 55-68
Precursor Charge State: 2
Theoretical Monoisotopic: 802.8829 *m/z*
Mass Error: 3.5 ppm

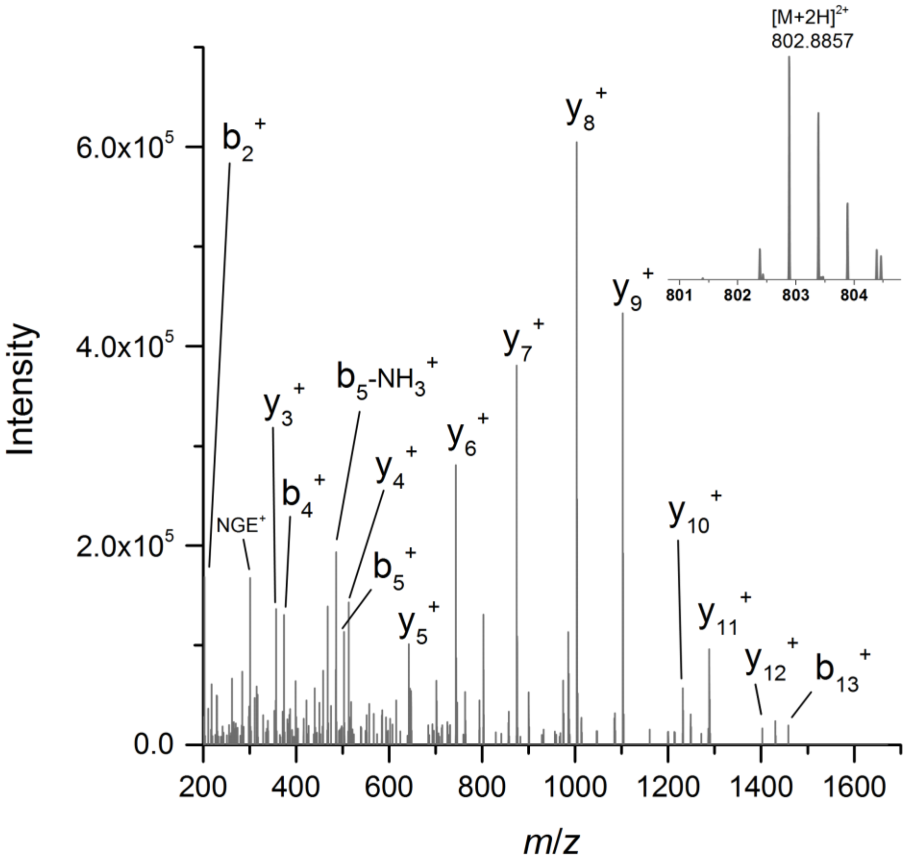

Supplemental Figure S3.7

IYNSLGVK

Location: aa 69-76
Precursor Charge State: 2
Theoretical Monoisotopic: 447.2582 *m/z*
Mass Error: 0.9 ppm

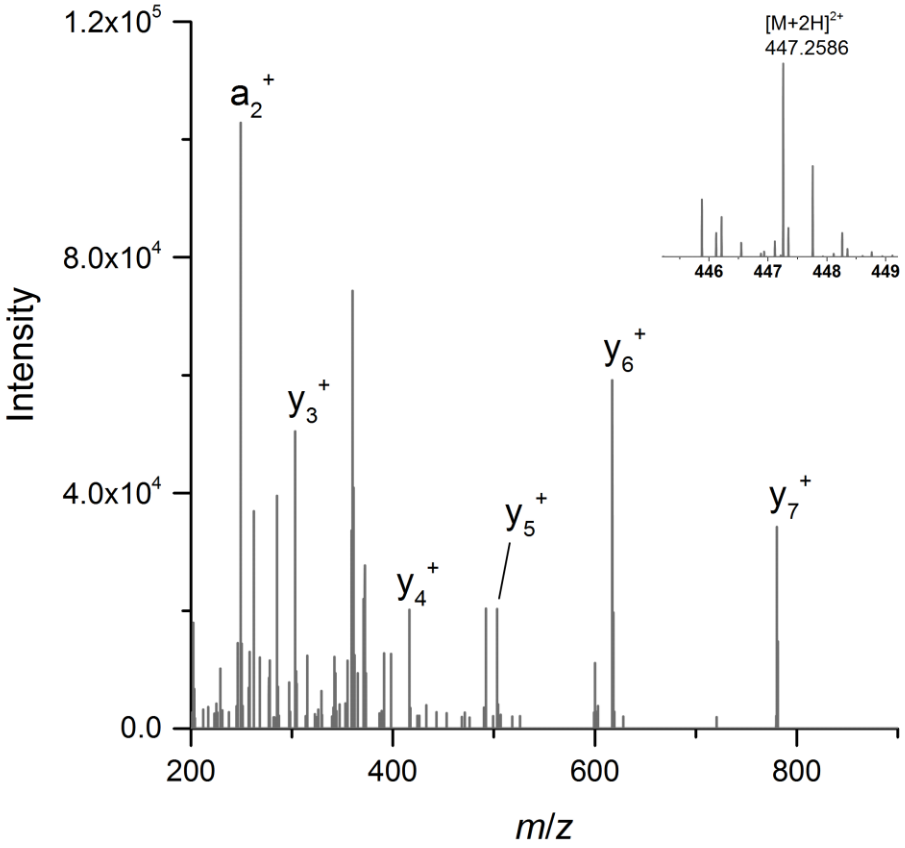

Supplemental Figure S3.8

DINIQDR

Location: aa 77-83
Precursor Charge State: 2
Theoretical Monoisotopic: 437.2249 *m/z*
Mass Error: 1.8 ppm

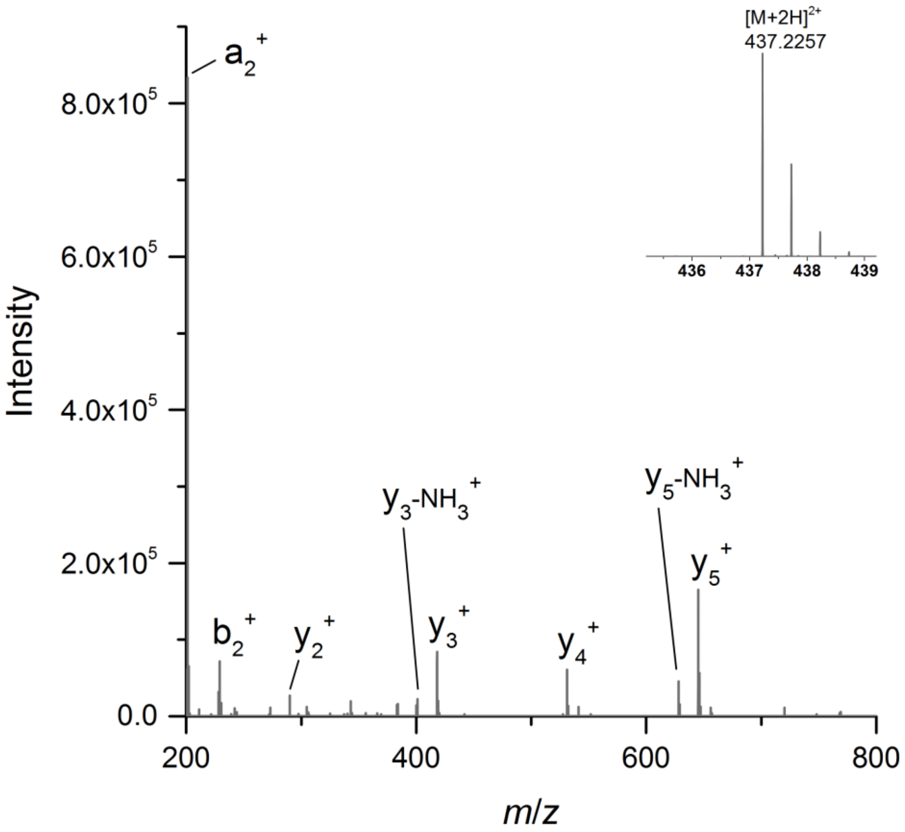

Supplemental Figure S3.9

RVDAQYK

Location: aa 94-100

Precursor Charge State: 2

Theoretical Monoisotopic: 440.2378 *m/z*

Mass Error: 1.1 ppm

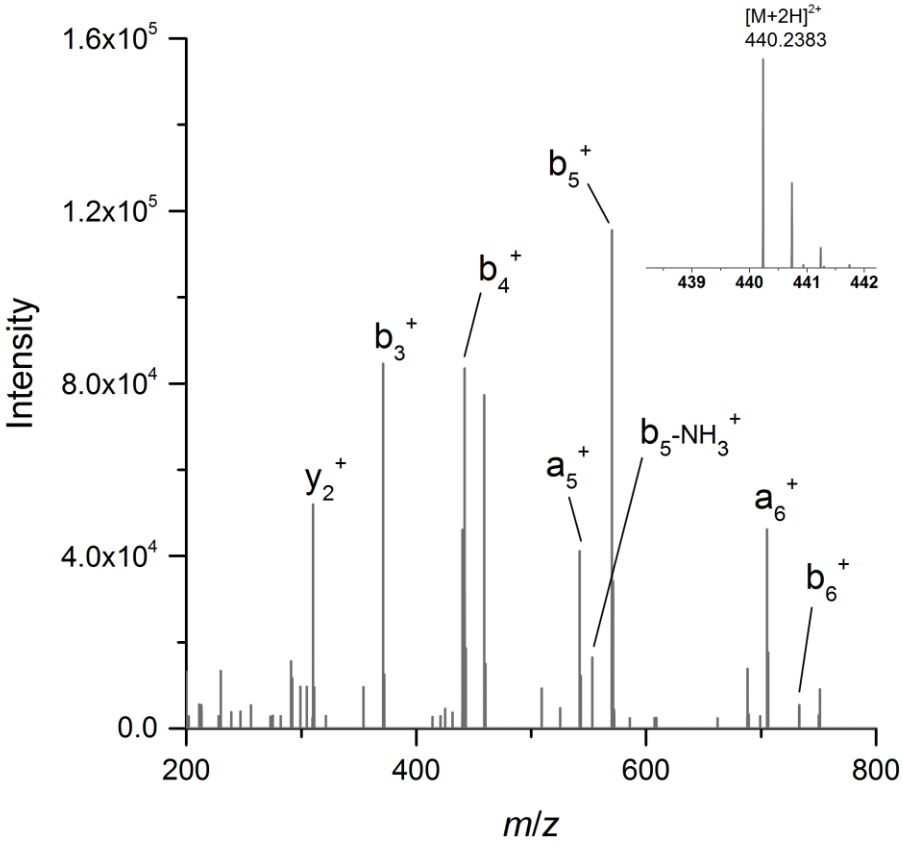

Supplemental Figure S3.10

IKTNYGNIDR

Location: aa 101-110

Precursor Charge State: 3

Theoretical Monoisotopic: 398.5473 *m/z*

Mass Error: 1.8 ppm

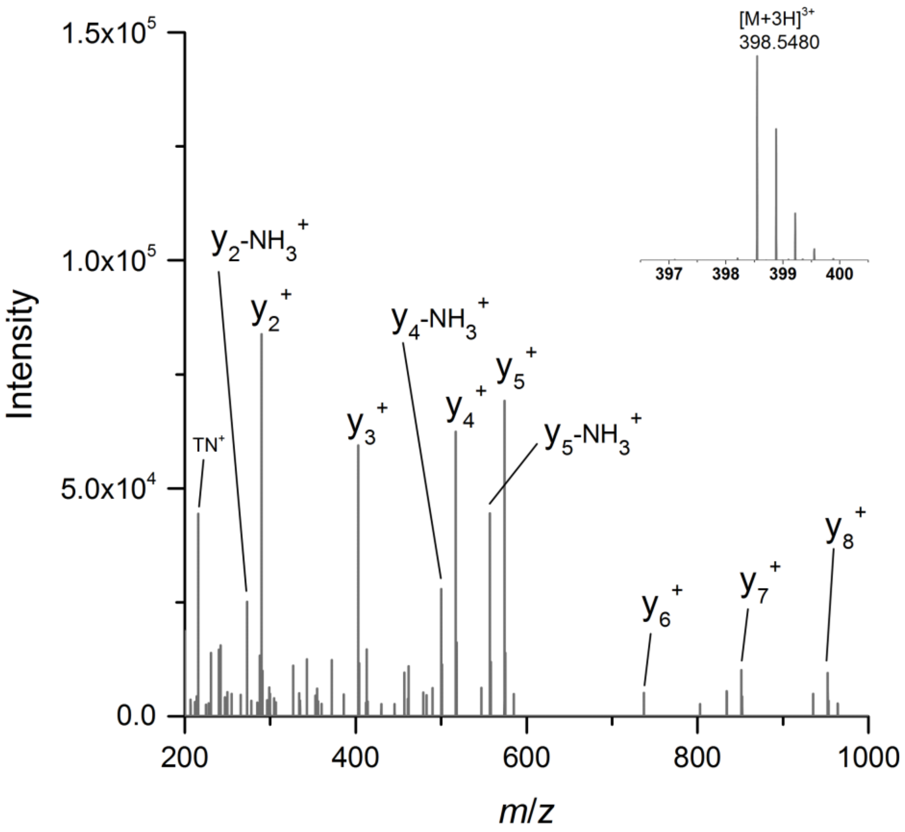

Supplemental Figure S3.11

TNYGNIDR

Location: aa 103-110
Precursor Charge State: 2
Theoretical Monoisotopic: 476.7278 *m/z*
Mass Error: 1.8 ppm

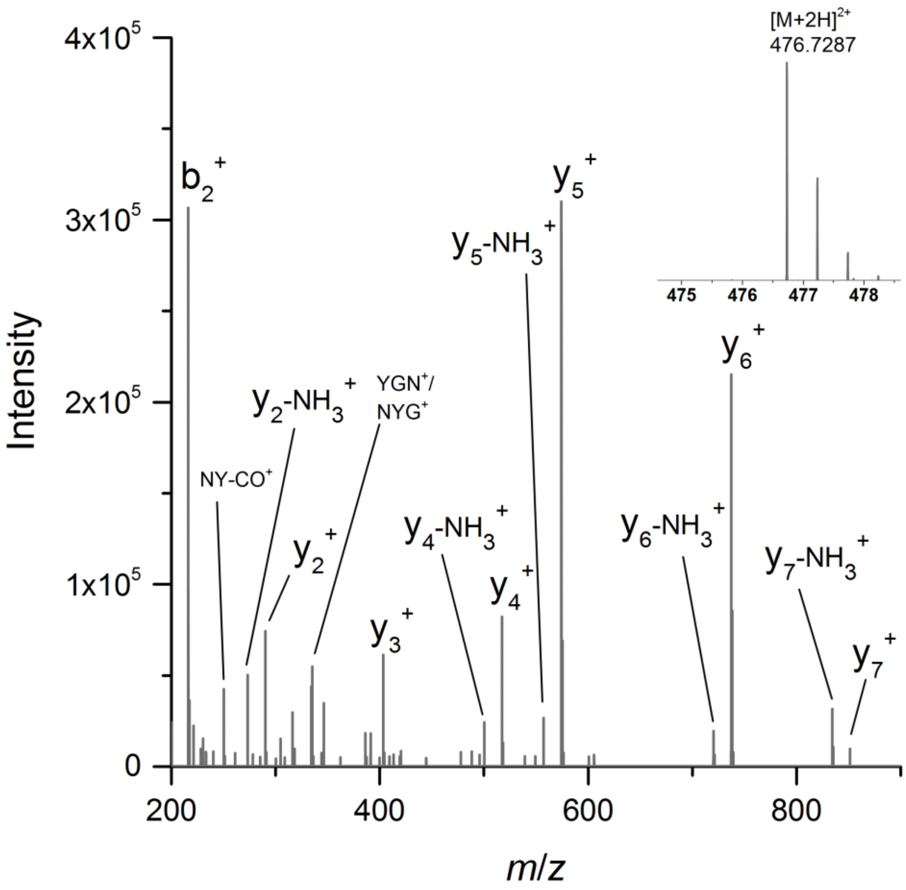

Supplemental Figure S3.12

NVQFNFVK

Location: aa 111-118
Precursor Charge State: 2
Theoretical Monoisotopic: 498.2691 *m*/*z*
Mass Error: 4.0 ppm

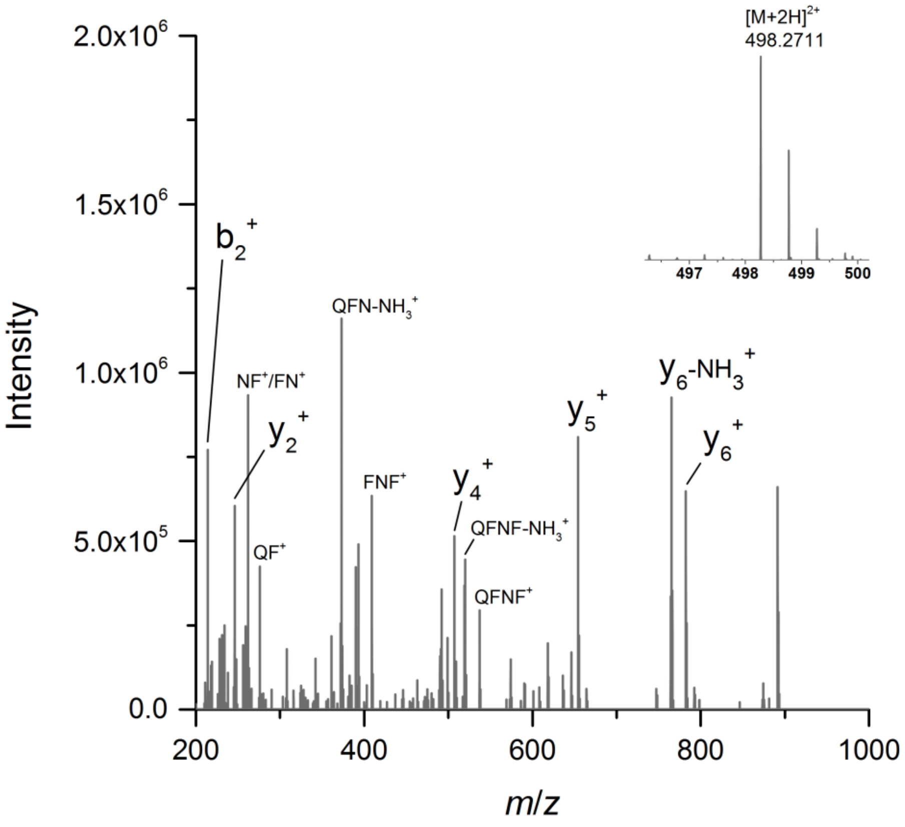

Supplemental Figure S3.13

LDWDHSVIIPGMQK

Location: aa 125-138

Precursor Charge State: 3

Theoretical Monoisotopic: 546.9485 *m/z*

Mass Error: 4.6 ppm

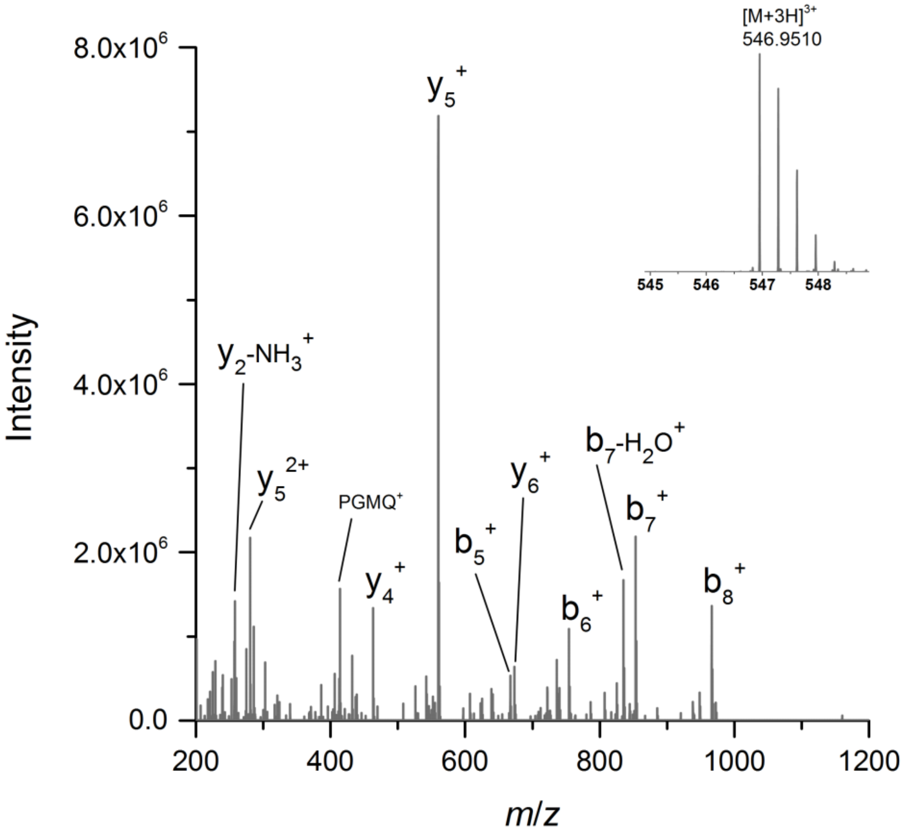

Supplemental Figure S3.14

DQSIHIENLK

Location: aa 139-148

Precursor Charge State: 3

Theoretical Monoisotopic: 399.5472 *m/z*

Mass Error: 3.5 ppm

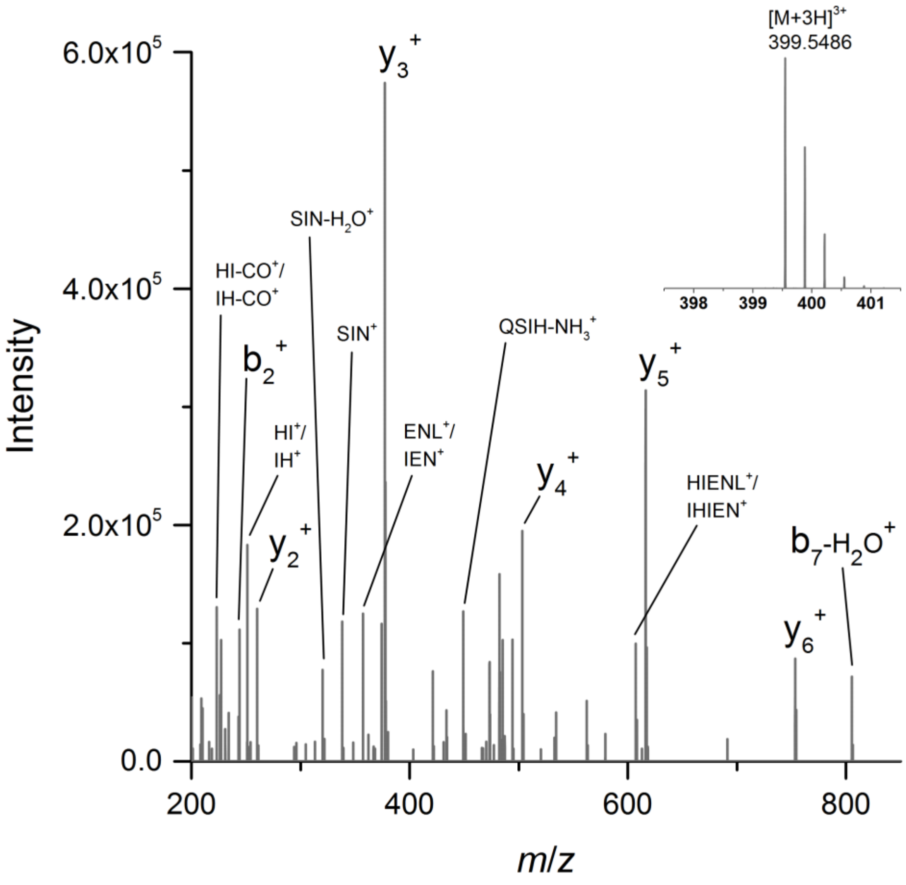

Supplemental Figure S3.15

KMDEYLSDFAK

Location: aa 219-229

Precursor Charge State: 3

Theoretical Monoisotopic: 449.5481 *m/z*

Mass Error: 0.2 ppm

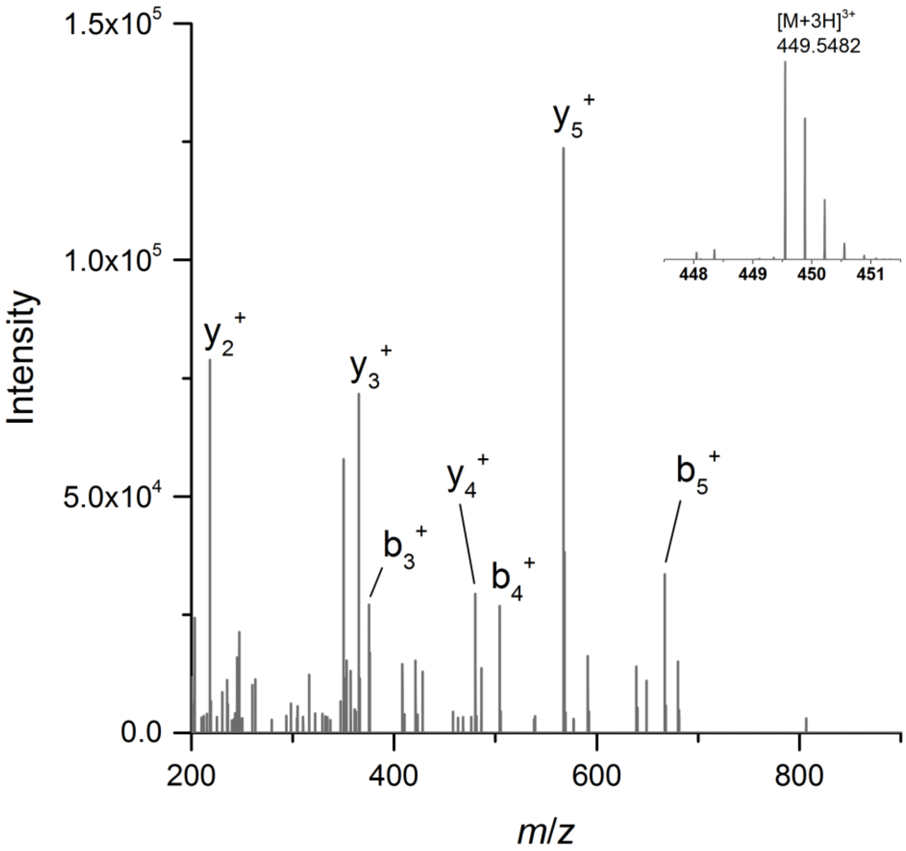

Supplemental Figure S3.16

KFHLTTNETESR

Location: aa 230-241

Precursor Charge State: 3

Theoretical Monoisotopic: 488.2477 *m/z*

Mass Error: -0.2 ppm

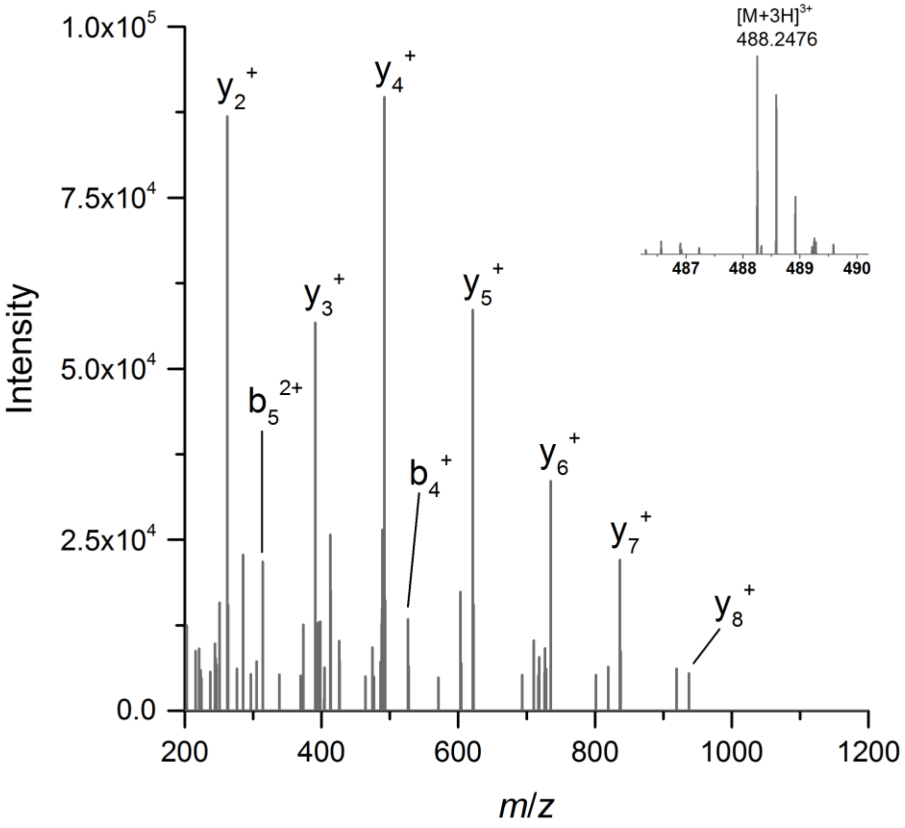

Supplemental Figure S3.17

FHLTTNETESR

Location: aa 231-241

Precursor Charge State: 3

Theoretical Monoisotopic: 445.5494 *m/z*

Mass Error: 2.2 ppm

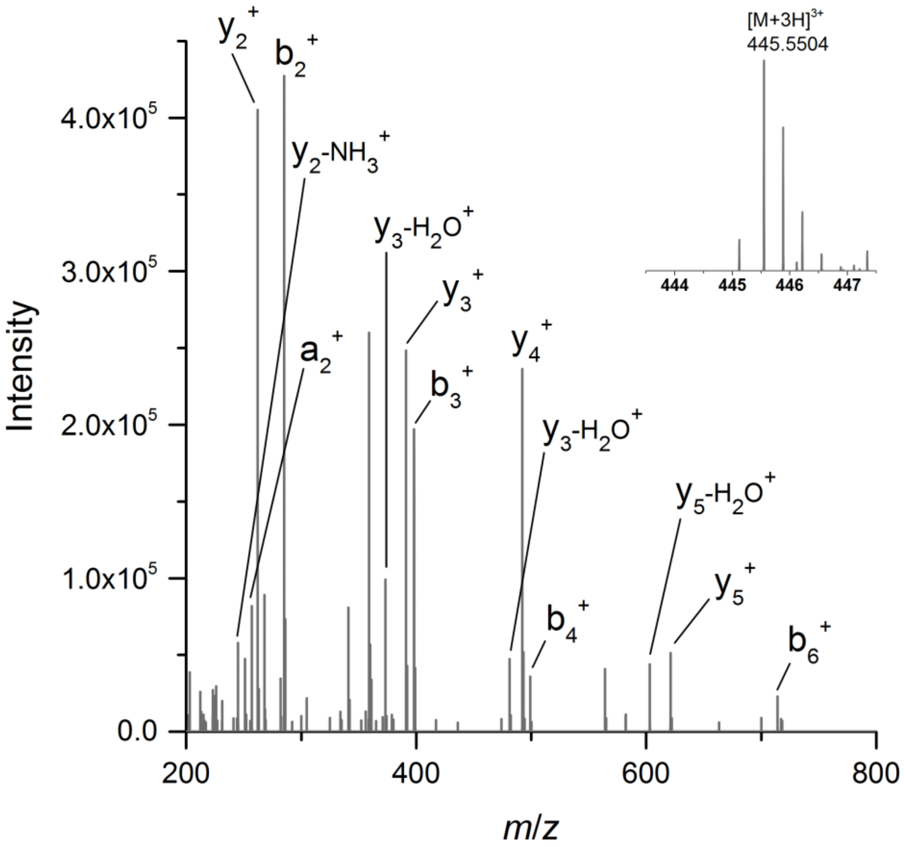

Supplemental Figure S3.18

ATSHLLGYVGPINSEELK

Location: aa 248-265

Precursor Charge State: 3

Theoretical Monoisotopic: 643.3423 *m/z*

Mass Error: 1.6 ppm

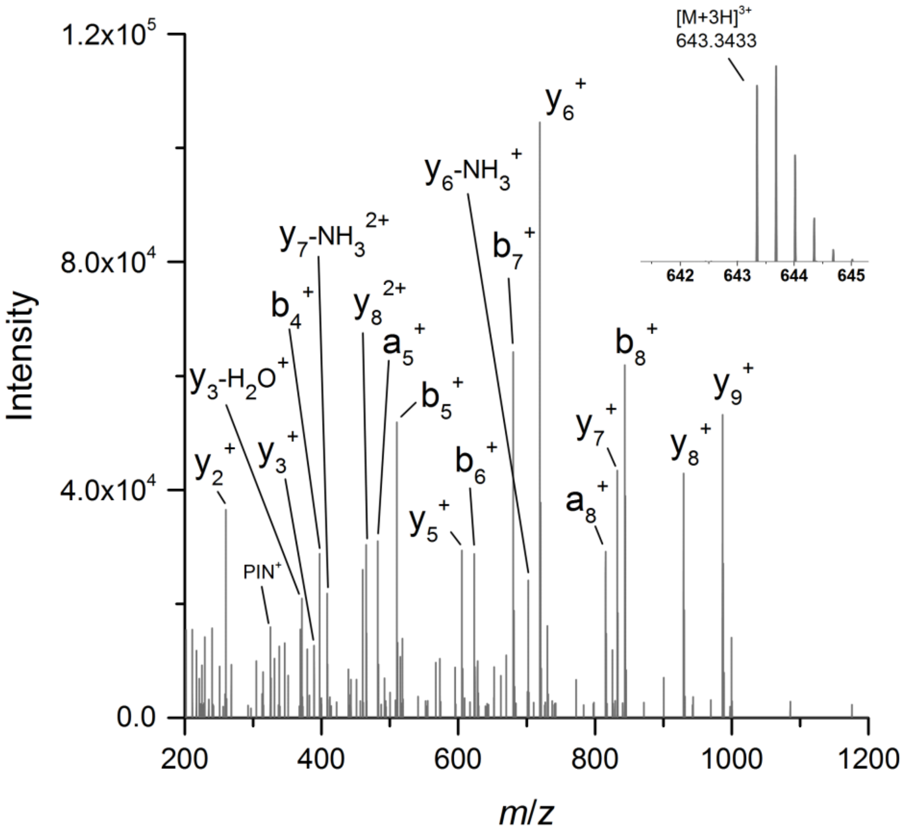

Supplemental Figure S3.19

GYKDDAVIGK

Location: aa 271-280

Precursor Charge State: 2

Theoretical Monoisotopic: 533.2824 *m/z*

Mass Error: 3.4 ppm

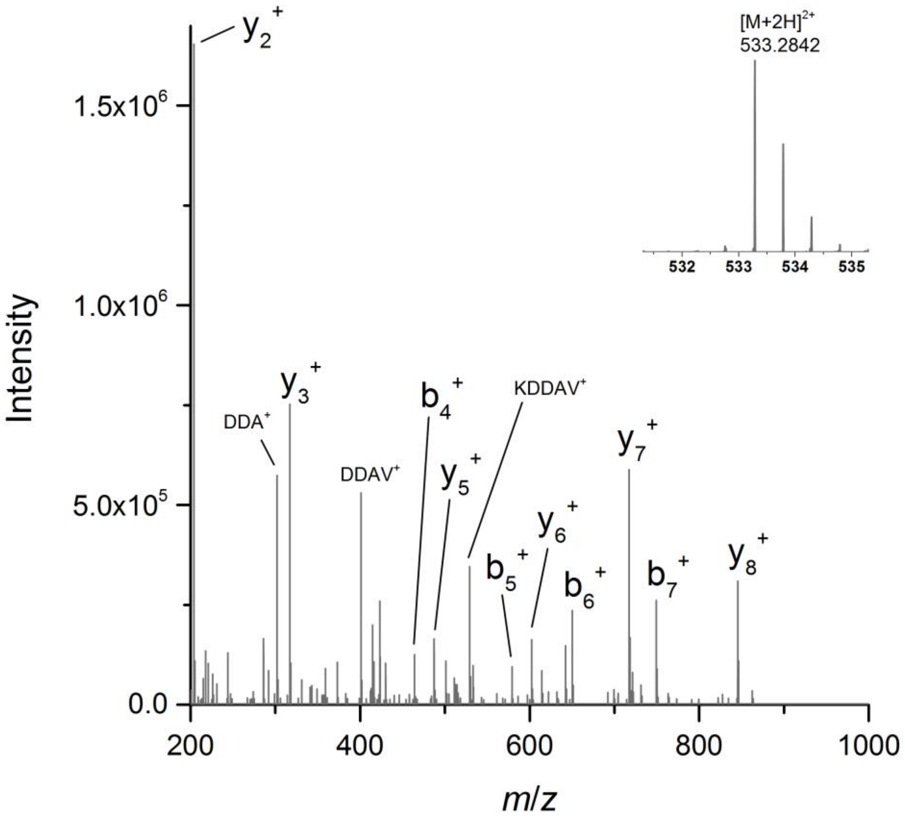

Supplemental Figure S3.20

KLQHEDGYR

Location: aa 290-298

Precursor Charge State: 3

Theoretical Monoisotopic: 382.5281 *m/z*

Mass Error: -0.3 ppm

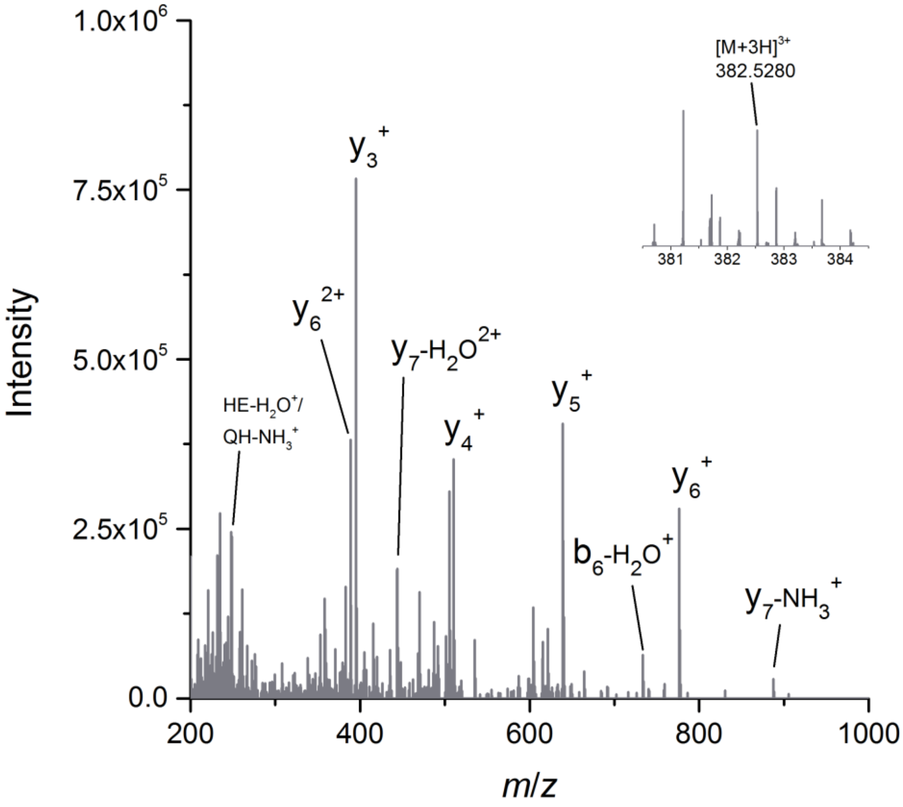

Supplemental Figure S3.21

HEDGYRVT (Proteinase K)

Location: aa 293-300
Precursor Charge State: 2
Theoretical Monoisotopic: 488.7278 *m/z*
Mass Error: 0.6 ppm

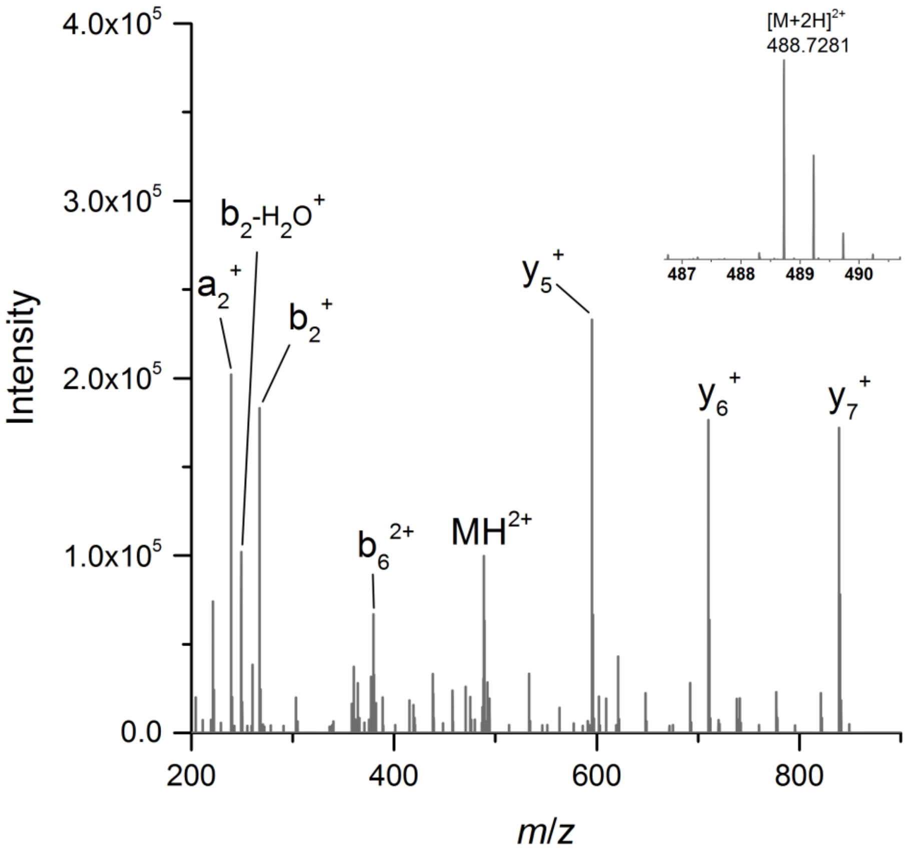

Supplemental Figure S3.22

VTIVDDNSNTIAHTLIEK

Location: aa 299-316

Precursor Charge State: 3

Theoretical Monoisotopic: 661.6846 *m/z*

Mass Error: 2.1 ppm

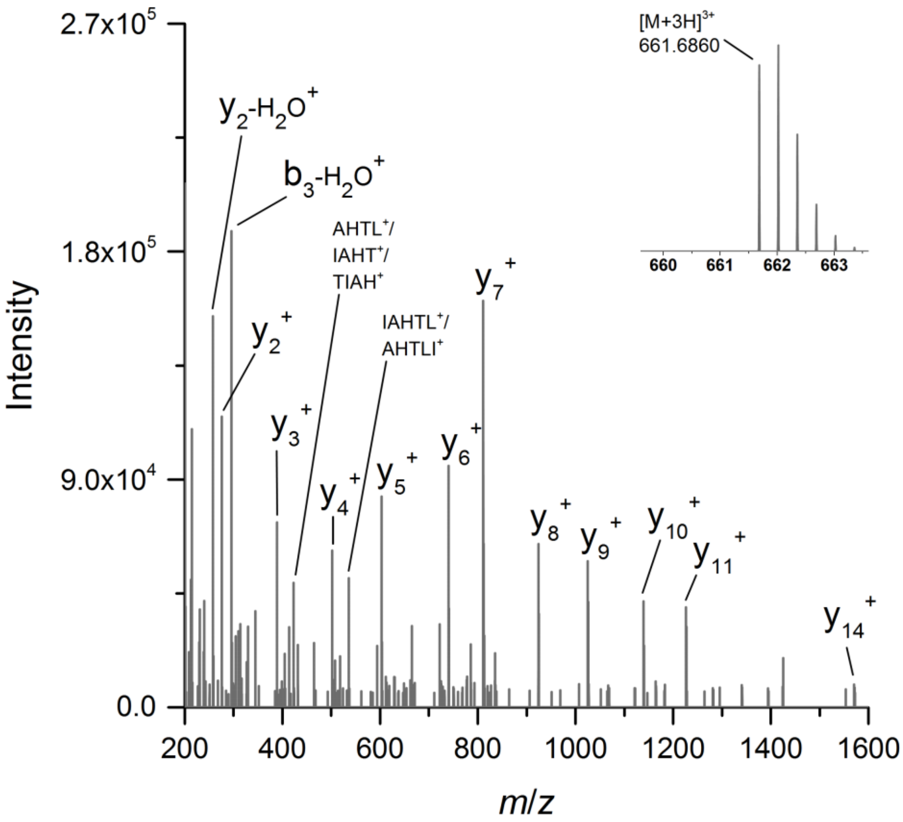

Supplemental Figure S3.23

IVDDNSNTIAH (Proteinase K)

Location: aa 301-311
Precursor Charge State: 2
Theoretical Monoisotopic: 599.7886 *m/z*
Mass Error: 0.3 ppm

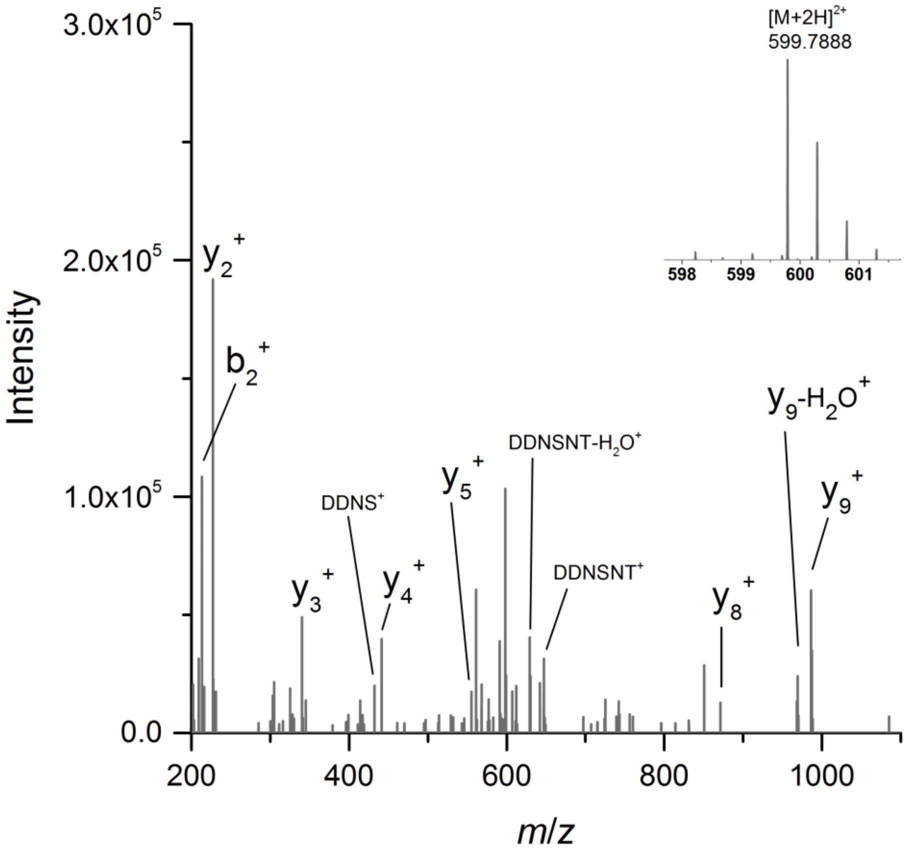

Supplemental Figure S3.24

DGKDIQLTIDAK

Location: aa 320-331
Precursor Charge State: 3
Theoretical Monoisotopic: 439.5734 *m/z*
Mass Error: 1.1 ppm

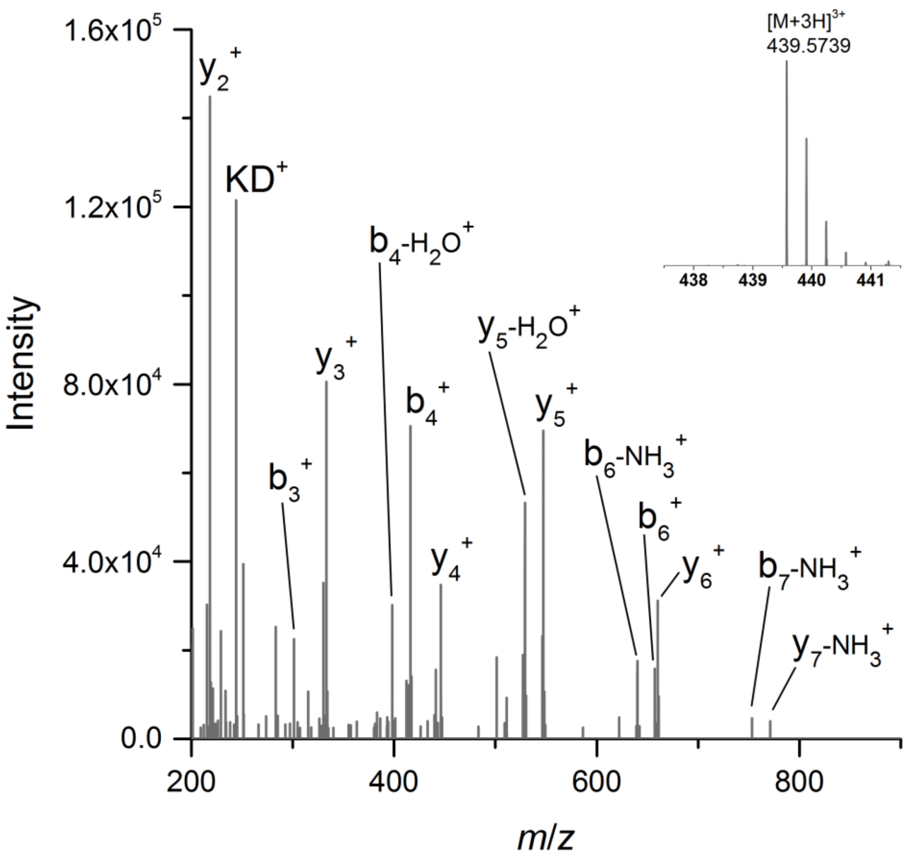

Supplemental Figure S3.25

DIQLTIDAK

Location: aa 323-331

Precursor Charge State: 2

Theoretical Monoisotopic: 508.7848 *m/z*

Mass Error: 0.6 ppm

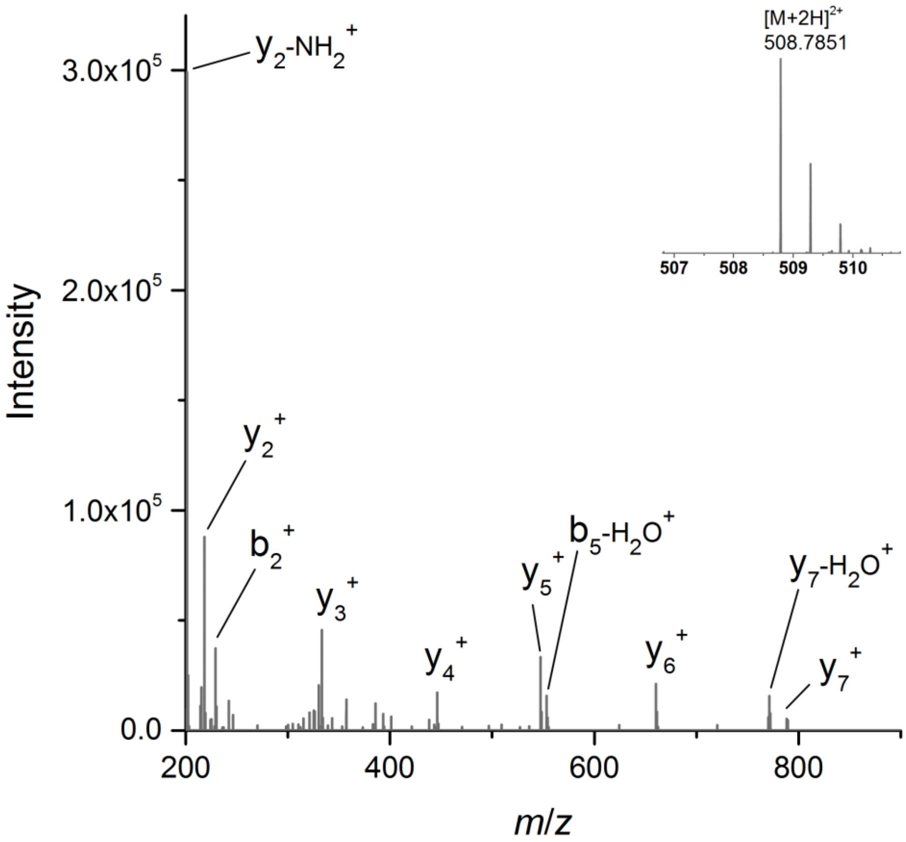

Supplemental Figure S3.26

NDYGSGTAIHPQTGELLALVSTPSYDVYPFMYGMSNEEYNK

Location: aa 342-382

Precursor Charge State: 3

Theoretical Monoisotopic: 1521.3613 *m/z*

Mass Error: 3.5 ppm

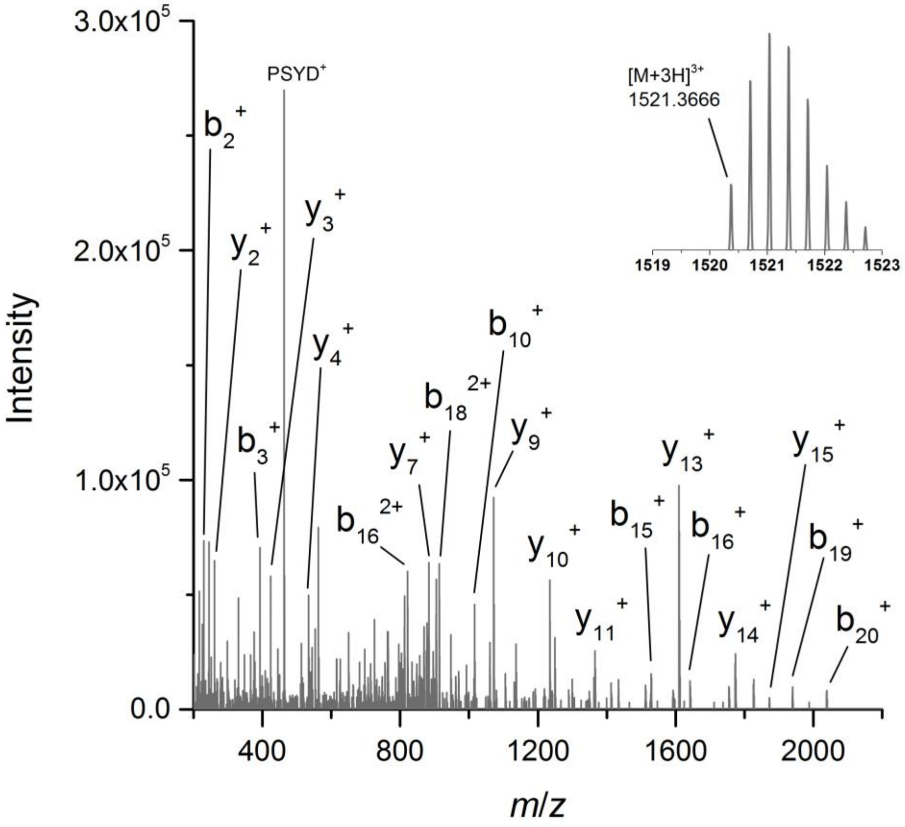

Supplemental Figure S3.27

SNEEYNKL (Proteinase K)

Location: aa 376-383

Precursor Charge State: 2

Theoretical Monoisotopic: 498.7353 *m/z*

Mass Error: 1.6 ppm

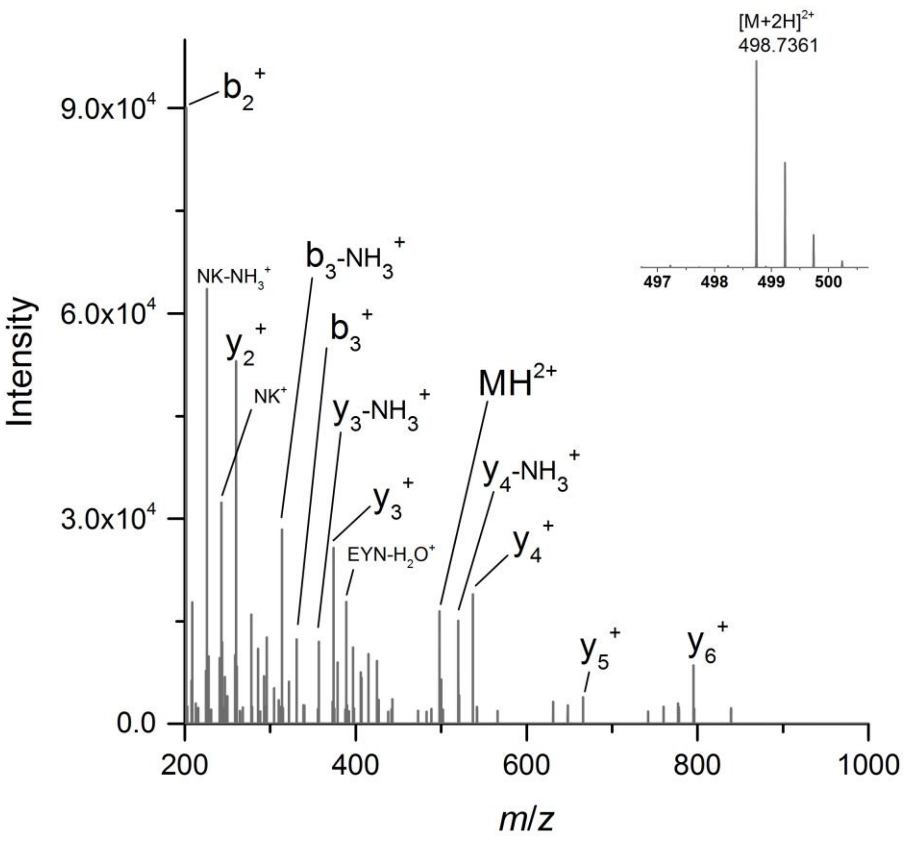

Supplemental Figure S3.28

LTEDKKEPLLNK

Location: aa 383-394

Precursor Charge State: 3

Theoretical Monoisotopic: 476.6083 *m/z*

Mass Error: 3.2 ppm

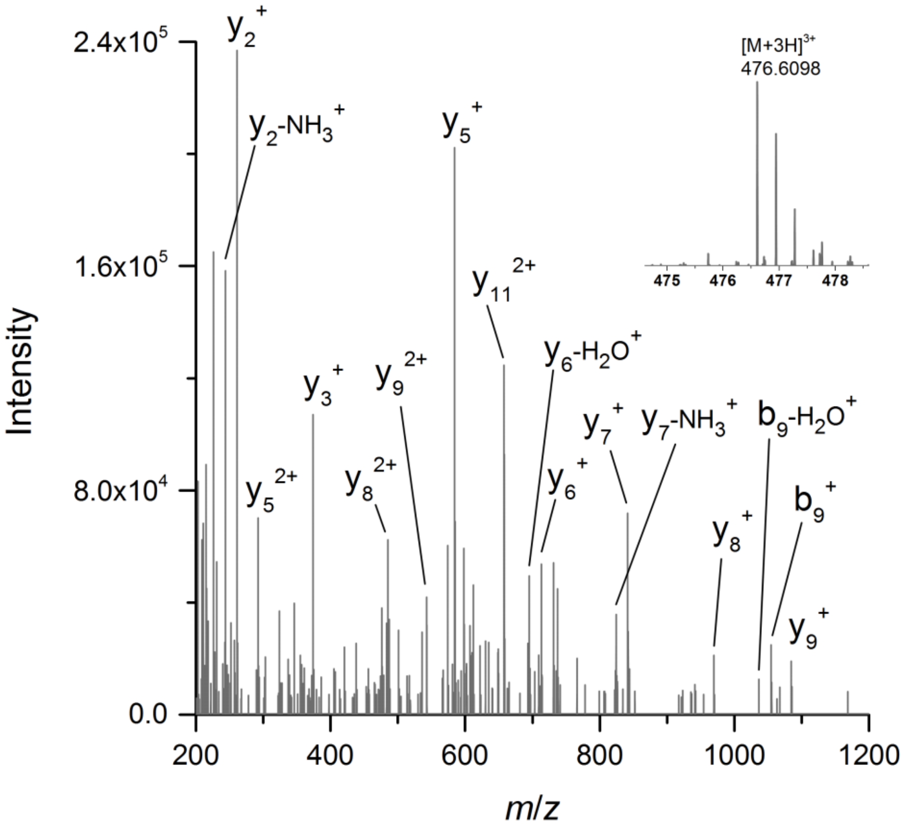

Supplemental Figure S3.29

FQITTSPGSTQK

Location: aa 395-406

Precursor Charge State: 2

Theoretical Monoisotopic: 647.8355 *m/z*

Mass Error: 0.9 ppm

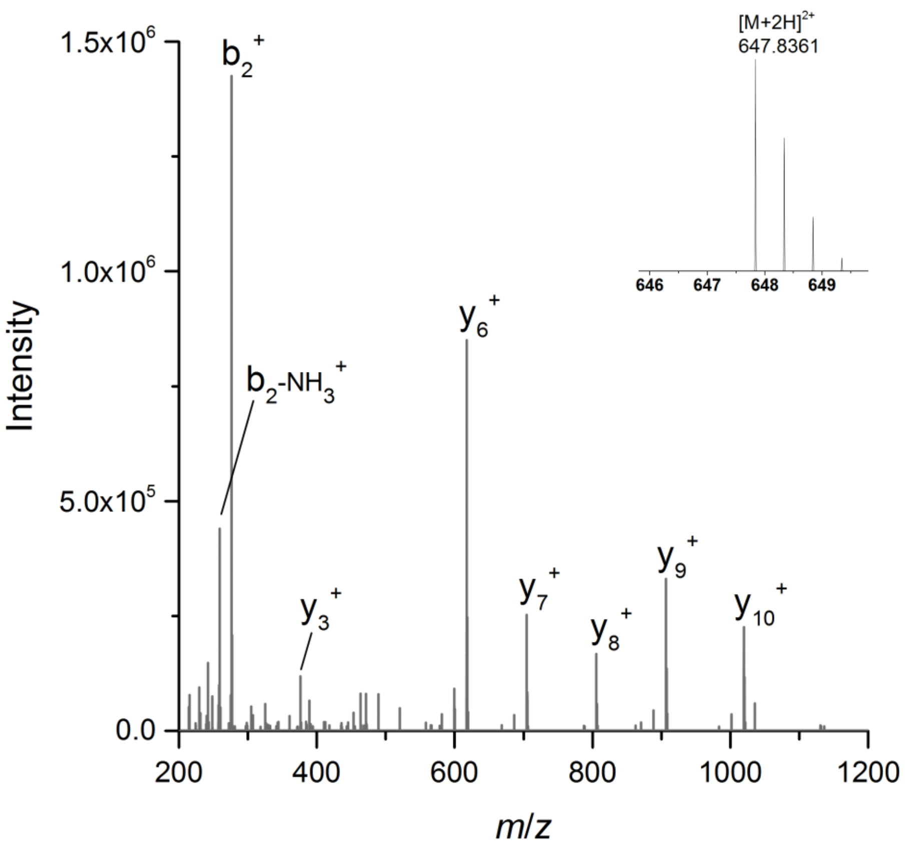

Supplemental Figure S3.30

ILTAMIGLNNK

Location: aa 407-417

Precursor Charge State: 2

Theoretical Monoisotopic: 594.3445 *m/z*

Mass Error: 0.3 ppm

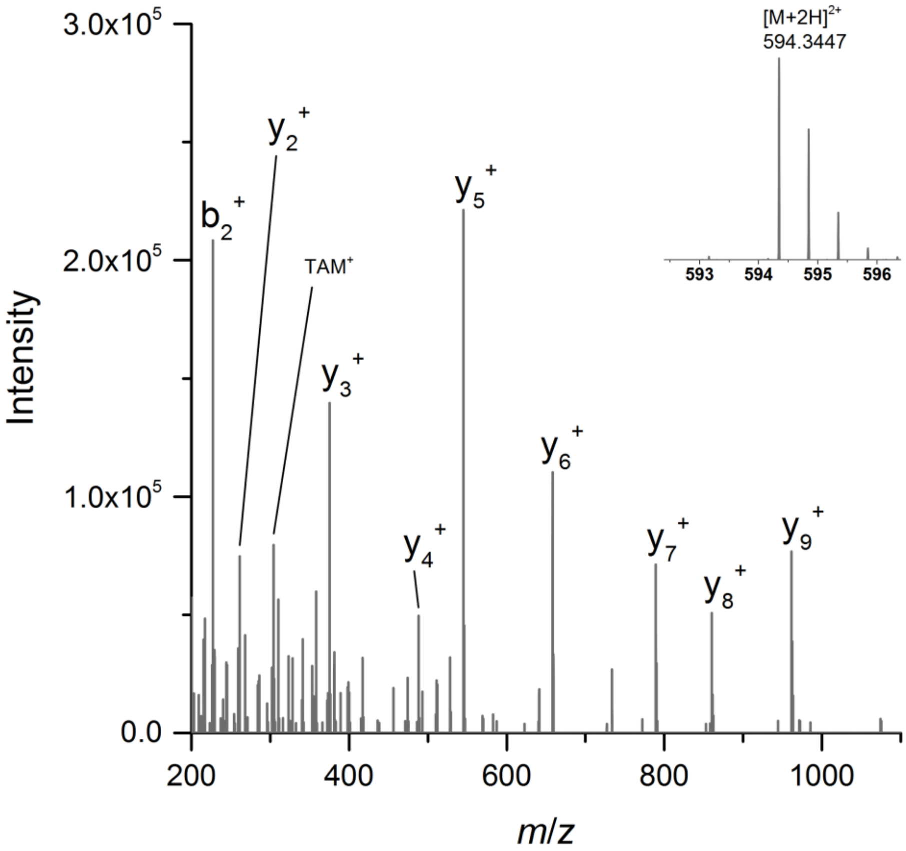

Supplemental Figure S3.31

ILTAmIGLNNK

Location: aa 407-417

Precursor Charge State: 2

Theoretical Monoisotopic: 602.3419 *m/z*

Mass Error: 2.2 ppm

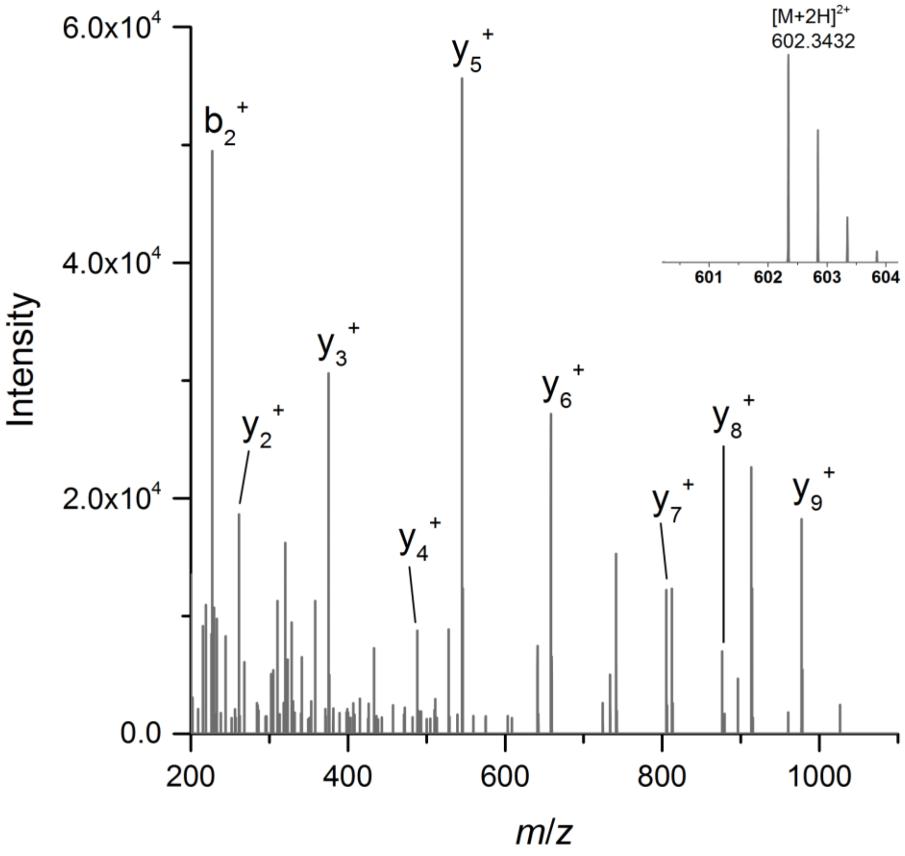

Supplemental Figure S3.32

TLDDKTSYK

Location: aa 418-426

Precursor Charge State: 3

Theoretical Monoisotopic: 357.5170 *m/z*

Mass Error: 2.5 ppm

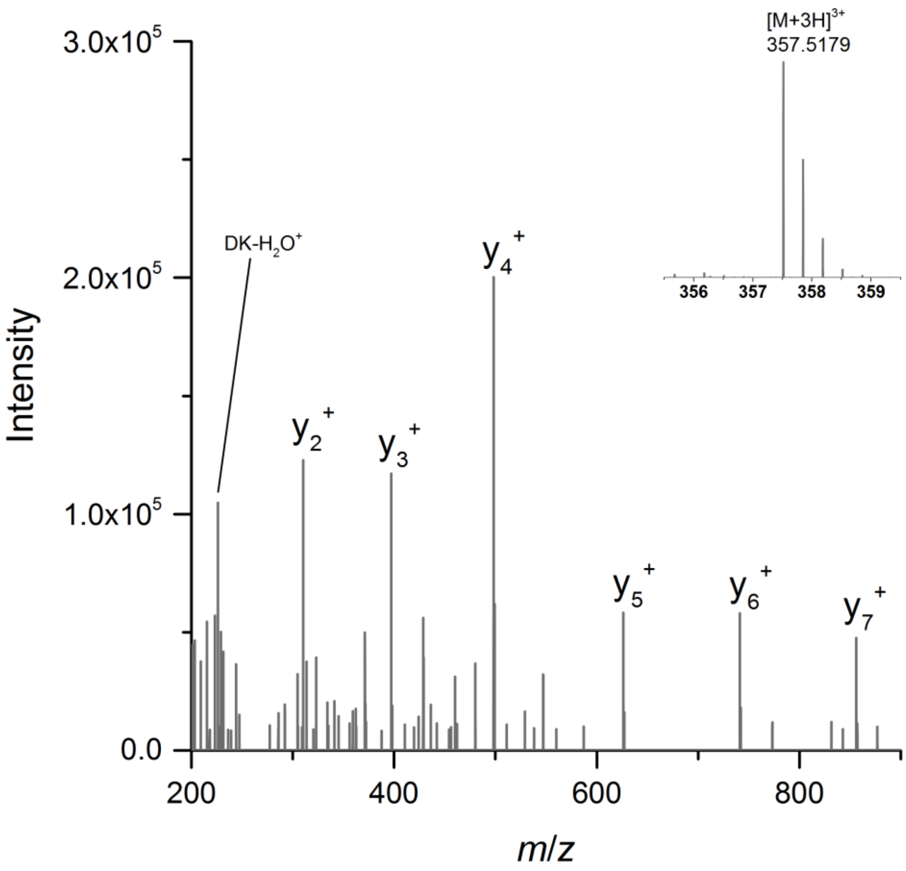

Supplemental Figure S3.33

DKSWGGYNVTR

Location: aa 435-445

Precursor Charge State: 3

Theoretical Monoisotopic: 428.2107 *m/z*

Mass Error: 1.4 ppm

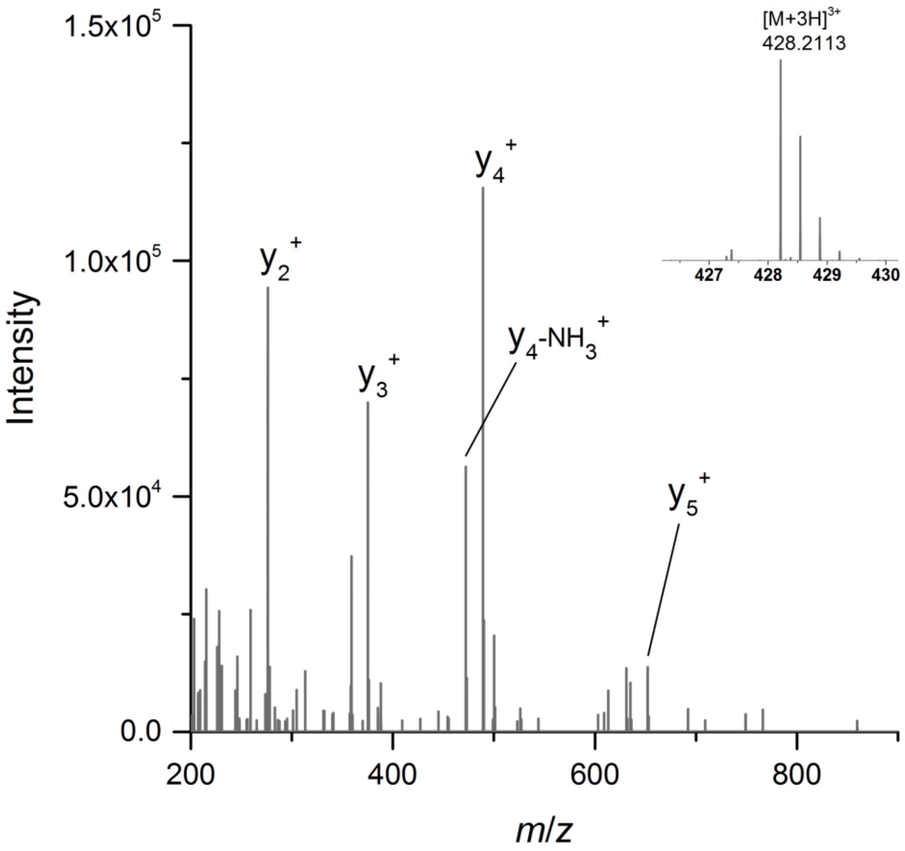

Supplemental Figure S3.34

YEVVNGNIDLK

Location: aa 446-456

Precursor Charge State: 2

Theoretical Monoisotopic: 632.3326 *m/z*

Mass Error: 0.3 ppm

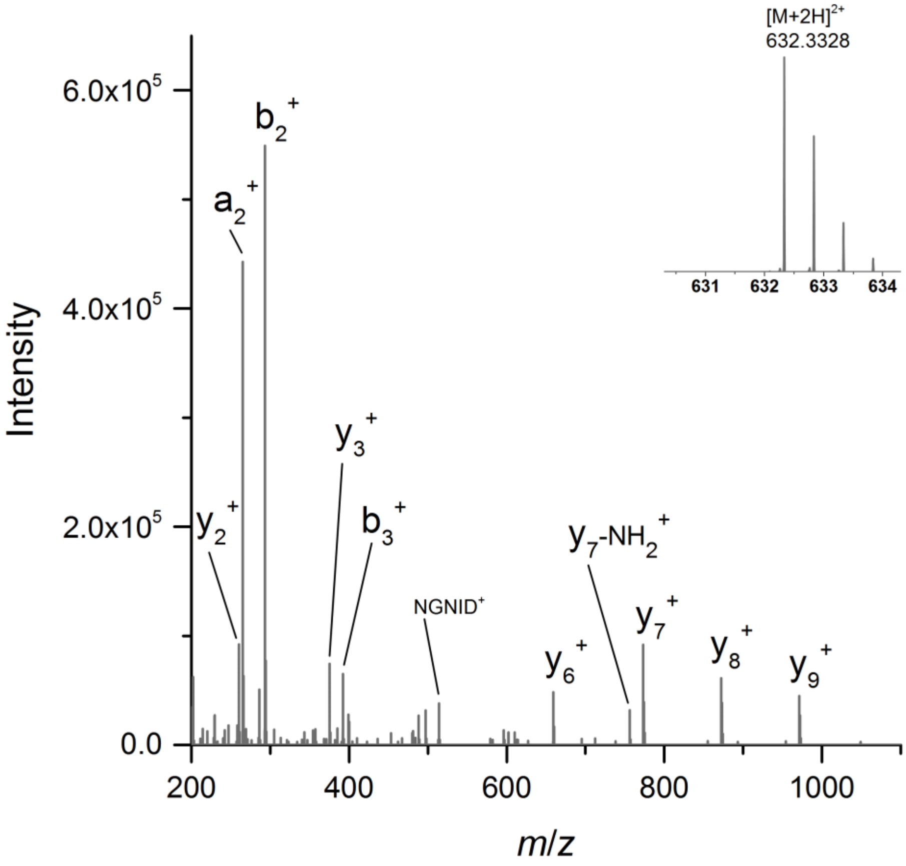

Supplemental Figure S3.35

QAIESSDNIFFAR

Location: aa 457-469

Precursor Charge State: 2

Theoretical Monoisotopic: 749.3703 *m/z*

Mass Error: 2.3 ppm

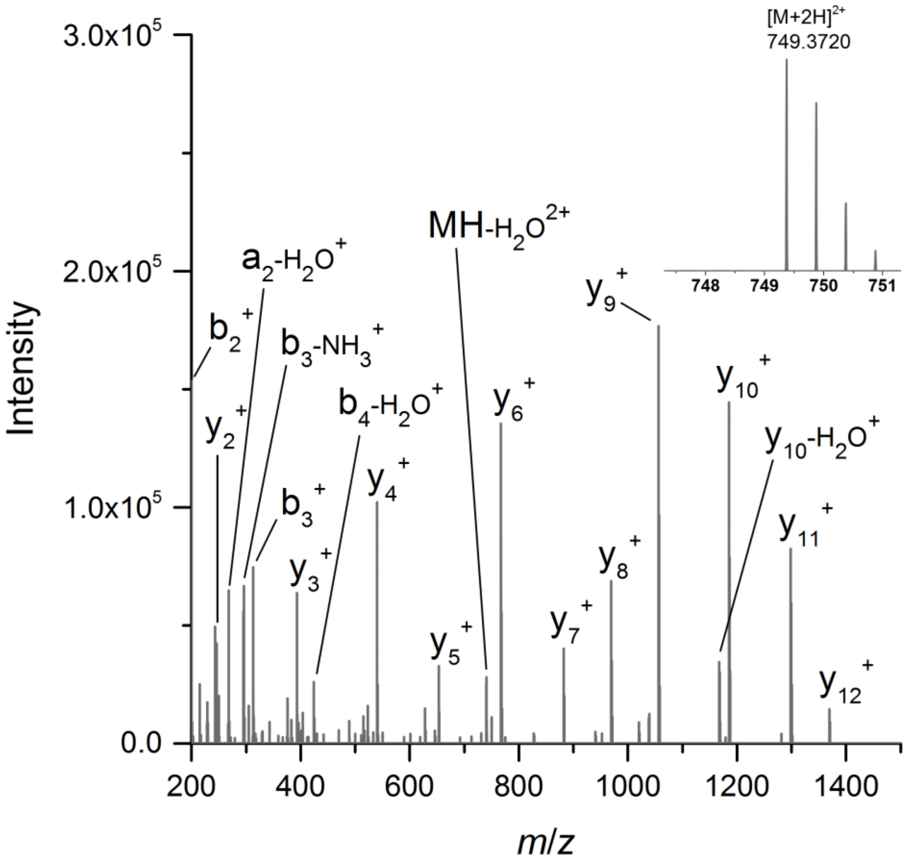

Supplemental Figure S3.36

VALELGSK

Location: aa 470-477

Precursor Charge State: 2

Theoretical Monoisotopic: 408.7449 *m/z*

Mass Error: 2.2 ppm

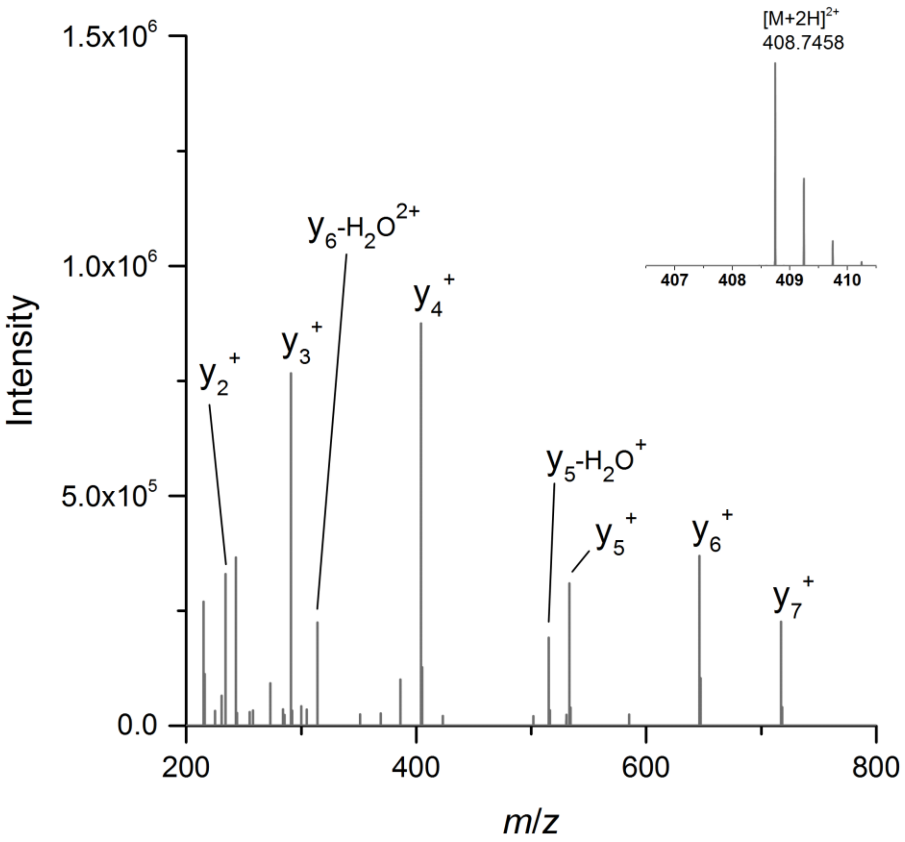

Supplemental Figure S3.37

KLGVGEDIPSDYPFYNAQISNK

Location: aa 485-506

Precursor Charge State: 3

Theoretical Monoisotopic: 819.0762 *m*/*z*

Mass Error: 1.9 ppm

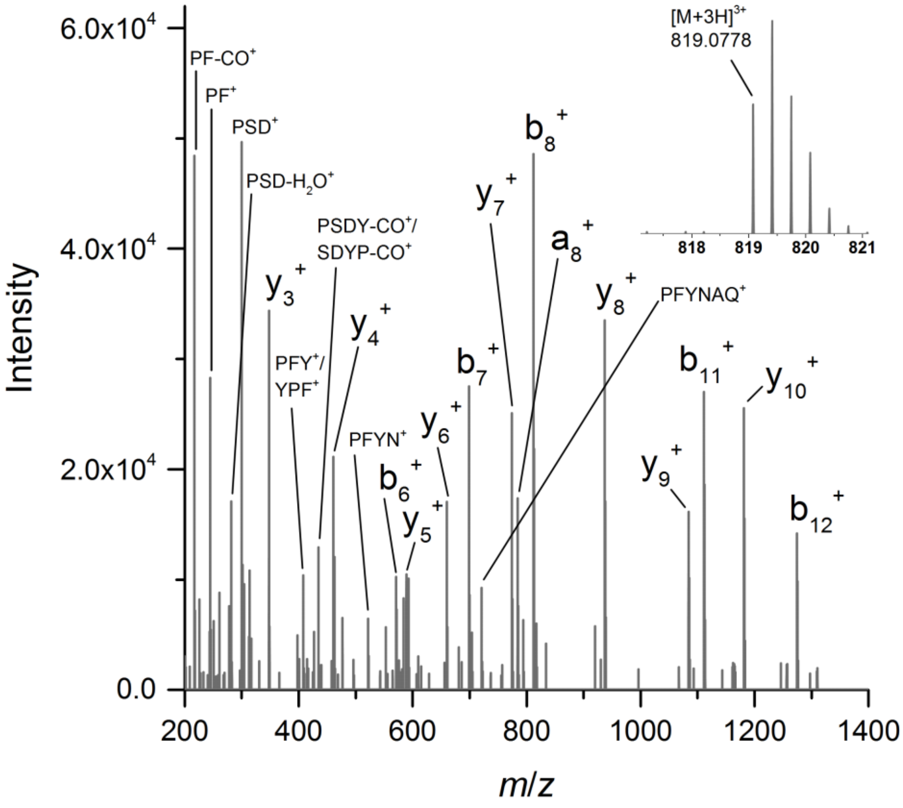

Supplemental Figure S3.38

LGVGEDIPSDYPFYNAQISNK

Location: aa 486-506

Precursor Charge State: 2

Theoretical Monoisotopic: 776.3779 *m/z*

Mass Error: 2.2 ppm

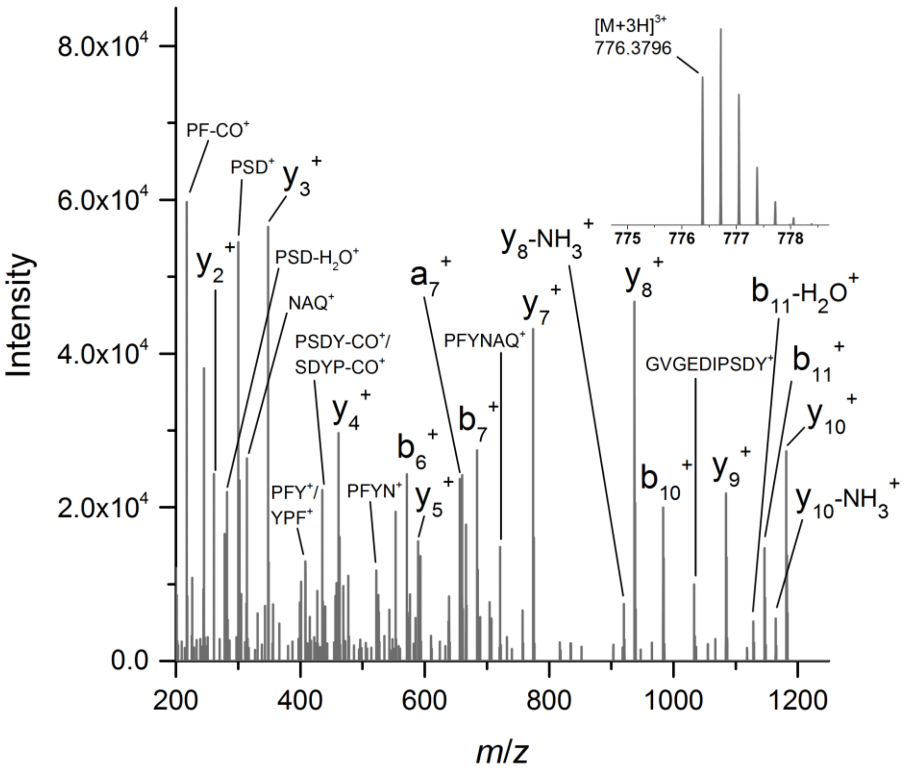

Supplemental Figure S3.39

NKNLDNEIL (Proteinase K)

Location: aa 505-513

Precursor Charge State: 2

Theoretical Monoisotopic: 536.7853 *m/z*

Mass Error: 1.1 ppm

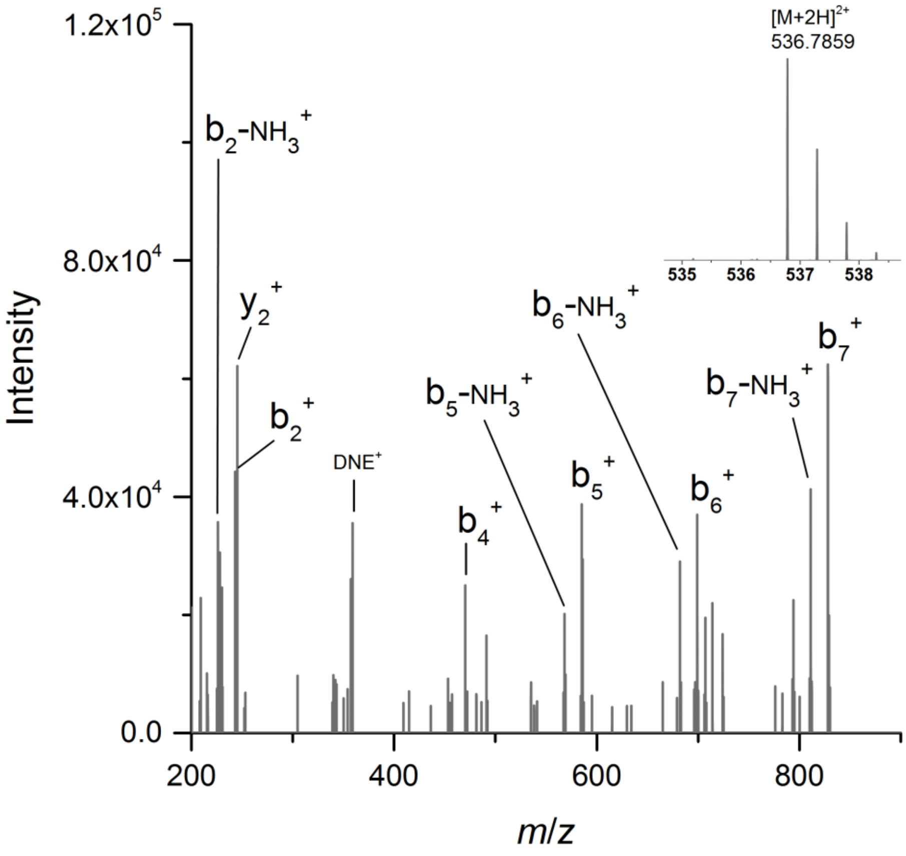

Supplemental Figure S3.40

NLDNEILLADSGYGQGEILINPVQILSIYSALENNGNINAPHLLK

Location: aa 507-551

Precursor Charge State: 3

Theoretical Monoisotopic: 1620.8521 *m/z*

Mass Error: 2.8 ppm

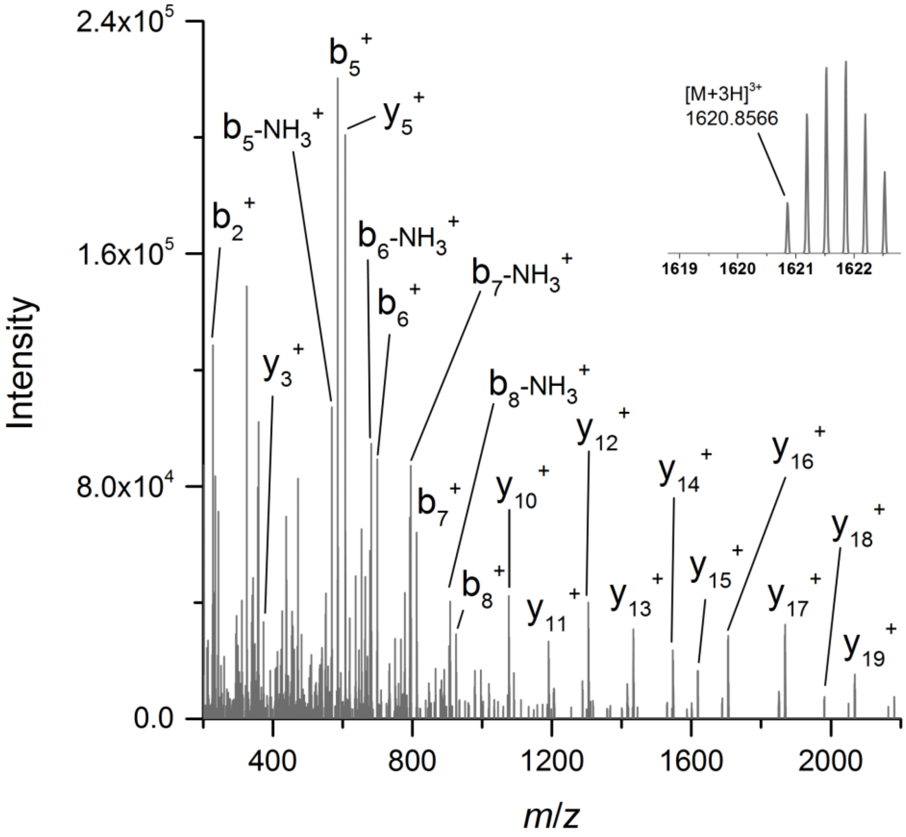

Supplemental Figure S3.41

THKEDIYRSY (Proteinase K)

Location: aa 582-589
Precursor Charge State: 3
Theoretical Monoisotopic: 437.8824 *m/z*
Mass Error: 1.8 ppm

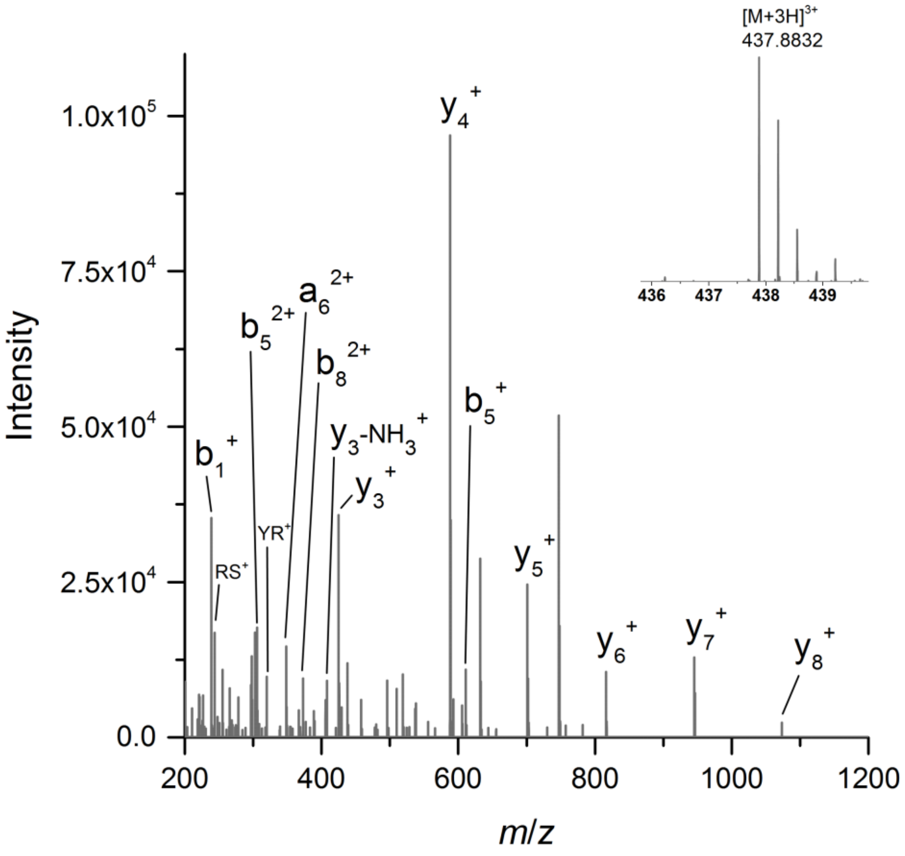

Supplemental Figure S3.42

THKEDIYR

Location: aa 582-589

Precursor Charge State: 3

Theoretical Monoisotopic: 354.5173 *m/z*

Mass Error: 1.4 ppm

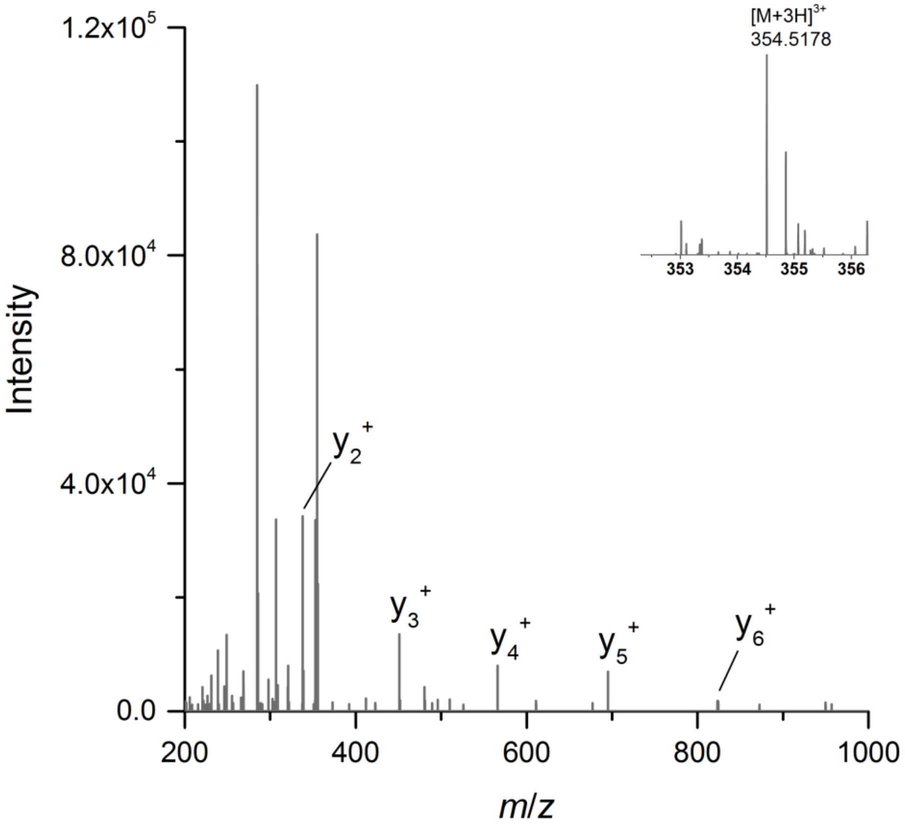

Supplemental Figure S3.43

HKEDIYRSY (Proteinase K)

Location: aa 583-591

Precursor Charge State: 3

Theoretical Monoisotopic: 404.1999 *m/z*

Mass Error: -0.2 ppm

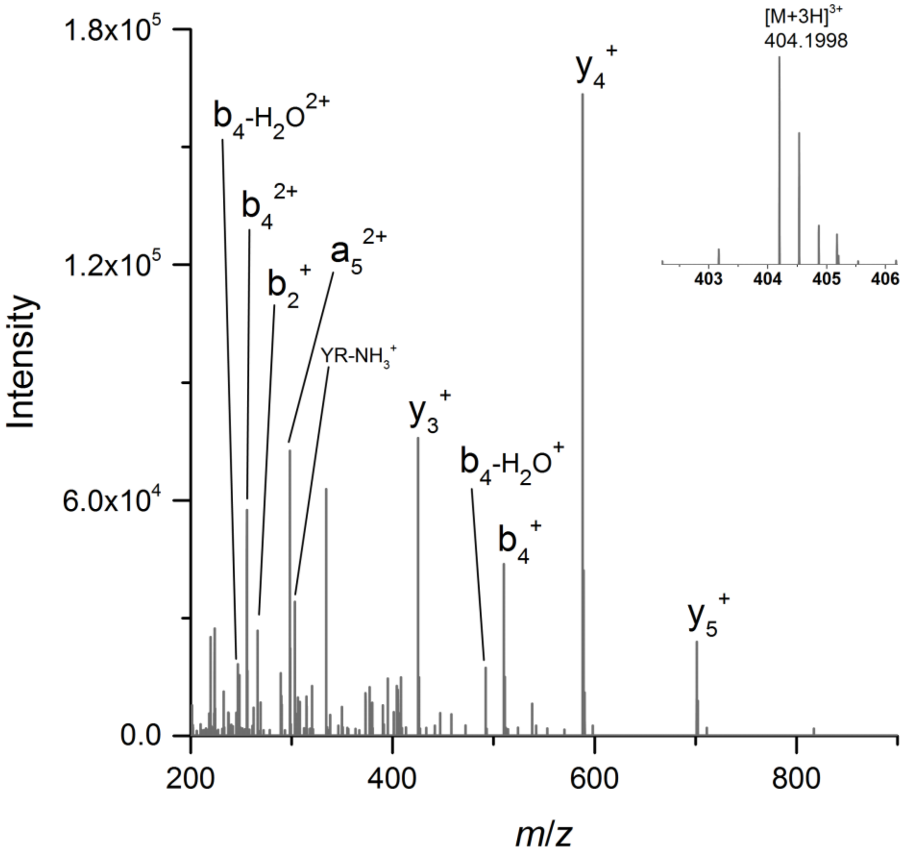

Supplemental Figure S3.44

SYANLIGK

Location: aa 590-597

Precursor Charge State: 2

Theoretical Monoisotopic: 433.2425 *m/z*

Mass Error: 3.9 ppm

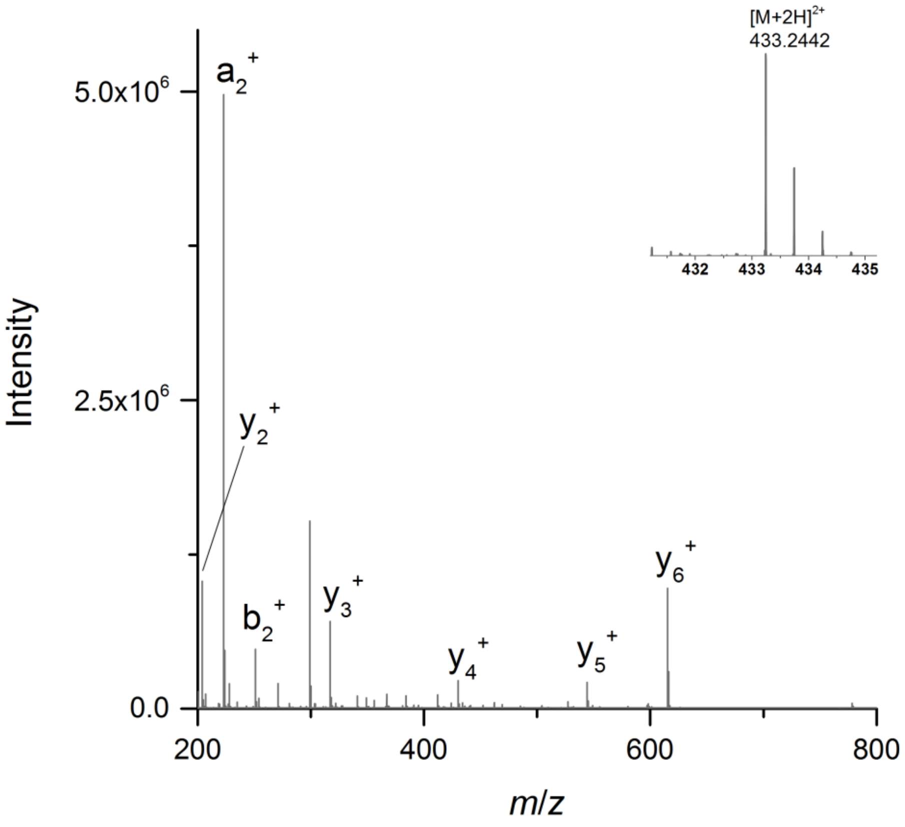

Supplemental Figure S3.45

SGTAELK

Location: aa 598-604

Precursor Charge State: 2

Theoretical Monoisotopic: 353.1925 *m/z*

Mass Error: -0.6 ppm

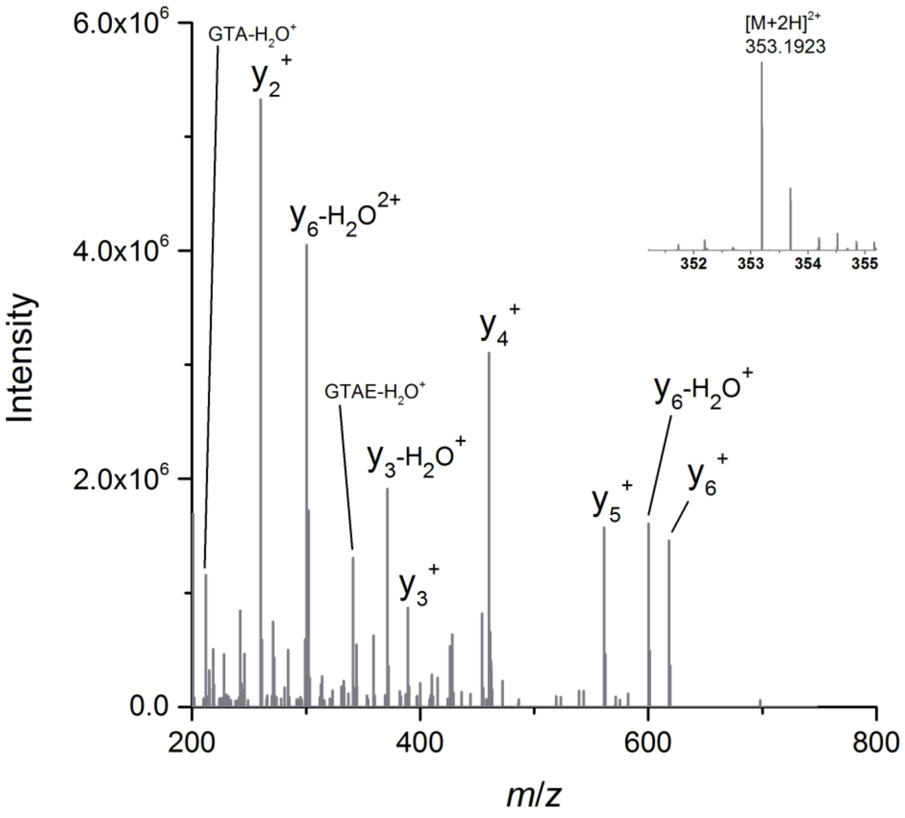

Supplemental Figure S3.46

QIGWFISYDKDNPNmMMAINVK

Location: aa 613-634

Precursor Charge State: 3

Theoretical Monoisotopic: 877.7517 *m/z*

Mass Error: -0.1ppm

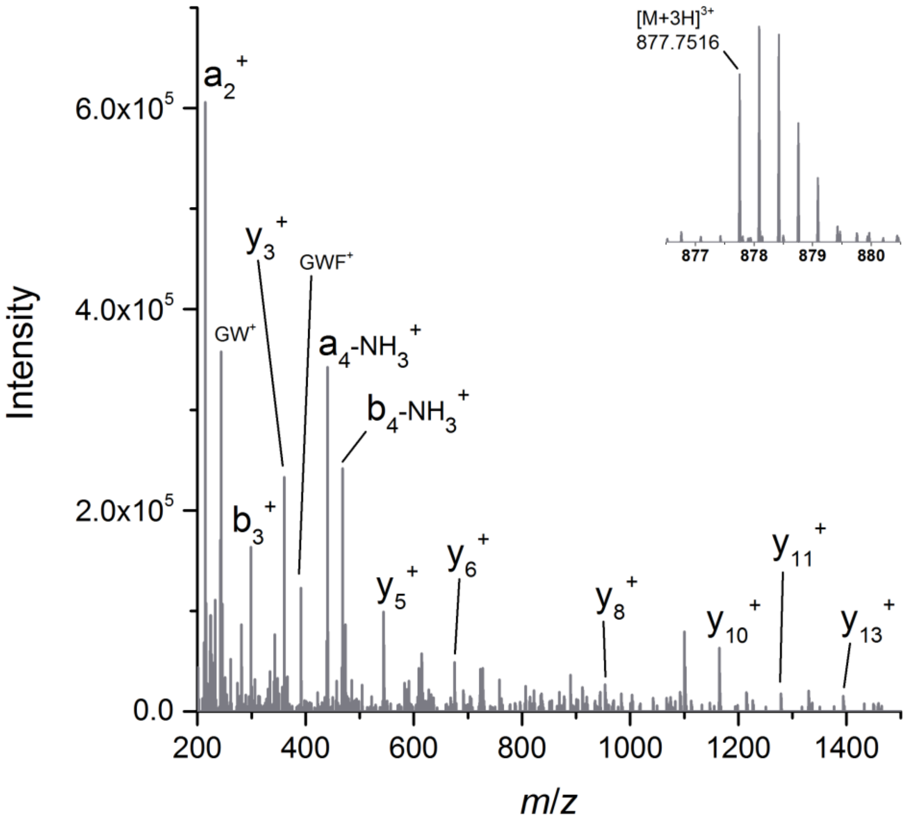

Supplemental Figure S3.47

ISYDKDNPNMM (Proteinase K)

Location: aa 618-628

Precursor Charge State: 2

Theoretical Monoisotopic: 664.2865 *m/z*

Mass Error: 2.0 ppm

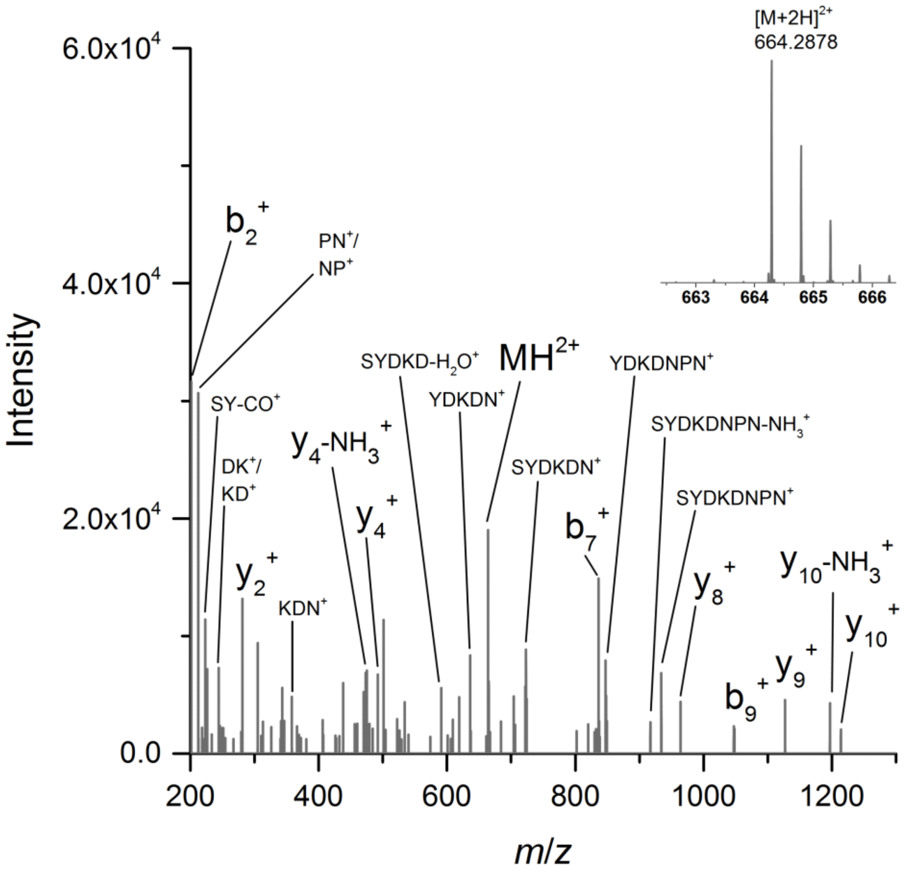

Supplemental Figure S3.48

YDKDNPNMM (Proteinase K)

Location: aa 620-628

Precursor Charge State: 2

Theoretical Monoisotopic: 564.2284 *m/z*

Mass Error: 2.0 ppm

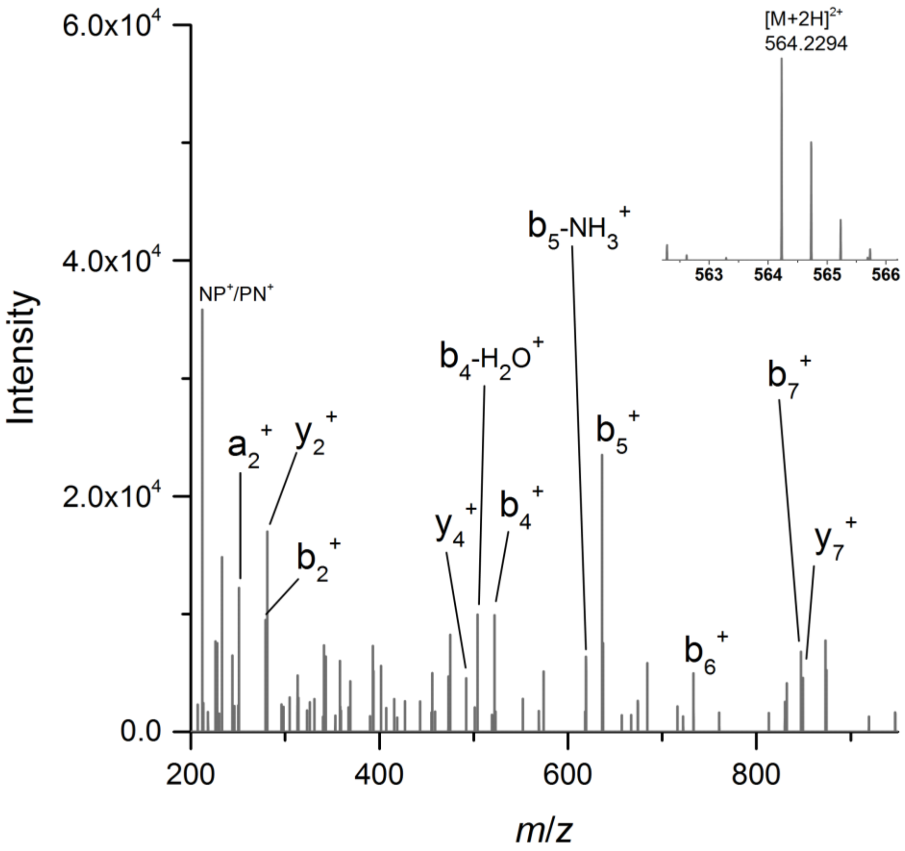

Supplemental Figure S3.49

DKDNPNMM (Proteinase K)

Location: aa 621-628

Precursor Charge State: 2

Theoretical Monoisotopic: 482.6968 *m/z*

Mass Error: 0.8 ppm

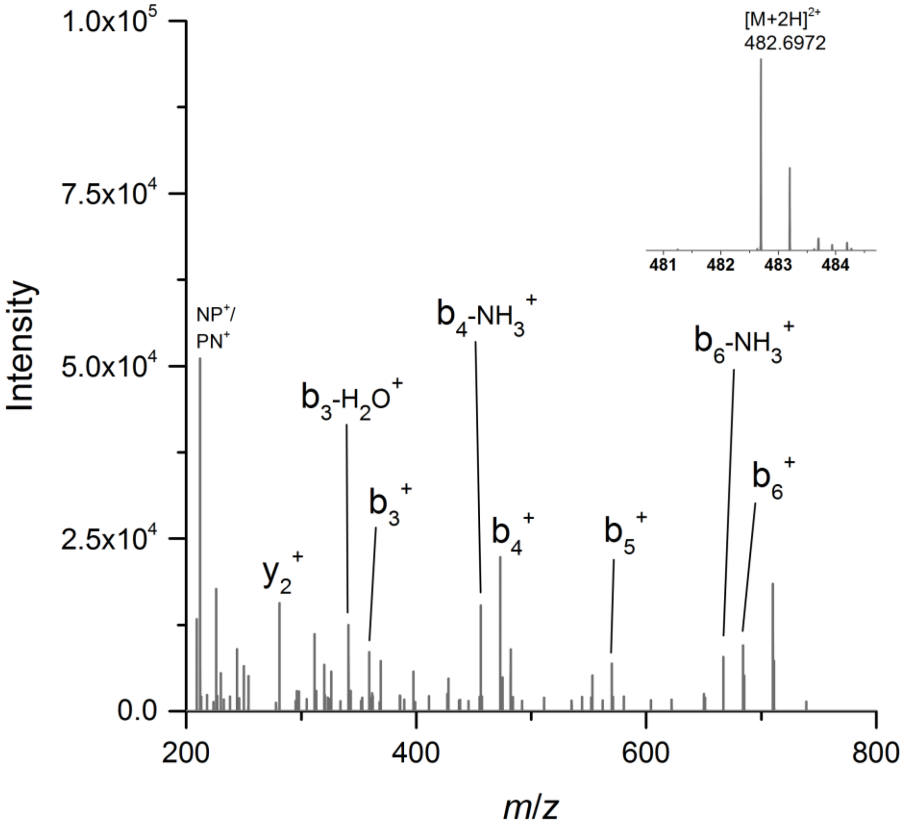

Supplemental Figure S3.50

GMASYNAK

Location: aa 640-647
Precursor Charge State: 2
Theoretical Monoisotopic: 421.1973 *m/z*
Mass Error: -0.7 ppm

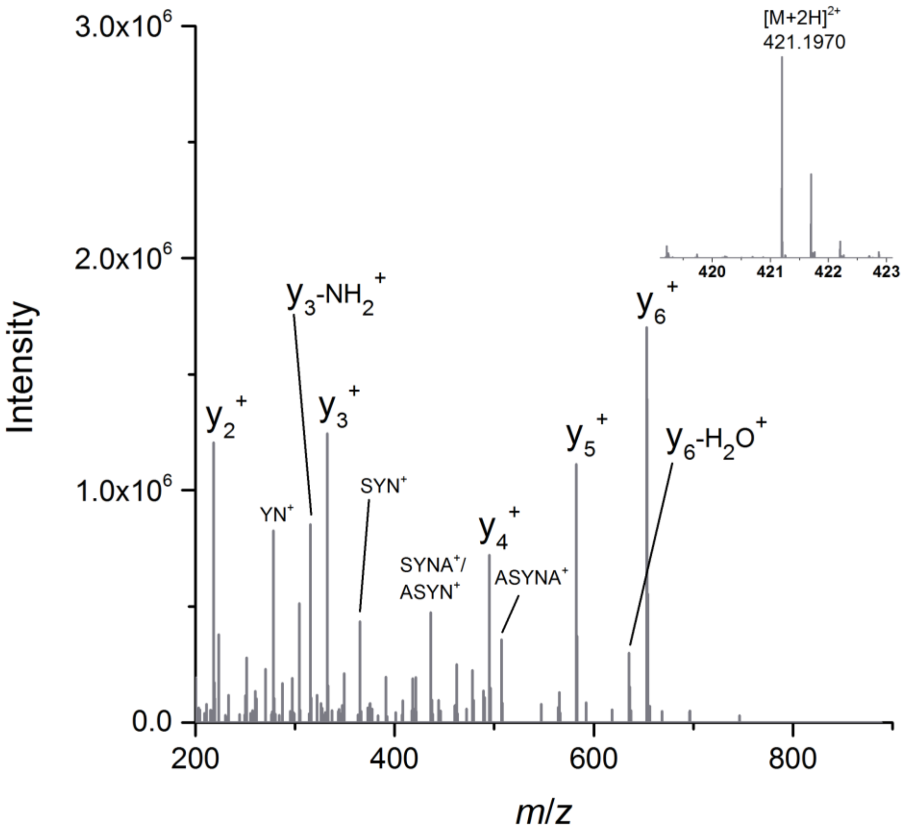

Supplemental Figure S3.51

VYDELYENGNK

Location: aa 652-662

Precursor Charge State: 2

Theoretical Monoisotopic: 672.3093 *m*/*z*

Mass Error: 1.8 ppm

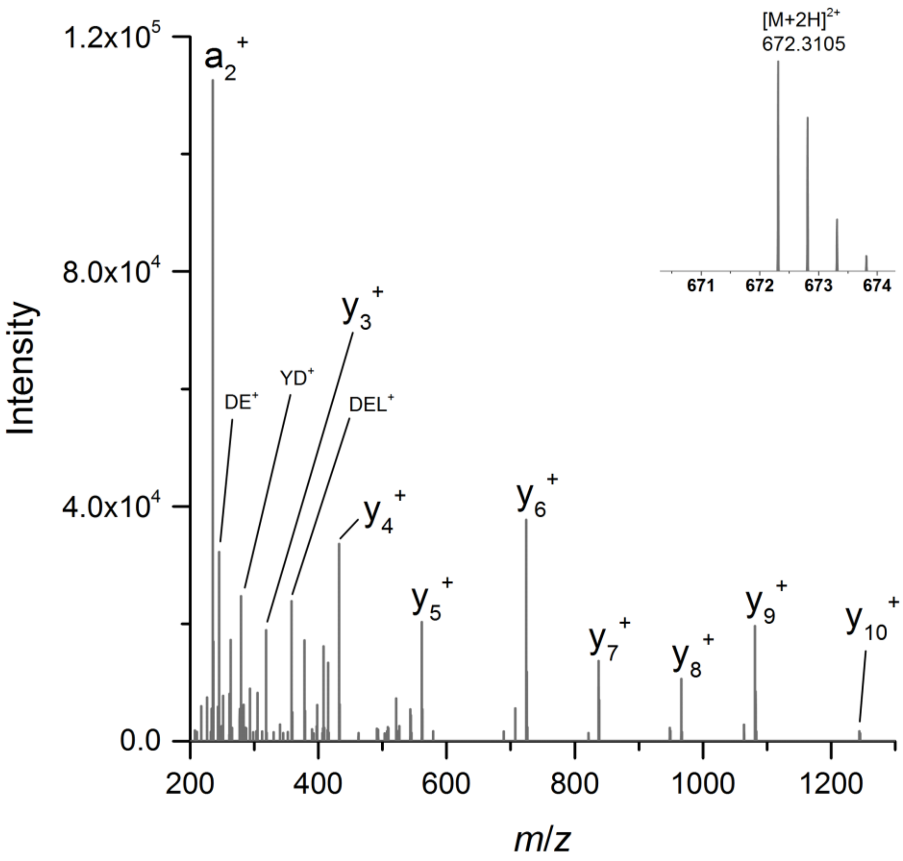

Supplemental Figure S3.52

KYDIDE

Location: aa 663-668

Precursor Charge State: 2

Theoretical Monoisotopic: 391.6820 *m/z*

Mass Error: -0.5 ppm

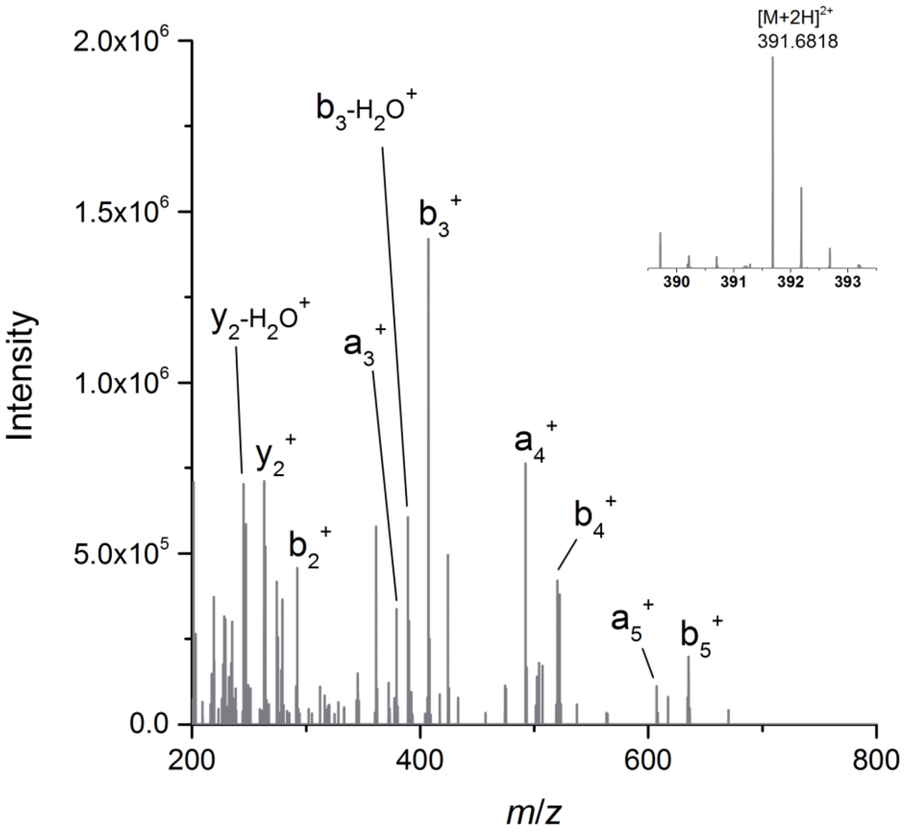

Supplemental Figure S4.

Representative illustration of cyclic peptide rearrangement of source-induced PBP2a$_{mecA}$ N-terminal peptide for precursor *m/z* = 653.4382.

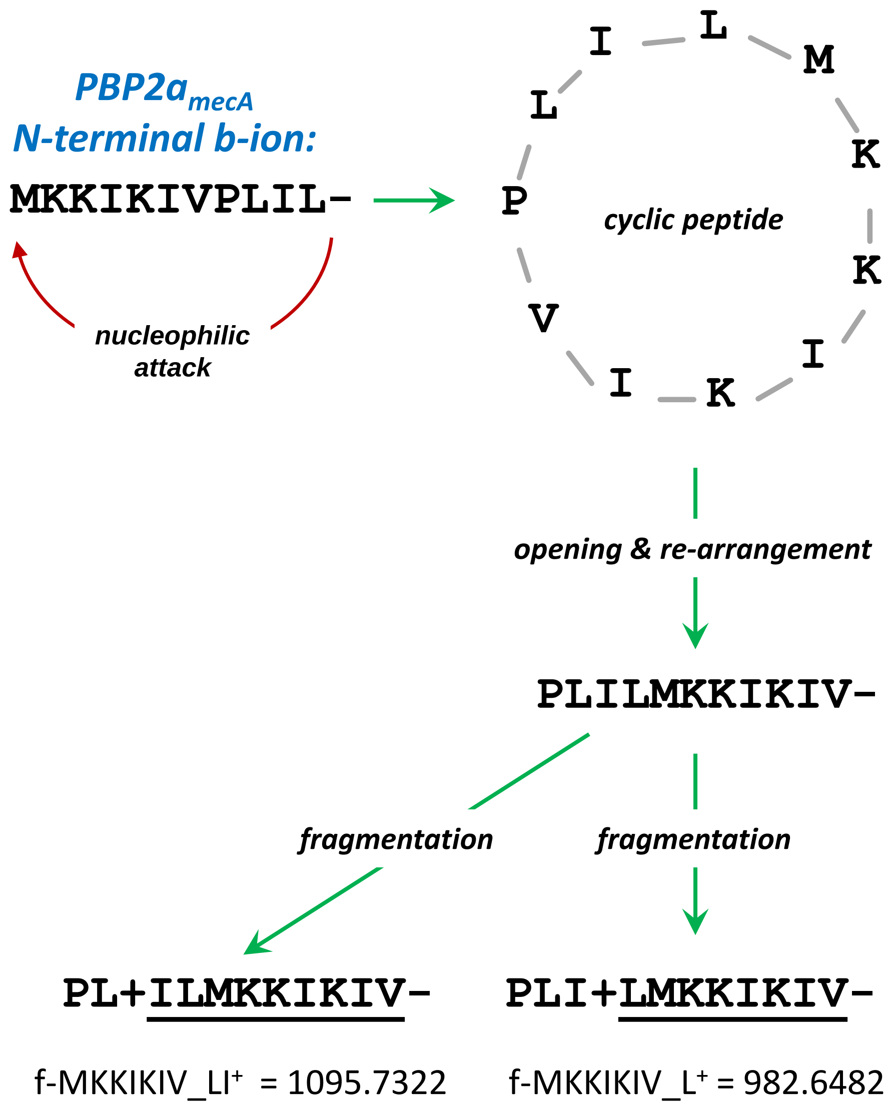

Supplemental Figure S5.

Representative elution profile for $PBP2a_{mecA}$ as indicated by the extracted ion chromatogram for the *m*/*z* 982.6482 transition ion (fMKKIKIV_L^+) for both source-induced peptide-like targets. This analysis was performed following $PBP2a_{mecA}$ enrichment and separation performed on the ProSwift RP-4H at 200 μL/min.

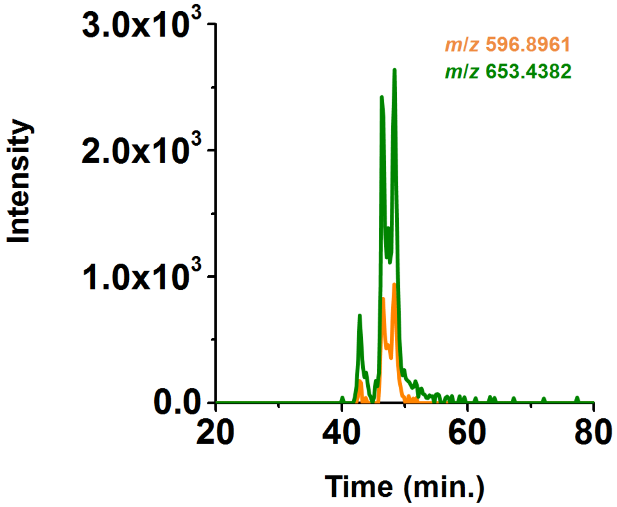

Supplemental Figure S6.

Representative illustration of cyclic peptide rearrangement of source-induced PBP2a$_{mecC}$ N-terminal peptide for precursor *m/z* = 715.4462.

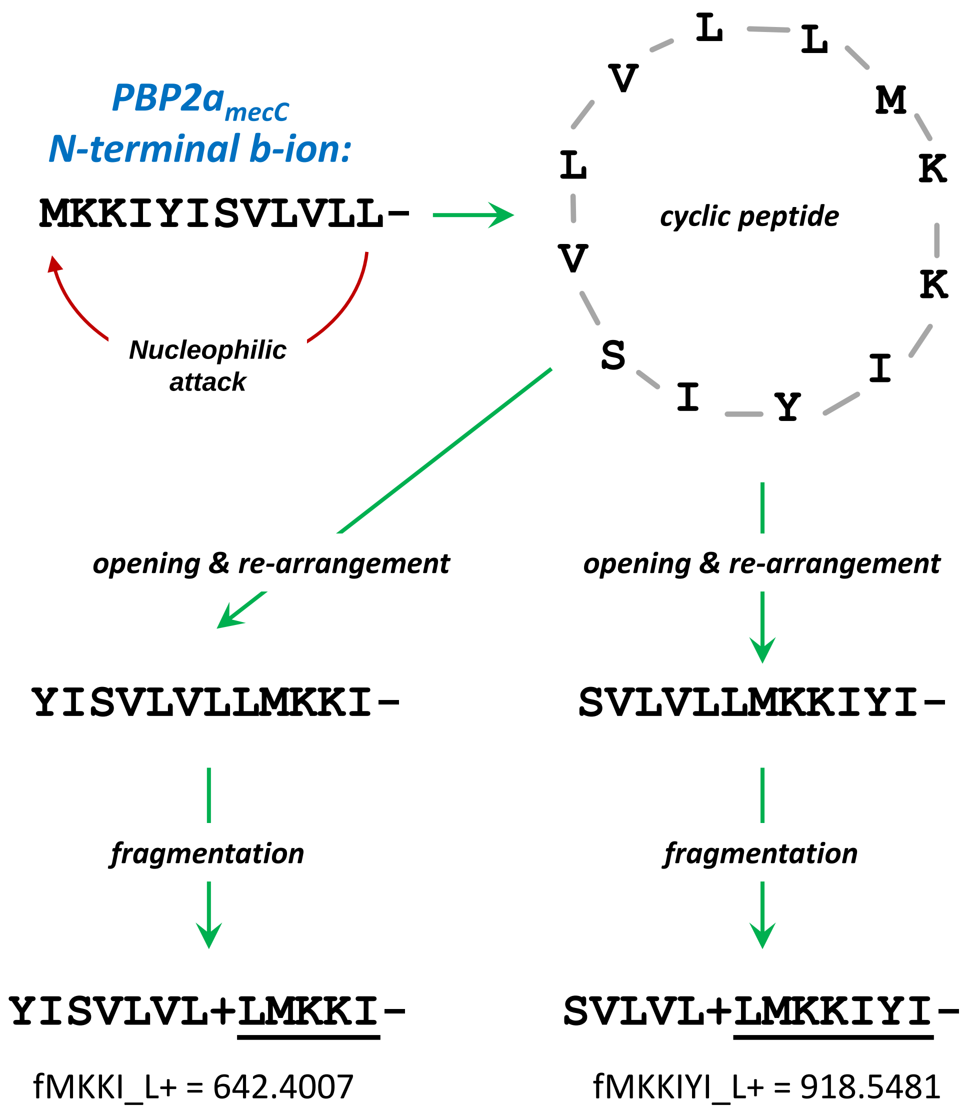

Supplemental Figure S7.

Cross sectional display of reverse phase monolith SPE tip.

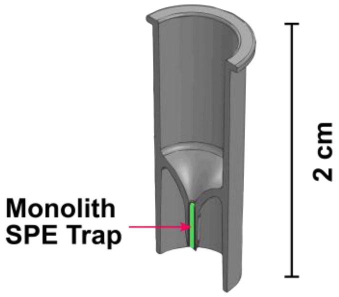

Supplemental Figure S8.

Representative elution profile for PBP2a$_{mecA}$ on the monolith solid phase extraction tip as illustrated by extracted ion chromatogram for the *m/z* 982.6482 transition ion (fMKKIKIV_L^{+}) for both source-induced peptide-like targets. Acetonitrile gradient is indicated by the blue line. Protein separation was performed at 3 μL/min.

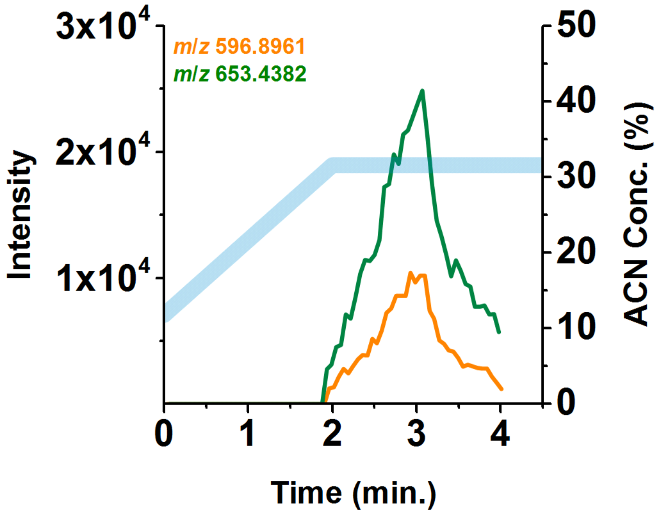

Supplement: Supplementary file 1 — Supplementary Information. [file 41598_2021_97844_MOESM1_ESM.pdf]
